# Unveiling the Electrolyte and Solid Electrolyte Interphase in Sodium Ion Batteries: Mechanisms, Progress, and Perspectives

**DOI:** 10.1002/adma.202510882

**Published:** 2025-10-10

**Authors:** Meng Li, Chengzhi Sun, Ruoqi Zhang, Man Qi, Zeen Wu, Xin Zhang, Yu Zhang, Jiayin Yuan, Naiqing Zhang

**Affiliations:** ^1^ State Key Laboratory of Urban‐rural Water Resource and Environment School of Chemistry and Chemical Engineering Harbin Institute of Technology Harbin 150001 China; ^2^ Department of Chemistry Stockholm University Stockholm 10691 Sweden; ^3^ Weichai Power Co., Ltd. Weifang Shandong 261061 China

**Keywords:** electrolyte, mechanism, progress, sodium ion batteries, solid electrolyte interphase

## Abstract

Sodium‐ion batteries (SIBs) are regarded as a promising alternative to lithium‐ion batteries due to the low cost and abundant availability of sodium. Electrolyte, as the medium for ion transport, plays a crucial role in determining the electrochemical performance. Currently, SIBs employ mainly organic electrolytes, aqueous electrolytes, ionic liquids, gel electrolytes, and solid electrolytes. These electrolytes have made significant progress according to the needs of various application scenarios. Notably, the solid electrolyte interphase (SEI) formed by decomposition of electrolytes on the electrode surface has a decisive influence on the performance of SIBs, and its composition and formation mechanism are closely related to the chemical nature of the electrolyte. Therefore, a deep understanding of the structure and interfacial chemistry of the SEI is essential for developing high‐performance SIBs, preferably through the simple and effective modulation of electrolyte composition. However, the fragmented and insufficient mechanistic summary on this connection results in poor guidance on future research, especially for the co‐design of electrolyte and solid electrolyte interphase. This review summarizes and compares the research progress of various electrolyte systems, discusses the formation and aging mechanisms of SEI, and presents the perspectives on the integrated design of electrolyte and SEI.

## Graphical abstract

This review summarizes the recent advances in sodium‐ion battery electrolytes (including organic, aqueous, gel, ionic liquid, and solid‐state types) and their influence on the solid electrolyte interphase (SEI). It discusses SEI formation, aging, and optimization, as well as cases where no SEI forms, providing insights into the critical relationship between electrolyte chemistry and interfacial stability.

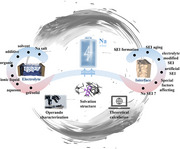

## 1 Introduction

**Figure 1:**
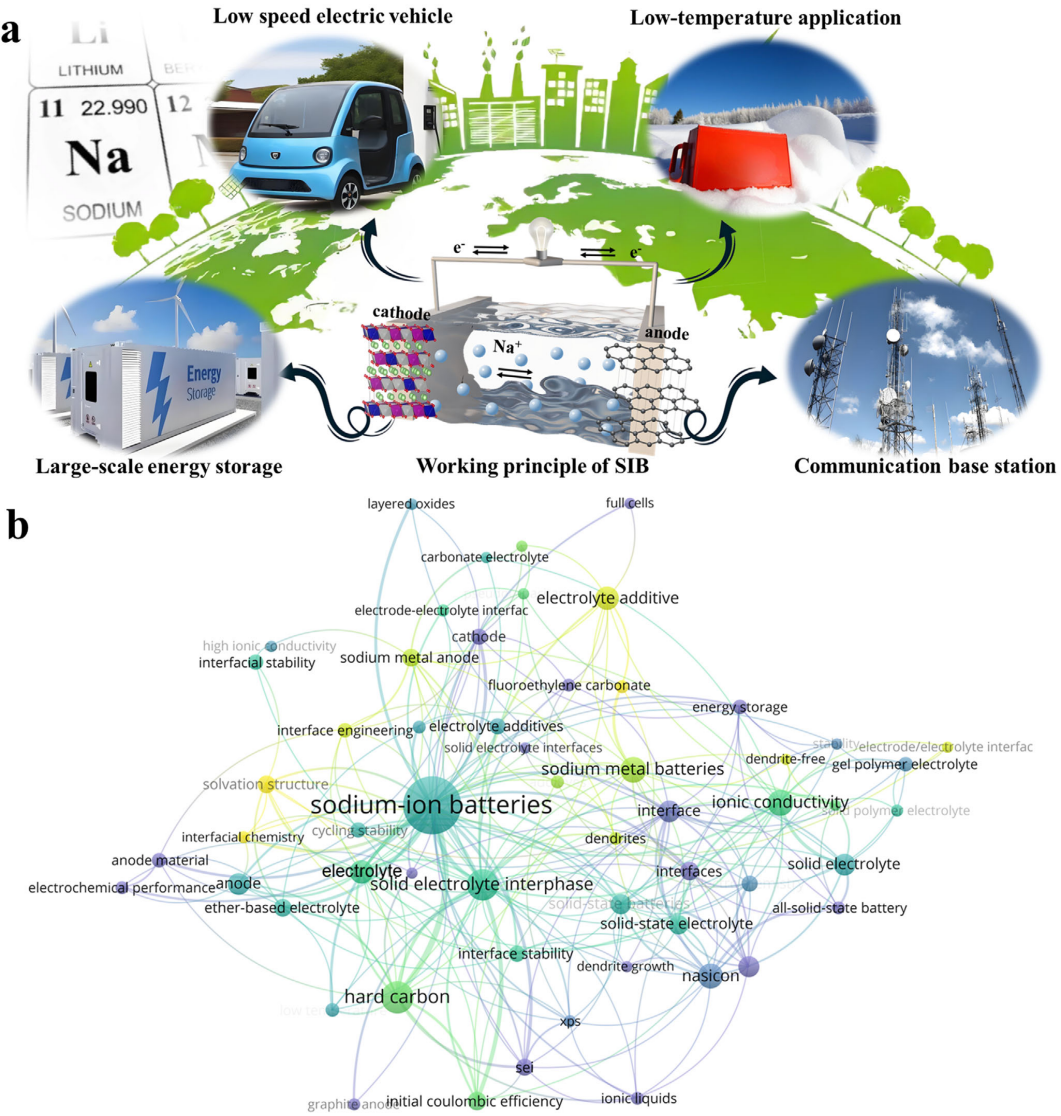
a) Working principle and application scenarios of SIBs. b) Research hotspots retrieved using SIB electrolyte and interface as keywords, data from Web of Science.

In order to overcome the pressing issues of global warming and environmental pollution caused dominantly by fossil fuel combustion, clean and renewable energy sources have been vigorously promoted to partially, if not fully, replace conventional primary energy sources. Accompanied by these efforts, highly efficient and sustainable electrochemical energy storage (EES) technologies are essential for meeting the use of renewable energy at scale.^[^ ^1^, ^2^ ^]^ Lithium‐ion batteries (LIBs) have been widely employed in consumer electronics and power batteries, and are expanding into the EES market, due to their high energy density, fast response, lightweight, and compactness.^[^ ^3^, ^4^ ^]^ However, problems associated with the high cost and limited lithium resource make it debatable, considering the rapid expansion of EES. Sodium ion batteries (SIBs) share a similar working principle as LIBs, and are gaining great attention as an alternative to LIBs in EES because of the low cost and practically unlimited sodium resources, meeting a wide range of practical scenarios^[^ ^5^, ^6^, ^7^ ^]^ (**Figure** **1**a).

As the core carrier of ion transport, the physicochemical properties of the electrolyte directly affect the electrochemical performance of SIBs. Although current electrolytes for SIBs mainly follow that of LIBs, including organic electrolytes, aqueous electrolytes, ionic liquids, gel electrolytes, and solid electrolytes,^[^ ^8^, ^9^ ^]^ the larger ionic radius and distinct solvation energy of sodium ions in solution suggest that the design of SIB electrolytes requires a varied structure‐property relationship. On the one hand, the easier desolvation of sodium ions affords SIB electrolytes to exhibit faster ion transport kinetics and better low‐temperature performance. On the other hand, the larger ionic radius imposes greater lattice stress on electrode materials, which can undermine interfacial stability.^[^ ^10^, ^11^, ^12^ ^]^ In addition, as new materials chemistries emerge at the electrode, the electrolyte will need to evolve further to ensure optimal compatibility and performance. At present, ester electrolytes are the most common in commercial LIBs due to their large voltage window and high ion conductivity; they are also used as common organic electrolytes in SIBs due to their better compatibility with electrode materials. Ether electrolytes attract rising interest in SIBs due to their low viscosity and wide working window in the low‐temperature range.^[^ ^13^, ^14^ ^]^ Besides, non‐flammable ionic liquid electrolytes, aqueous electrolytes, gel electrolytes, and solid electrolytes are starting to receive more and more attention owing to growing safety concerns.

As SIB technology matures, substantial progress has been made in the application of these diverse electrolyte systems. However, current review articles on SIB electrolytes tend to be fragmented and lack a comprehensive mechanistic understanding. Notably, there is a scarcity of systematic comparisons and generalizations across different electrolyte types. Some reviews focus exclusively on non‐aqueous electrolytes, while others neglect aqueous or solid‐state systems. In addition, the electrolyte will undergo potential‐driven oxidative/reductive decomposition to form a solid electrolyte interphase (SEI) on the electrode. Note that the oxidative decomposition formed a solid electrolyte interphase on the cathode surface, which is sometimes specifically called CEI.^[^ ^15^, ^16^ ^]^ This passivation layer protects the electrode material, ensures its stable operation, and suppresses further decomposition of the electrolyte.^[^ ^17^ ^]^ While challenges, including easy breakage, high interface resistance, continuous consumption of cations,^[^ ^18^, ^19^, ^20^ ^]^ and more soluble than the SEI in LIBs,^[^ ^21^ ^]^ limit the Coulombic efficiency and cycle stability of SIBs. Therefore, the formation mechanism, composition, and morphological structure of SEI are becoming the hotspots due to its crucial role in determining the electrochemical performance of SIBs. These factors of SEI are highly correlated with the chemical nature of the electrolyte.^[^ ^22^ ^]^ Currently, the understanding between SEI and electrolyte is gradually being accumulated based on various advanced characterization techniques and theoretical simulation methods. This knowledge is enriching the interfacial chemistry of SIBs in terms of the ion transport, the desolvation process, electrolyte decomposition, etc. We searched the literature related to SIBs in the database from Web of Science, in which some of the keywords are shown in Figure 1b. It can be seen that topics about electrolytes and SEI have received great attention. However, the existing reviews focus on classifying electrolyte systems and SEI on specific electrode surfaces. A consensus has yet to be reached on the formation and evolution laws of SEI. Overall, the fragmented cognition on electrolytes and SEI leads to poor theoretical guidance on pursuing high‐performance SIBs.

**Figure 2:**
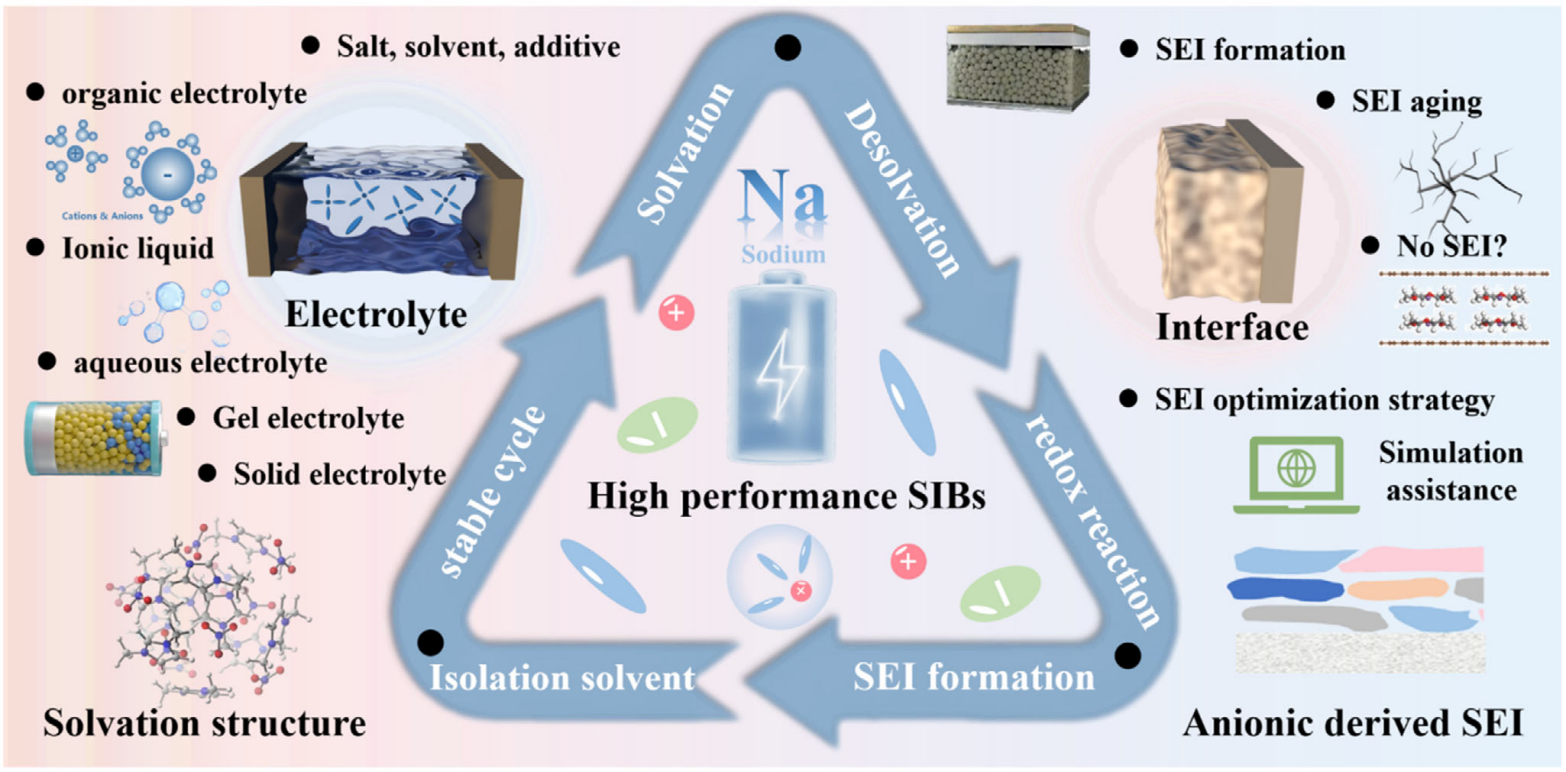
Overview of electrolyte and interface issues discussed in this review.

This review brings four aspects of SIBs connected to electrolyte and SEI together. i) the development history of SIB electrolytes; ii) the research progress of different electrolyte systems; iii) the formation, aging mechanism of SEI and its influence on the interfacial chemistry; iv) the intrinsic tie between electrolytes and derived SEI (**Figure** **2**). Through comprehensive and in‐depth summary, this review aims to delve deeper into the intrinsic connections and mechanisms between electrolytes and interfaces in SIBs, while also offering forward‐looking perspectives through the emerging artificial intelligence (AI) tools.

## 2 SIB Electrolyte Composition

In SIBs, the electrolyte not only serves as a medium for ion transport but also plays a crucial role in balancing and transferring charges between the two electrodes. SIB electrolytes typically consist of three components: sodium salt(s), solvent(s), and additive(s). Different electrolyte systems are primarily distinguished by the type of solvent used, which has a significant impact on battery safety, electrochemical performance, and cost. In this section, we will focus on the fundamental physicochemical properties and basic principles of electrolyte components, with specific electrolyte systems discussed in subsequent sections.

### 2.1 Sodium Salt

Sodium salts are the mainstay of the SIB electrolytes for transporting ions and consist of Na^+^ and anions. In order to ensure high ionic conductivity, sodium salts should have high solubility compatible with common solvents. In addition, Na salt should be highly electrochemical and thermally stable, easily prepared, and non‐toxic and non‐hazardous.^[^ ^23^, ^24^ ^]^ The factors governing these properties of sodium salts are mainly differences in anionic structure. **Table** **1** lists some common sodium salts with their pros and cons.

**Table 1:** Molecular structures and physical properties of common sodium salts and their pros and cons.

Na salt	Anionic structure	Molar mass [g mol^−1^]	Room‐T conductivity [mS cm^−1^]	Melting point [°C]	Advantages	Disadvantages	Refs.
NaPF_6_	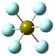	167.95	7.98 (1 m in PC)	300	High conductivity and good electrochemical stability; high compatibility with electrodes	Poor thermal stability, sensitive to water	[40]
NaClO_4_	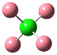	122.44	6.4 (1 m in PC)	468	High conductivity, low cost; insensitive to water	Poor thermal stability; toxic	[40]
NaOTF	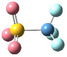	172.06	/	248	High oxidative and thermal stability	Low conductivity; Corroding Al collectors	[40]
NaFSI	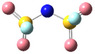	203.12	/	118	High thermal stability; High conductivity; High solubility	Corroding Al collector	[32]
NaTFSI	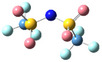	303.13	6.2 (1 m in PC)	257	High thermal stability; High conductivity	High cost; Corroding Al collector	[40]
NaBF_4_	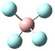	109.79	/	384	High thermal stability; good film‐forming properties	Low ionic conductivity	[23]
NaBOB	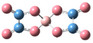	209.84	0.256 (0.025 m)	345	Wide electrochemical window; effective protection of electrodes	High cost; Low solubility	[41]
NaDFOB	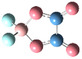	159.8	6.8 (1 m in G2)	/	Good film‐forming properties for improved cycle stability	Difficult and costly to prepare	[42]
NaTDI	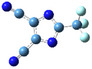	208.1	3.78 (1 m in PC)	/	High conductivity and electrochemical stability	Complex and costly synthesis	[43]
NaPDI	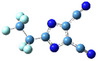	258	3.83 (1 m in PC)	/	High conductivity and electrochemical stability	Complex and costly synthesis	[43]
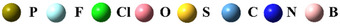							

PF_6_^−^ is the preferential anion for LIBs due to its high conductivity, and suits SIBs as well. NaPF_6_ is currently the “golden standard” of sodium salt in commercial SIB electrolytes. Nevertheless, it is thermally less stable and prone to slow decomposition in contact with water into PF_5_, POF_3_, and HF, which corrode the formed SEI, thus dissolving the cathodic transition metal species, and weakening the cycle stability of the battery.^[^ ^25^, ^26^, ^27^ ^]^ Another common Na salt is NaClO_4_, which matches the electrochemical window of carbonate and has low cost and high stability.^[^ ^28^ ^]^ But it is difficult to dry during use, resulting in a high water content in the prepared electrolyte, which limits its practical use in SIBs. Anions in some ionic liquids form sodium salts with Na^+^, such as sodium trifluoromethanesulfonate (NaOTF),^[^ ^29^ ^]^ sodium bis(fluorosulfonyl)amide (NaFSI), sodium bis(trifluoromethylsulfonyl)imide (NaTFSI), and NaBF_4_. Among them, NaFSI and NaTFSI have high solubility and high conductivity. With the concept of high/localized high concentration electrolytes (HCEs/LHCEs) and weakly solvated electrolytes (WSEs),^[^ ^30^, ^31^ ^]^ these anions have been broadly used due to good film‐forming effect, while the disadvantage is that they are prone to corroding the Al collector.^[^ ^32^ ^]^ Several non‐fluorinated borates such as sodium bis(oxalato)borate (NaBOB),^[^ ^33^ ^]^ sodium‐difluoro(oxalato)borate (NaDFOB),^[^ ^34^ ^]^ sodium bis(salicylato)borate (NaBSB),^[^ ^35^ ^]^ and sodium tetraphenylborate (NaBPh_4_)^[^ ^36^ ^]^ have been used in SIBs, and some of them have quite good performance. Unlike other sodium salts, NaDFOB and NaBOB are more often used as electrolyte additives because of their capability of forming stable SEI, a property that is a “double‐edged sword” because such readily forming SEI may grow too thick to hold a too high impedance. In this context, the amount of the electrode used is a sensitive parameter to control. In comparison, NaBSB and NaBPh_4_ are less researched at present. Here, NaBPh_4_ is known for low oxidative stability, which makes it difficult to match the cathode material.^[^ ^37^ ^]^

The development of new salts aims to meet multiple indicators simultaneously, and can be guided by the following principals: 1) including heterocycles as the backbone structure of anions. For example, sodium 4,5‐dicyano‐2‐(trifluoromethyl) imidazolate (NaTDI) and sodium 4,5‐dicyano‐2‐(pentafluoroethyl)imidazolate (NaPDI), both show high conductivity and good electrochemical stability (>4 V versus Na^+^/Na).^[^ ^38^ ^]^ In addition, these two salts are of high stability against water. 2) Modifying anions through substitution reactions, such as some ─CN‐based substitution salts, e.g., sodium 2,4,5‐tricyanoimidazolate (NaTIM), sodium 2,3,4,5‐tetracyanopirolate (NaTCP), and sodium pentacyanopropenide (NaPCPI). Such salts are highly soluble, thermally stable above 500 °C, oxidatively resistant, and ionically conductive.^[^ ^39^ ^]^

In addition to the sodium salts mentioned above, some exotic anions have been tested for SIB applications. For example, Na[B(hifp)_4_] (hfip = hexafluoroisopropoxy) and Na[B(pp)_2_] (pp = perfluorinated pinacolato) were used in SIBs by Wright et al.^[^ ^44^ ^]^ to possess high resistance to air and water, and superior cyclic stability to NaPF_6_, pointing out commercial promise. Recently, Cui et al.^[^ ^45^ ^]^ reported aluminum fluoride:sodium bis(perfluoropinacolato)aluminate (NaBPPA) as the dominant salt in the electrolyte, which can induce inorganic‐rich SEI at the hard carbon (HC) surface to mitigate the susceptibility to dissolution of SEI in SIBs, and facilitate Na‐storage of HC. To summarize, the development of these new salts has undoubtedly expanded the scope of SIBs. Their properties affect the ion transport and stability of the electrolyte, and in turn, the composition of SEI. While Na salts are of particular interest for SIBs, balancing performance and cost remains a key challenge in transitioning from research to commercial application. This calls for in‐depth analysis and comparison of the properties and mechanisms of various Na salts, as well as continuous exploration of more optimal candidates.

### 2.2 Solvent

The solvent is the component in the highest percentage of the electrolyte, conducting sodium ions between the anode and cathode. The physical and chemical parameters of solvents are paramount for ion transport. In this section, we first discuss their universal parameters, and next the specific types of solvents (such as esters and ethers) in subsequent sections.

In order to facilitate Na^+^ transport in the electrolyte, the electrolyte is required to have a high ionic conductivity, which satisfies the following equation:

(1) $$\sigma =\sum n_{i}u_{i}z_{i}e$$

where *n*, *u*, and *z* are the concentration, mobility, and number of charges of the transported ion. It can be seen that the dissolution of sodium salt in the electrolyte will result in a gradual change in ionic conductivity at different salt concentrations. According to testing results, the maximum ion conductivity is obtained when the salt concentration is ≈1–1.2 m.^[^ ^46^ ^]^ When too much salt is dissolved, ion migration will be affected because of an increase in the viscosity. Current commercial electrolytes are therefore usually set to 1 m.

For fully dissociating the sodium salt, the solvent may have a high dielectric constant, which corresponds to a high polarity. The larger solvent polarity and higher dielectric constant facilitate the salt dissociation, thereby enhancing ionic conductivity. Another key parameter that has been proposed and widely used by researchers is the donor number (DN) of the solvent by Gutmann,^[^ ^47^ ^]^ which measures the capability of a solvent to act as an electron donor. It is highly correlated with the solvation energy and solvation structure of cations. Solvents of high donor numbers typically have stronger binding energies with cations.^[^ ^48^, ^49^ ^]^ The donor number is usually combined with the dielectric constant to determine the solvation capability of a solvent. Different solvents have varied solvation structures, which affect the desolvation process, SEI formation, and cation insertion. In addition, the electrochemical window of the solvent is important to be compatible with cathode and anode materials. For SIBs, the solvent is often required to exhibit high oxidative stability, meaning no decomposition occurs below 4 V. For high‐voltage cathode materials, even higher oxidation resistance is necessary. Density functional theory (DFT) calculations are commonly used to obtain the highest occupied molecular orbital (HOMO) and lowest unoccupied molecular orbital (LUMO) energy levels of solvents, which provide an estimation of their redox behavior. A higher HOMO level indicates a greater tendency toward oxidation, while a lower LUMO level suggests easier reduction.^[^ ^50^ ^]^ However, theoretical calculations serve only as a predictive tool and cannot accurately define the electrochemical window, as actual battery operating conditions play a crucial role. In practice, cyclic voltammetry is often employed to experimentally determine the electrochemical stability of solvents. Moreover, other physical parameters of solvents, such as melting point, boiling point, flash point, and dipole moment, are closely related to the operating temperature of the electrolyte and the electrochemical performance of the cell. An ideal solvent is essential for ensuring a high performance, a long life, and a wide temperature range of the battery. Therefore, when selecting a solvent, it is necessary to consider the above parameters as a whole. **Table** **2** lists the physicochemical properties of some common solvents.

**Table 2:** Molecular structures and physicochemical properties of common solvents.

Solvent	Molecular structure	Boiling point [°C]	Melting point [°C]	Flash point [°C]	Dielectric constant (ε) at 25 °C	Viscosity (η) at 25 °C (cP)	Dipole moment (D) [F m^−1^]	Donor numbers (DN) [kcal mol^−1^]	Refs.
PC	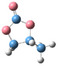	242	−48.8	132	64.92	2.53	5.38	15.1	[51, 52]
EC	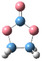	248	36.4	160	89.78	1.9 (40 °C)	5.27	16.4	[52, 53]
EMC	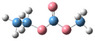	110	−53	23.9	2.958	0.65	0.51	16	[49, 52, 53]
DMC	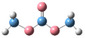	91	4.6	18	3.107	0.59	0.76	17.2	[52]
DEC	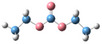	126	−74.3	31	2.805	0.75	0.63	16.0	[51]
DME	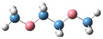	84	−58	0	7.18	0.46	1.15	20.0	[9]
DOL	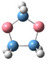	78	−95	−6	6.79	0.58	1.22	18	[9, 49]
DEGDME (G2)	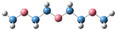	162	−64	57	7.4	1.06	1.97	19.2	[9]
TEGDME (G3)	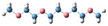	216	−46	111	7.53	3.39	2.24	14	[49]
THF	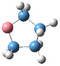	66	−108.5	−14	7.58	0.46	1.63	20.0	[49]
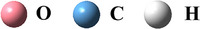									

Organic solvents are the most commonly used in SIBs, while many suffer from poor thermal stability. To enhance battery safety, high‐boiling‐point solvents are generally preferred for formulating non‐flammable electrolytes. Hence, ionic liquids (ILs) and water have been proposed as safer alternatives; however, each presents its own challenges. ILs are more viscous and costly, while the aqueous solution shows a narrow voltage window (1.23 V).^[^ ^54^ ^]^

The low‐temperature performance of solvents is also another important factor to be evaluated in SIBs. Although the desolvation energy of sodium ions is 25–30% lower than lithium ions that enables the SIBs to operate well at low temperatures,^[^ ^10^, ^55^ ^]^ the ionic conductivity, viscosity, and melting point of the solvent under extremely cold condition need careful evaluation.

**Figure 3:**
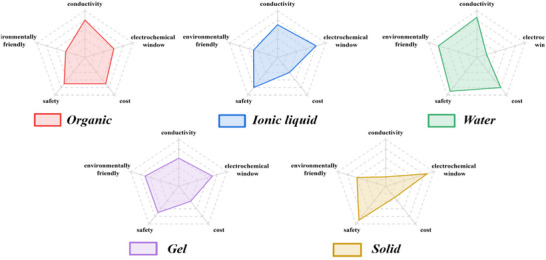
Gains and losses of different electrolytes in SIBs.

To note, when sustainability weighs more and more in battery design, the environmental friendliness has become an important criterion of solvent screening. Taken together, **Figure** **3** compares the gains and losses of various electrolytes. Future electrolyte design is encouraged to optimize these key parameters, ensure electrode compatibility, and integrate multiple factors to improve energy density and cycle stability of SIBs.

### 2.3 Additive

**Figure 4:**
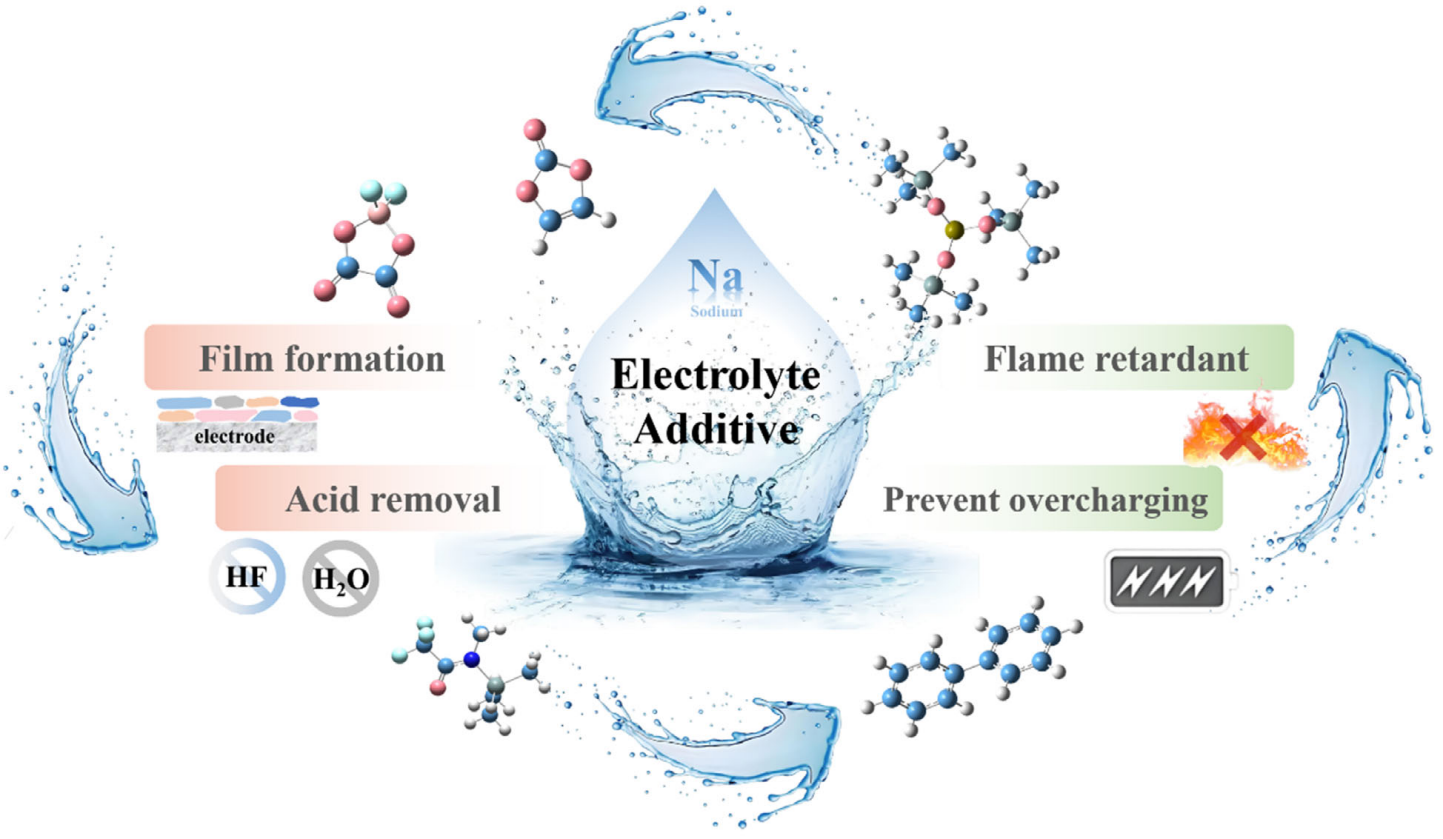
Electrolyte additives with different functions in SIBs.

As mentioned above, the SEI film formed by electrolyte decomposition plays a crucial role in batteries and greatly affects the electrochemical performance. For common electrolytes, the SEI film originates from the decomposition of solvents and anions, which is no longer ideal due to the constantly rising expectation of battery performance. Therefore, additives are common in electrolyte formulations to reconstruct the composition and structure of SEI. Additives are preferred in commercial electrolytes because of the simple operation of addition into the electrolyte at a low mass content, typically less than 10%. This simple cocktail‐type strategy is fully compatible with existing battery production lines. Additives typically act as sacrificial agents and preferentially decompose to participate in film formation, enabling SEI to selectively transport Na^+^, block solvent‐electrode contact, and inhibit subsequent decomposition. It also contributes to stabilizing the structural integrity of SEI under extreme environments (low/high temperatures, high voltage, etc.). In the screening of additives, there is a lack of uniform indicators, relying more on the experience of researchers or theoretical calculations to evaluate molecules involved in SEI formation.^[^ ^56^ ^]^ Classified by their functions, SIB additives mainly include four types, i.e., film‐forming additives, acid‐inhibiting additives, flame‐retardant additives, and anti‐overcharge additives (**Figure** **4**), with details being provided as follows.

#### 2.3.1 Film‐Forming Additives

Film formation is the most common and fundamental function of additives, where a high mechanical strength and stable interfacial layer are formed on the anode and cathode via preferential redox reactions. This layer helps suppress solvent decomposition, facilitates ion transport, and enhances the cycle stability of the battery. The physicochemical properties of some common additives are shown in **Table** **3**.

**Table 3:** Molecular structure and physicochemical properties of common additives.

Additive	Molecular structure	Molar mass [g mol^−1^]	Boiling point [°C]	Melting point [°C]	Density [g cm^−3^]	Refs.
VC (vinylene carbonate)	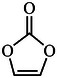	86	162	19	1.36	[57]
FEC (fluoroethylene carbonate)	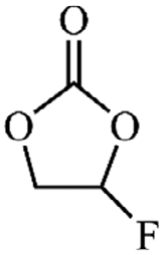	106	249	18	1.45	[58]
ES (ethylene sulfate)	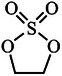	124	231	95	1.60	[59]
1,3‐PS (1,3‐Propanesultone)	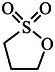	122	180	30	1.39	[59]
TMSP (Tris(trimethylsilyl) phosphate)	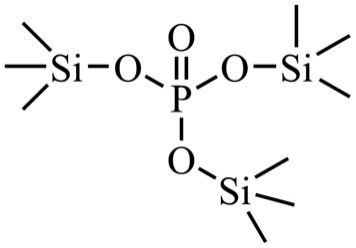	314	228	3‐4	0.945	[60]
FPPN (Ethoxy(pentafluoro)cyclotriphosphazene)	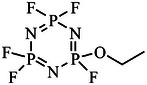	275	42	/	1.36	[61]

**Figure 5:**
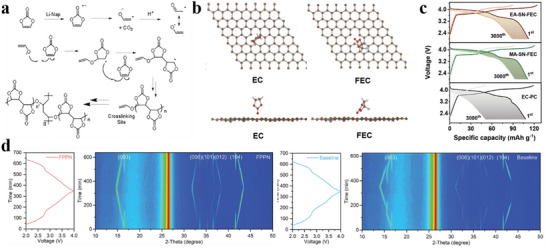
a) VC decomposition mechanism.^[^ ^63^ ^]^ Copyright 2016, the American Chemical Society. b) Adsorption structure of EC and FEC on carbon layer surface.^[^ ^71^ ^]^ Copyright 2024, Royal Society of Chemistry. c) Comparison of cycle performance after introducing SN additive.^[^ ^74^ ^]^ Copyright 2024, Wiley‐VCH. d) The XRD of cathode material in different electrolytes.^[^ ^75^ ^]^ Copyright 2024, Wiley‐VCH.

Due to their wide application scope, carbonate solvents with target functional groups are proposed as additives because of the favorable compatibility between the main solvent and film‐forming capability, such as vinylene carbonate (VC) and vinyl ethylene carbonate (VEC). VC was patented in the 1990s by Sanyo Electric, SAFT, Sony, and Valence Technology Corporation.^[^ ^59^ ^]^ Upon the development of the electrolyte field, VC has gradually become the most typical and widely used additive. The chemical structure of VC is similar to that of common carbonate solvents, e.g., ethylene carbonate (EC) and propylene carbonate (PC). Nevertheless, VC contains unsaturated vinyl bonds and is chemically more active. One of its most important reactions during the electrochemical reaction process is the polymerization of the vinyl bonds to form an interface layer (**Figure** **5**a),^[^ ^62^, ^63^, ^64^ ^]^ effectively ensuring the cycle stability of the electrode. To note, Xu et al.^[^ ^65^ ^]^ used VC in an ether electrolyte, namely 1 m NaPF_6_ in DME. They found that although VC showed good electrochemical performance in the ether electrolyte, VC decomposed in large amounts on the surface of the electrodes into an excessively thick SEI with increased impedance.

Fluoroethylene carbonate (FEC) is another typical carbonate additive that utilizes fluorine's electron‐withdrawing effect to assist the central atom in accepting electrons from the anode and generate a stable SEI film. Some studies found that the improvement of FEC on HC anode varies according to the electrolyte system. When the electrolyte itself forms films poorly, FEC can help grow a stable SEI, whereas when the electrolyte itself has good film‐forming properties, the additive FEC thickens the SEI and increases impedance.^[^ ^66^, ^67^ ^]^ Moreover, FEC can inhibit volume expansion for better cycle performance in Sn, SnO_2_, Sb, and black phosphorus anodes.^[^ ^68^, ^69^, ^70^ ^]^ Recently, Liang et al.^[^ ^71^ ^]^ proposed that FEC in a tilted chemical structure has a lower adsorption energy at a single defect site than EC (Figure 5b). For HC anodes with a high defect structure, FEC can promote uniform SEI formation and inhibit uneven lithium deposition. Although the study was carried out in LIBs, the positive effects for HC anodes can be readily extended to SIBs.

Heteroatom‐containing additives usually build up SEI containing inorganic components based on the characteristics of heteroatoms, mainly including F, N, S, P, B, and Si, for the benefit of the cycle performance of the battery. First, F‐containing substances can raise the antioxidant capacity of the electrolyte and form inorganic substances such as NaF on the electrode surface, which is mechanically strong and can isolate electrons, thus suppressing the growth of Na dendrites.^[^ ^72^, ^73^ ^]^ Moreover, FEC can be used for the protection of the high‐voltage cathode. Pang et al.^[^ ^74^ ^]^ used FEC as an additive to produce NaF‐rich CEI/SEI in an electrolyte system where carboxylic acid esters and succinonitrile are co‐dissolved. As shown in Figure 5c, the introduction of FEC significantly enhanced the cycle stability of the Na_3_V_2_O_2_(PO_4_)_2_F (NVOPF) half‐cell, maintaining performance even after 3000 cycles. The prepared electrolyte achieves high compatibility with high‐voltage NVOPF cathode and HC anode. The full cell was able to cycle at 1C for 500 cycles. NaF‐containing interfaces carry better homogeneity and mechanical stability, encouraging researchers to work on novel F‐containing additives to prolong battery life. For example, Huang et al.^[^ ^75^ ^]^ used pentafluoro(phenoxy)cyclotriphosphazene (FPPN) as a multifunctional electrolyte additive to reconstitute the interfacial layer. After adding FPPN, it was found that due to its weak solvation ability, FPPN hardly participated in the solvation structure. Nevertheless, its introduction could effectively weaken the coordination between Na^+^ and organic solvents (PC, EC, and DEC) and enhance the coordination of Na^+^‐FEC, which enhanced the decomposition of FEC. The formation of interfacial NaF was promoted due to the F‐substituent groups and π–π conjugated ‐P = N‐ structure of FPPN. The capacity retention of a 5 Ah pouch battery containing 2 wt% FPPN was as high as 88.9% after 1000 cycles, demonstrating a superior enhancement of electrochemical performance by FPPN. In parallel, the cycling process of the NaNi*_x_* Fe*_y_* Mn*_z_* O (NNFMO) cathode was characterized by in situ XRD analysis (Figure 5d), and the shifts of all characteristic peaks were lower after that by the addition of FPPN, which proved that its derived CEI could improve the structural stability of NNFMO during the cycle process.

**Figure 6:**
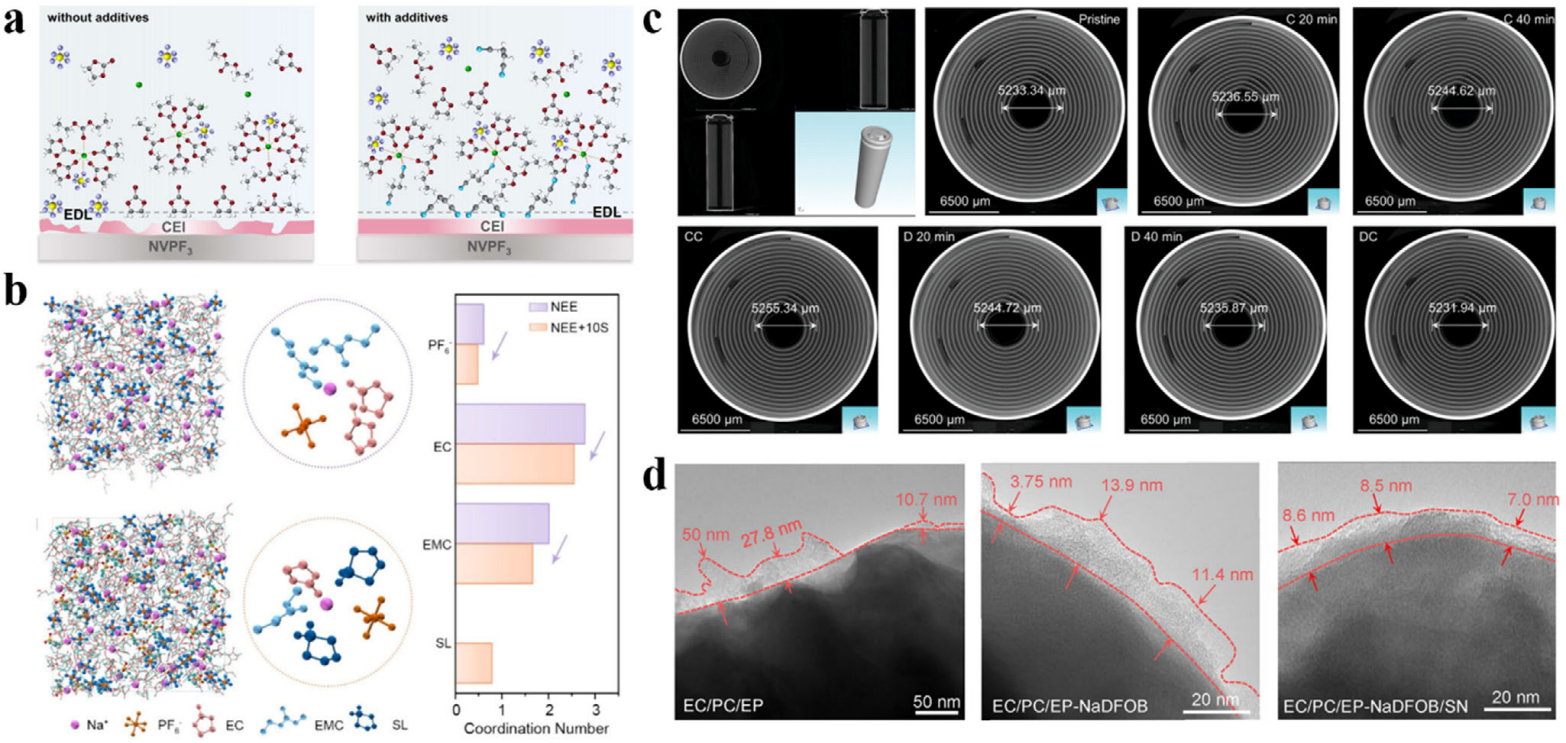
a) Adsorption mechanisms in electrolytes with and without additives.^[^ ^78^ ^]^ Copyright 2024, American Chemical Society. b) The influence of SL additives on solvation structure.^[^ ^79^ ^]^ Copyright 2024, American Chemical Society. c) Internal structure of cylindrical batteries during charging and discharging processes.^[^ ^87^ ^]^ Copyright 2024, Wiley‐VCH. d) Morphology of CEI formed by different electrolytes.^[^ ^88^ ^]^ Copyright 2024, Wiley‐VCH.

For N‐ and S‐containing additives, it is possible to grow highly conductive Na_3_N and Na_2_S for better transport of Na^+^ at the interface. Substances with nitrile (─C≡N) groups are usually used as N‐containing additives, such as succinonitrile^[^ ^76^ ^]^ and 1,3,5‐pentanetricarbonitrile (PTN),^[^ ^77^ ^]^ which operate well on high‐voltage cathodes and enhance the high‐voltage capability of electrolytes. Li et al.^[^ ^78^ ^]^ designed a succinonitrile (SN) additive bearing two ─C≡N groups and elaborated a high‐voltage stabilization mechanism. As stated in their report, SN can replace part of the organic solvent in the solvation structure, where the ─C≡N group at one end coordinates with Na^+^, and the other ─C≡N repelled into the electrical double layer (EDL) (**Figure** **6**a), which is in turn preferentially adsorbed and oxidized into a stable and electrically insulating interface rich in CN^−^/NCO^−^/Na_3_N. With this strategy, the frequency of contact between solvent molecules and the electrode surface is reduced, so the solvent‐induced side reactions are minimized, and a robust CEI can be constructed to enable the Na_3_V_2_(PO_4_)_2_F_3_ (NVPF) cell with excellent stability and high multiplicity capability at 4.3 V. S‐containing additives, e.g., 1,3‐propane sultone (PS), 1,3,2‐dioxathiolane‐2,2‐dioxide (DTD) and ethylene sulfite (ES), usually construct an interface being fast ionic conductors through the decomposition of SO_2_/SO_3_/SO_4_ groups. Recently, Chou et al.^[^ ^79^ ^]^ found a sulfolane (SL) with a S═O double bond as a S‐containing additive not only creates a S‐containing inorganic‐rich interface, but also impacts its solvation structure. As shown in Figure 6b, the structure of the solvated sheath was calculated by molecular dynamics (MD), and the introduced SL could enter the solvated sheath and weaken the coordination of Na^+^ to the solvent, thus accelerating the desolvation process. The dual modification of the solvated structure and interface resulted in a capacity retention of 78.3% over 500 cycles for the pouch battery with Prussian blue (PB) cathode and HC anode. These discussions above indicate that the SEI‐forming mechanism of additives is strongly dependent on target functional groups in their chemical structures, and that the additives tend to affect the solvated structure of SEIs. In this context, when designing additive molecules, it is necessary to consider both the interface and solvation environment to synergistically advance the cycle stability and ion transport kinetics of the battery.

Apart from F and S, the P‐containing additives are often used for the enhancement of flame‐retardancy, because they can capture and eliminate oxygen radicals and terminate chain reactions, thereby enhancing the non‐flammability of organic electrolytes.^[^ ^80^, ^81^ ^]^ They will be discussed in detail in later sections.

An additive containing multiple heteroatoms often achieves more ideal electrochemical performance. For example, researchers used the anion acceptor tris(pentafluorophenyl)borane (TPFPB) as an electrolyte additive,^[^ ^82^ ^]^ which contains both F and B heteroatoms. The B as the core atom is electron‐deficient, and interacts with the anion to weaken its solvation effect. However, the coordination of Na^+^ with EC increases. Unlike other reports, this work reduces the proportion of free solvent in the electrolyte, thereby improving high‐voltage oxidation stability. At the same time, the F makes the CEI layer rich in NaF, so as to enable SIBs to be stably cycled for 100 times at 4.2 V and 60 °C at a capacity retention rate of 86.9%. It can be seen that when designing additive molecules, the enrichment of various functional groups can multiply the efforts and synergistically advance the performance of SIBs. Of course, the simultaneous addition of several additives can also play multiple roles at the same time. For example, Wu et al.^[^ ^83^ ^]^ optimized the interface by combining complementary additives of FEC and tris(trimethylsilyl) phosphite (TMSPi); due to the compensation for the high interfacial impedance by TMSPi and the decomposition by‐products brought by FEC, the assembled NVPF||HC cell operated in a wide temperature range of −25 to75 °C. Recently, Wang et al.^[^ ^84^ ^]^ sought to determine which component of SEI is most conducive to ion transport. Through extensive calculations, they found that Na^+^ has the lowest diffusion energy barrier in Na_2_SO_4_. On top of that, the effect of SEI components on the electrolyte was also considered. It was found that Na_3_PO_4_ had the weakest adsorption of solvents but strong adsorption of anions, which would prevent solvents from entering the electric double layer (EDL), and anions would preferentially adsorb on the HC anode surface to form an inorganic‐rich interface. To achieve the Na_2_SO_4_ and Na_3_PO_4_ components in the SEI, they introduced triphenyl phosphate (TPP) and diethyl sulphate (DTD) additives to achieve such a P‐S interface. This work provides us with a new perspective: namely, the impact of additives on the electrolyte components should be considered, which imposes higher requirements on the components of the SEI.

In the previous sections, it has been mentioned that commonly employed sodium salts as additives mainly include NaDFOB, sodium difluorophosphate (NaDFP), NaBF_4_, NaOTF, etc., in addition to a small amount of NaTFSI and NaFSI. These sodium salts have good film‐forming performance. Similar to LIB, the film‐forming ability of inorganic salts depends on the anion, whereby a typical example is NaDFOB of high thermal stability and good solubility.^[^ ^85^, ^86^ ^]^ Zhang et al.^[^ ^87^ ^]^ introduced NaDFOB into an ether‐based electrolyte, and DFOB^−^ preferentially reacted via oxidation and reduction to establish a robust B‐ and F‐rich interface at both the anode and the cathode, inhibiting dendritic growth to enhance cycle stability in FeMn‐based Prussian blue (FeMnHCF)||HC cylindrical batteries. What's more, the inner microstructure of the cylindrical battery was analyzed by non‐destructive X‐ray computed tomography (CT), and the inner condition of the battery during charging and discharging is shown in Figure 6c. No additional substances were produced, and the electrode exhibited reversible expansion and contraction. This indicates that NaDFOB can stabilize the battery and has practical prospects. Additionally, Pang et al.^[^ ^88^ ^]^ introduced NaDFOB and SN as synergistic additives into the electrolyte, and by observing the Na_3_V_2_(PO_4_)_3_ (NVP) cathode interface after cycling, the CEI formed by the dual additives was found to be thinner and more homogeneous, with superior performance to that of the electrolyte with NaDFOB alone (Figure 6d). Such a robust interface suppresses the continuous decomposition of the electrolyte to operate SIBs in a wide temperature range of −45 to 60 °C. With more and more research, NaDFOB is becoming a well‐known additive. In the ongoing research, NaDFOB is well recognized as additive, but not to forget that NaDFOB when added beyond 3 wt.% amplifies the impedance due to the thicker CEI.^[^ ^89^ ^]^

The efficacy of additives is often reflected in their ability to undergo reduction or oxidation reactions on the electrode surface before solvents to form a stable SEI. Besides participation in interface formation, they interact complexly with solvents, and this interaction determines the solvation structure and overall performance of the electrolyte. First, there is intense competition between additives and solvent molecules, manifested by some additives replacing part of the solvent molecules in the solvation shell due to their strong coordination with Na^+^, thereby suppressing solvent coordination and decomposition. Other additives do not participate in the solvation structure, but interact with solvent molecules on a physical (e.g., van der Waals forces) or chemical (e.g., coordination) level. These interactions alter the solvation behavior, for example, by weakening solvent coordination and accelerating desolvation. When considering the EDL, some adsorbent additives repel solvent molecules by occupying the EDL, thereby preventing their decomposition and the formation of unstable SEI. Meanwhile, some additives that constitute SEI components (such as Na_3_PO_4_) can act as solvent repellents as well. Therefore, additives and solvents do not simply coexist, but rather, they interact through competition, repulsion, and interface formation to synergistically construct a stable working environment for SIBs.

#### 2.3.2 Acid‐Scavenging Additives

NaPF_6_ is one of the most common sodium salts in commercial electrolytes, with somewhat noticeable hydrolysis problems, especially at high voltage, generating corrosive by‐product hydrofluoric acid (HF). HF not only corrodes the already formed SEI/CEI, but also accelerates the dissolution of the cathode transition metal.^[^ ^90^, ^91^ ^]^ Therefore, additives to capture HF or H_2_O and thus to avoid the existence of HF in the electrolyte are necessary. For this purpose, additive molecules containing target functional groups are used in SIBs, such as substances containing siloxyl (Si─O) and silicone‐nitrogen (Si─N) groups. Xiang et al.^[^ ^92^ ^]^ used N, O‐bis(trimethylsilyl) trifluoroacetamide (BSTFA) as an additive. Through theoretical calculations, there exist two reaction paths for Si─N/Si─O bond to react with H_2_O, and the Δ*G* values are −0.35 and −5.53 eV, respectively, both of which are lower than that of the reaction energy of NaPF_6_ with water (−0.161 eV). Thus, BSTFA can effectively remove water molecules from the electrolyte. FEC has a good effect in improving the performance of SIBs, but its poor thermal stability is susceptible to acidic substances and produces harmful substances such as HF.^[^ ^83^, ^93^ ^]^ Therefore, using some synergistic additives is a good way to tackle this problem. For example, TMSPi contains a trimethylsilyl structure, which can remove the generated HF and H_2_O through the Si─O bond. On top of that, there are additives containing N═C═O and P(OR)_2_R groups that can play a role in restricting HF generation. Apart from the reaction effect of target functional groups, adsorption function of substances, such as ZSM‐5 nano‐zeolite^[^ ^94^ ^]^ can capture H_2_O or HF molecules, limiting their corrosion of the electrode and SEI. Chen et al.^[^ ^95^ ^]^ recently proposed 4,4′‐(1,4‐phenylenebis(oxy))‐bis(butane‐1‐sulfonate)‐15‐crown‐5 (15PBS) as an additive. First, the 15PBS molecule can effectively adsorb water molecules, transforming them from an aggregated state to an inactive state, thereby inhibiting water decomposition and generation of the acidic substance HF. 15PBS also exhibits strong adsorption energy on the HC surface, enabling it to penetrate the EDL and repel solvent molecules, thereby inhibiting solvent decomposition. Furthermore, MD simulations revealed that the introduction of the additive reduces the coordination number of Na^+^ with the solvent, which facilitates the desolvation process. Therefore, the use of the 15PBS additive provides a stable interface and is not susceptible to multiple side reactions at high temperatures.

In SIBs, the SEI solubility is higher than that in LIBs, worsening the SEI stability problem. Therefore, some HF‐like acids have an amplified impact on the battery cycle performance and lead to the dissolution of transition metals in cathode materials (e.g., layered oxides), especially in high‐temperature environments. Consequently, it is essential to consider acid‐scavenging functional groups when designing additive molecules, which will fulfil the goal of additive multifunctionality.

#### 2.3.3 Flame Retardant Additive

At present, the organic electrolyte routinely used in SIBs is flammable, which makes the battery a potential safety hazard. Some flame‐retardant additives, e.g., P‐ and F‐containing ones, can enhance the safety of the battery. The P‐containing additives inhibit the combustion of the organic electrolyte by capturing the oxygen radicals and blocking the occurrence of the free radical chain reaction.^[^ ^96^, ^97^ ^]^ Common additives are mainly phosphate esters, phosphites, and cyclic phosphonitrile, among which trimethyl phosphate (TMP) and triethyl phosphate (TEP) are also used as solvents to enhance the flame retardant effect. Ma et al.^[^ ^98^ ^]^ compared the effects of ethoxy (pentafluoro) cyclotriphosphazene (PFPN) and hexafluorocyclotriphosphonitrile (HFPN) to the electrolyte. Their study showed that 5 wt% PFPN in the electrolyte had a significant nonflammable effect, and PFPN could remove PF_5_, avoiding the production of acidic substance HF. The cycle performance of PFPN was even better than that of HFPN, because of the formation of inorganic Na_3_N/NaF‐rich SEI on the electrode surface. Such a multifunctional additive is what researchers have been longing for.

Some substances with high fluorine content, due to their high flash point, have flame‐retardant effects and are commonly used as solvents in electrolytes. However, solvents with a high F content tend to be more costly, so some studies take this into account and employ only a small amount for the flame‐retardant effect. For example, methyl nonafluorobutyl ether (MFE) has good electrochemical stability, but its addition leads to low ionic conductivity (0.43 mS cm^−1^).^[^ ^99^ ^]^ For flame‐retardant effects, additives at a lower content are often inferior to flame‐retardant solvents, so if the safety of the battery is to be ensured, the use of non‐flammable solvents without sacrificing other parameters (e.g., ionic conductivity, cycle stability, and electrochemical stability) is necessary to ensure its safe use in practical commercialization.

#### 2.3.4 Anti‐Overcharge Additive

The high voltage of SIBs is generally around 4 V. When overcharging occurs in the battery, the voltage will continue to rise, and the chemical reaction will intensify, so that the temperature will rise and cause safety problems such as combustion and explosion. To solve this problem, anti‐overcharge additives have been proposed. These additives do not participate in the reaction during normal charging and discharging, and their decomposition voltage is generally in the range of 4.3‐5 V, which is not only higher than the charging cut‐off voltage of the battery, but also lower than the decomposition voltage of the electrolyte. When the battery is overcharged, these kinds of additives can react rapidly to form an insulating film that hinders the electronic conduction and prevents the continuation of the overcharging.^[^ ^100^ ^]^ For example, it was reported that biphenyl (BP) undergoes polymerization reaction when the voltage exceeds 4.3 V, creating a protective layer and improving the safety of the battery.^[^ ^101^ ^]^ However, this protection is irreversible and will undoubtedly cause an increase in interfacial impedance. Recently, some companies have proposed 2‐fluorobiphenyl (FBP) as an anti‐overcharging additive by replacing a H with a F on the benzene ring of BP. Its decomposition voltage can be increased to 4.6 V, with weakened activity of the polymerization reaction, and a smaller increase in impedance due to the addition of F. It has a lesser effect on the performance of the battery. To sum up, the introduction of ideal polymeric anti‐overcharge additives should impose little‐to‐no effect on battery performance, and this is expected to be the next focus of researchers.

Another group of anti‐overcharging additives is the shuttle additives. They oxidize at cathode first and then shuttle to anode to be reduced, preventing overcharging by shuttling back and forth between cathode and anode, and reducing excessive current and stabilizing voltage.^[^ ^102^ ^]^ Qu et al.^[^ ^103^ ^]^ developed a functional organic salt, trisaminocyclopropenium perchlorate (TAC ClO_4_), and used it as a redox shuttle for overcharge protection in SIB systems. It was able to diffuse rapidly in both redox states, and the addition of 0.1 m TAC‐ClO_4_ enabled the NVP cathode to withstand 400% overcharge; it was demonstrated that this overcharge‐protection salt had no significant effect on the electrochemical performance of the NVP||HC full cell.

The choice of different additives depends on the composition of the electrolyte. It is necessary to clarify the function of the additives and design the molecular structure rationally. At present, most of the additive designs for SIBs are borrowed from LIB. In order to pick up additives suitable for SIBs, not only the researchers’ scientific intuition is needed, but also the screening enabled by machine learning and other artificial intelligence tools is needed to avoid the traditional trial‐and‐error process, thereby accelerating additive discovery for SIB electrolyte.

## 3 Different Electrolyte Systems

Current development of SIB liquid electrolytes is guided by the LIB electrolyte, which consists of organic electrolyte, aqueous electrolyte, ionic liquid electrolyte, and gel electrolyte. Along with the expansion of the field, more electrolytes and new concepts are demanded, which ask for a better understanding of this important transport medium, including solvation structure, ion transport process, and film formation behavior. This section will review various SIB liquid electrolyte systems and discuss their roles in SEI formation.

### 3.1 Organic Electrolyte

#### 3.1.1 Ester Electrolyte

**Figure 7:**
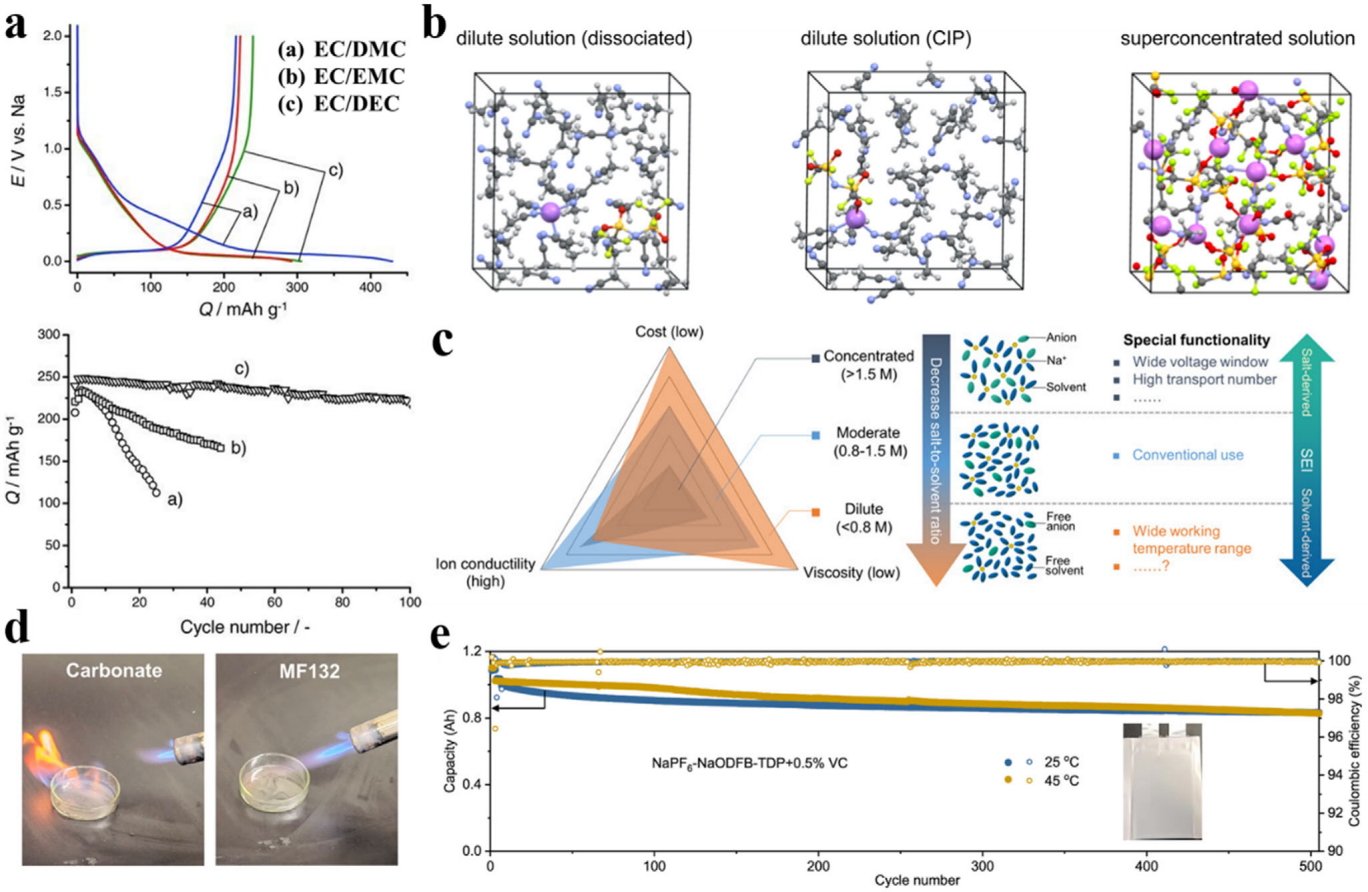
a) First cycle and long cycle performance of HC anodes using different electrolytes.^[^ ^109^ ^]^ Copyright 2011, Wiley‐VCH. b) The interaction between ions and solvents in electrolytes of different concentrations.^[^ ^118^ ^]^ Copyright 2014, American Chemical Society. c) Comparison of properties of electrolytes with different concentrations.^[^ ^113^ ^]^ Copyright 2020, American Chemical Society. d) Ignition tests for the conventional carbonate electrolyte and the MF132 electrolyte.^[^ ^116^ ^]^ Copyright 2024, American Chemical Society. e) Long cycle performance of Ah‐level NFM||HC pouch cell using TDP electrolyte.^[^ ^117^ ^]^ Copyright 2024, American Chemical Society.

As early as the 1990s, electrolyte formulations in advanced lithium battery, e.g., 1 m LiPF_6_ dissolved in a solvent mixture of EC and linear carbonate were established. Such formulations can shape a SEI film at the anode, preventing the electrolyte from further decomposing.^[^ ^104^, ^105^ ^]^ However, the melting point of EC is relatively high (37 °C), while structurally similar PC has a lower melting point (−48.8 °C) with a high solvating power to dissolve salts. The role of PC as a capable electrolyte solvent is somehow limited by its strong binding power with lithium ions, as it causes the solvent to be co‐embedded in the exfoliation of the graphite layer, and eventually the collapse of the graphite structure.^[^ ^106^, ^107^ ^]^ As for SIBs, graphite is not suitable due to thermodynamically unfavorable Na^+^‐graphite intercalation.^[^ ^108^ ^]^ HC anode is widely used in SIB anode materials due to its large layer spacing and good sodium storage capacity. The layered structure of HC will not be exfoliated by the co‐intercalation behavior of PC, so in principal PC can be used in SIB electrolytes. However, PC solvent has poor film‐forming properties. When PC is used as a single solvent, the cycle performance is not ideal. On the contrary, EC has excellent film‐forming properties and can create a stable SEI. Although EC may experience significant viscosity issue or even solidification at low temperatures, it has a high dielectric constant and good antioxidant properties. Currently, EC remains the main solvent used in SIBs. Usually, EC is combined with some low‐melting point co‐solvents to adapt a proper viscosity. Common co‐solvents are linear carbonates such as dimethyl carbonate (DMC), ethyl methyl carbonate (EMC), and diethyl ethyl carbonate (DEC). Komaba et al.^[^ ^109^ ^]^ investigated a series of carbonates as solvents, and by comparing the effect of different electrolytes on the HC anode, it was found that the dual‐solvent electrolyte system had better cycle performance than a single solvent one (EC or PC). Moreover, the performance of EC mixed with DEC was superior to other co‐solvents, with 100 stable cycles and an initial coulombic efficiency (ICE) of 78% (**Figure** **7**a). It has also been investigated to mix EC and PC and found that its thermal stability is better than the mixture of EC and other linear carbonate. The electrolyte formulated by dissolving NaPF_6_ in EC/PC (1:1 v/v) showed the best performance in HC half cells.^[^ ^40^ ^]^

The usual concentration of salt is 1 m in the electrolyte, as research has found that electrolytes have the highest ionic conductivity around 1 m.^[^ ^110^ ^]^ Therefore, the concentration of commercial electrolytes is 1–1.2 m. However, a series of studies on electrolyte concentration has provided new inspirations for the design of electrolytes. Highly concentrated electrolytes (HCEs) were first used in LIBs. HCEs typically refer to salt concentrations greater than 2 m. Increasing the salt concentration results in enhanced coordination between cations and anions and a decrease in the amount of free state solvent molecules, resulting in a new electrolyte environment with a special solvated structure.^[^ ^111^ ^]^ The interaction of the solvent and the ions is shown in Figure 7b. In electrolytes at regular concentrations, solvents and cations form solvated structures of solvent‐separated ion pairs (SSIPs). As the salt concentration increases to a high concentration (>2 m), solvated structures of contact ion pairs (CIPs) and aggregates (AGG) with more anionic coordination will come into being.^[^ ^112^ ^]^ Not only that, due to the smaller Stokes radius of Na^+^, SIB has a superior kinetic rate compared to LIB, which allows the SIB electrolyte to achieve sufficient ionic conductivity at low concentrations. Therefore, efforts have been made to reduce the salt content to minimize cost and operating temperature. For example, Hu et al.^[^ ^113^ ^]^ proposed the use of an ultra‐low concentration electrolyte of 0.3 M NaPF_6_ in EC/PC (1:1 vol/vol). Compared to the conventional concentration electrolyte, the low concentration electrolyte delivered lower viscosity and cost, although the ionic conductivity was lower (**Figure** **7**c). In addition, the hydrolysis of NaPF_6_ in a low‐concentration electrolyte is suppressed, thus the generation of hazardous substances such as HF is greatly reduced, and the operating temperature range is extended to −30 to 50 °C. The proposal of low‐concentration electrolytes provides a new approach for SIBs to reduce costs, and is expected to be extended to other electrolyte systems.

Beyond the common carbonates, researchers have explored the potential of other esters as SIB solvents. Some of these carboxylic acid esters have been investigated due to its low viscosity and low melting point. Similar to PC, these carboxylic acid esters have poor film‐forming properties and are usually mixed with some film‐forming agents for use. For example, researchers have successfully prepared a low‐cost ester electrolyte using ethyl acetate (EA) as a single solvent and FEC as a film‐forming additive.^[^ ^114^ ^]^ Due to the weak binding energy of EA with Na^+^, the electrolyte exhibits a weakly solvated coordination structure, which promotes the possibility of fast charging of SIBs. A pouch battery with Na_0.97_Ca_0.03_[Mn_0.39_Fe_0.31_Ni_0.22_Zn_0.08_]O_2_ (NCMFNZO) as cathode and HC as anode has a capacity retention rate of 80% using this electrolyte for 250 cycles. Similar solvents include ethyl acetate (EA),^[^ ^74^ ^]^ methyl acetate (MA),^[^ ^88^ ^]^ etc. These studies indicate that linear esters have good application prospects in low‐temperature SIBs. Care should be taken to match them with suitable film‐forming agents to ensure the formation of stable interfaces and to prevent further decomposition of the linear esters.

It has been mentioned in the previous sections that the introduction of some phosphate esters can bring a flame‐retardant effect to the electrolyte. In order to achieve full non‐flammability, it is not enough to add it as an additive, so phosphate esters were studied as solvents to develop high‐safety flame‐retardant SIB electrolytes. Researchers dissolved 0.8 M NaPF_6_ in a trimethyl phosphate (TMP) solvent with 10% FEC additive to successfully achieve a non‐flammable electrolyte.^[^ ^115^ ^]^ Next Cao et al.^[^ ^116^ ^]^ introduced tris(2,2,2‐trifluoroethyl) phosphate (TFEP) to regulate anion‐cation interactions in TMP‐based electrolytes, enabling the design of a low‐salt, highly compatible phosphate ester electrolyte. At a molar ratio of TMP:TFEP 3:2 (MF132), the ICE of HC anode reached 82%, higher than that of ordinary carbonate electrolyte, and the electrolyte was nonflammable (Figure 7d). Additionally, the NaF‐rich inorganic SEI obtained from the reductive decomposition of TFEP has good interfacial compatibility, which ensures the stability of the electrolyte and achieves the long cycle life of the SIBs in a wide temperature range (−20 to 60 °C). In the practical application of SIBs, the electrolyte is often mixed with binary or ternary to enhance the comprehensive performance of the battery. This is because a single solvent is difficult to cover all aspects of battery requirements, so couplings of multiple solvents are used to achieve the goal. Pan et al.^[^ ^117^ ^]^ proposed a high‐performance electrolyte that enhances the chemical and electrochemical stability by carefully adjusting the ternary solvent‐solvent interactions. Mixing three solvents, triethyl phosphate (TEP), DMC, and trifluorotoluene (PhCF_3_), to set up an electrolyte (the electrolyte is named TDP) to effectively suppress the structural phase transition of layered oxide cathodes and the dissolution of transition metals compared to traditional carbonate electrolytes. Moreover, the electrolyte itself is non‐flammable due to the presence of phosphate ester, and the ternary solvent mixture with diverse solvation properties provides a new perspective to regulate the local solvation structure and improve the Na^+^ transport kinetics. The use of this ternary electrolyte with additives enabled the Ah‐level NaNi_1/3_Fe_1/3_Mn_1/3_O_2_ ||HC pouch battery to be cycled stably for 500 cycles at both 25 and 45 °C (Figure 7e).

In short, ester electrolyte is commonly used in SIBs. Conventional carbonate solvents alone can no longer meet the demand, so some studies mix phosphate, carboxylic acid ester, or other solvents with the carbonate solvent to achieve the effect of flame retardant, fast charging, or low‐temperature operation. On top of this, studies on fluorinating carbonates to reduce their viscosity and achieve a NaF‐rich interface were carried out. Regardless of the approach, it has long been demanded that the electrolyte has a good film‐forming component and a low‐viscosity solvent to ensure long‐cycle life and fast ion transport. Although some studies currently propose “EC‐free” electrolytes, EC, with its good ability to dissolve salts and its high thermal stability, remains the main component of commercial SIB electrolytes. The design of commercial electrolytes is different from that of cutting‐edge scientific research, as it requires consideration of many practical factors important in the markets, so carbonate electrolytes will stay as the mainstream in the near future. How to introduce alternatives to improve the performance of traditional carbonate electrolytes may be a more worthwhile topic from a commercial perspective.

#### 3.1.2 Ether electrolyte

**Figure 8:**
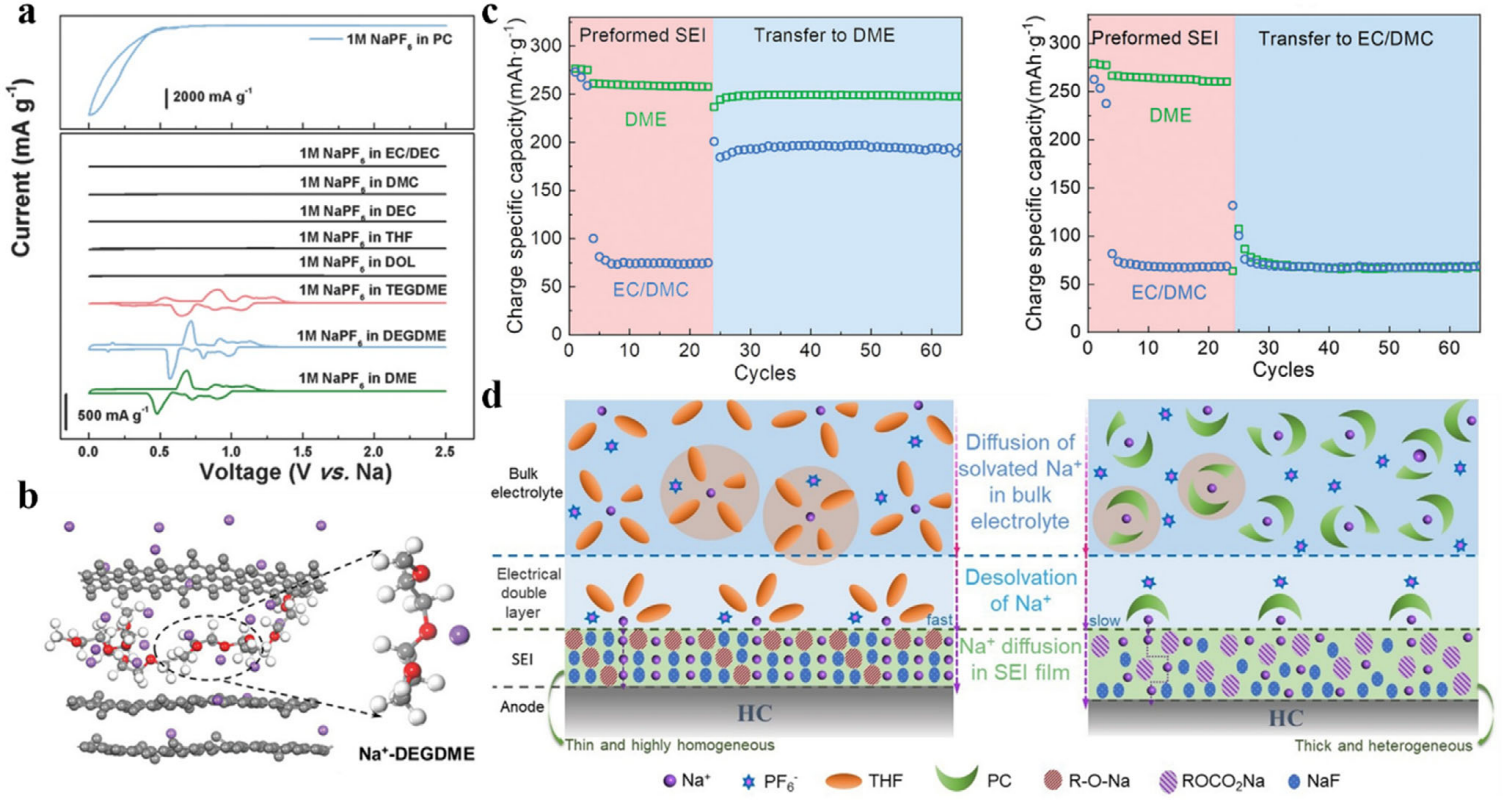
a) CV curves of different Na‐solvent systems.^[^ ^120^ ^]^ Copyright 2016, Wiley‐VCH. b) Computationally simulated structure of Na^+^‐solvent co‐insertion into carbon layer.^[^ ^124^ ^]^ Copyright 2021, Wiley‐VCH. c) Pre‐formed SEI exchange electrolyte experiment.^[^ ^125^ ^]^ Copyright 2022, Wiley‐VCH. d) SEI and sodium ion desolvation process formed by different electrolytes.^[^ ^126^ ^]^ Copyright 2022, Wiley‐VCH.

Similar to esters, ether electrolytes are used as well in SIBs due to low viscosity and high ion‐conductivity. It is noteworthy that ether electrolytes are discarded by LIBs due to their decomposition at high voltage (>4 V). In SIBs, the voltage is lower than that of LIB, so that ether electrolytes are reconsidered. It has been found that graphite can form unique intercalation compounds in ether electrolytes, enabling graphite anodes to be used in SIBs. As early as 2014, Jache et al.^[^ ^119^ ^]^ found that diethylene glycol dimethyl ether (DEGDME)‐based electrolytes can complex with Na^+^ co‐embedded in graphite layers to form sodium‐solvent‐graphite compounds. Subsequently, Kang et al.^[^ ^120^ ^]^ conducted experiments on a variety of solvents to investigate the conditions under which graphite co‐embedded layers occur, including linear ethers/esters and cyclic ethers/esters. The cyclic voltammetry (CV) test results (**Figure** **8**a) found that the co‐intercalation behavior occurred only for linear ethers (ethylene glycol dimethyl ether (DME), diethylene glycol dimethyl ether (DEGDME), and tetraethylene glycol dimethyl ether (TEGDME). As for the PC electrolyte, the CV curve has only a reduction peak without an oxidation peak, which indicates the presence of irreversible embedding and side reactions. These linear ethers will build up chelated structures with Na^+^ and therefore have stronger binding energies and higher desolvation barriers. For reversible Na^+^‐solvent co‐intercalation, the LUMO level of the complex should be larger than the Fermi level of graphite to ensure the stability during the reduction process. These findings allow graphite anodes to be used with linear ether‐based electrolytes in SIBs. From the practical application point of view, there are still non‐negligible problems, e.g., limited capacity and large volume expansion,^[^ ^121^, ^122^, ^123^ ^]^ limiting the use of, graphite anodes in SIBs. HC anodes as an alternative possess a more complex structure and Na^+^ storage mechanism, and still have a carbon layer composed of graphite domains. Because of the larger interlayer spacing of HC, researchers started to investigate whether ether electrolytes have co‐insertion behavior in HC anodes.

First, HC anodes in ether‐based electrolytes were found to exhibit higher ICE and better rate performance than in ester‐based electrolytes. The mechanism remains a myth to date. Wu et al.^[^ ^124^ ^]^ used DEGDME versus EC/DEC to compare the differences in kinetics between ether‐based and ester‐based electrolytes in HC‐based cells. Experimental and theoretical calculations confirmed the existence of co‐intercalation of ether‐based electrolytes in HC anode, as shown in Figure 8b, where Na^+^ and solvent diffuses into the carbon layers. Then, the structure of SEI formed by different systems of electrolytes was investigated in depth. It was found that the fast Na^+^ storage kinetics of HC in ether‐based electrolytes can be attributed to the following three aspects: (1) higher migration rates of solvated Na^+^ in ether‐based electrolytes than ester‐based ones; (2) ether‐based electrolytes avoid the slow desolvation process through the co‐insertion behavior; (3) The SEI formed by the decomposition of ether‐based electrolytes contain more inorganic components and is thinner, resulting in lower impedance of the SEI film and charge transfer impedance at the interface. A different view was also presented in a recent study. Yan et al.^[^ ^125^ ^]^ similarly compared the variability in kinetics of ester‐ and ether‐based electrolytes in HC, with the difference that the ether solvent used was DME. In this work, the co‐intercalation behavior of the ether electrolyte was not found, and it is believed that the desolvation process of the two electrolytes and the differences in SEI film properties have a significant impact on the migration kinetics of Na^+^. Moreover, the researchers designed an experiment to differentiate the effects of SEI and desolvation behavior on the rate performance. As displayed in Figure 8 **c**, SEI was pre‐formed in different electrolytes and transferred to another electrolyte. The results support that the desolvation process is the major factor causing the difference in kinetic performance between the two electrolytes. Since the binding energy of DME for Na^+^ is not as strong as that of DEGDME, it is possible that the difference in solvents is the true reason why no co‐intercalation behavior was found. However, regardless of the solvent, ether electrolytes have superior kinetic rates to ester ones for HC anodes. There seems to be a consensus among researchers that ether electrolytes construct a thinner, more inorganic SEI, which is essential for facilitating Na^+^ transport.

**Figure 9:**
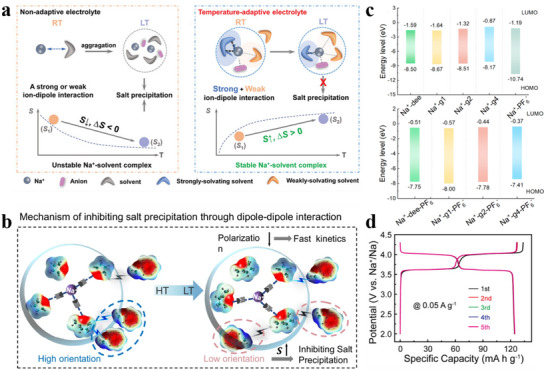
a) The solvation structure variations with temperature driven by entropy change.^[^ ^127^ ^]^ Copyright 2023, Wiley‐VCH. b) Schematic diagram of solvation structure changes at high and low temperatures.^[^ ^128^ ^]^ Copyright 2024, Springer Nature. c) Comparison of HOMO energy levels between Na^+^‐solvent and Na^+^‐solvent‐PF_6_^−^.^[^ ^129^ ^]^ Copyright 2024, Elsevier. d) Charge–discharge curves of NVPF cathodes using prepared electrolytes.^[^ ^130^ ^]^ Copyright 2021, Wiley‐VCH.

Besides linear ethers, cyclic ethers, e.g., tetrahydrofuran (THF), 2‐methyltetrahydrofuran (MeTHF), and 1,3‐dioxolane (DOL), are also employed in SIBs. Compared with linear ethers, cyclic ethers assemble more rigid complexes with Na^+^, which are unfavorable for co‐embedding, and have a lower barrier to desolvation. In combination with the low melting point of cyclic ethers, a large number of studies has been done in such solvents to develop low‐temperature weakly solvation electrolytes. Wang et al.^[^ ^126^ ^]^ found a novel electrolyte by dissolving 1 m NaPF_6_ in THF, which has a faster desolvation process and an optimized electrolyte interface (Figure 8d), allowing commercial HC to reach a high‐rate performance of 5 A g^−1^ in THF‐based electrolyte and a low‐temperature performance of −20 °C. Moreover, Lu et al.^[^ ^127^ ^]^ prepared a low‐temperature SIB electrolyte by mixing THF with DEGDME to avoid the precipitation of sodium salt at low temperature by adjusting the solvation entropy. This electrolyte with a mixture of strongly and weakly solvated solvents showed a solvation structure that varied adaptively with temperature (**Figure** **9**a). This property granted the capacity retention of SIBs at 90.6% after 400 cycles at −40 °C. This work reveals the importance of hybrid entropy gain, which may give better results when multiple solvents are coupled, providing a rational viewpoint for the design of low‐temperature SIB electrolytes. Lately, the same group has continued to explore new electrolytes with “temperature‐adaptive” properties.^[^ ^128^ ^]^ By dissolving NaPF_6_ in MeTHF, THF, and anisole (AN), an electrolyte has been prepared with great temperature‐adaptive properties. By modulating the interactions between the solvents, not only low working temperatures but also good thermal stability at high temperatures are achieved. Briefly, as shown in Figure 9b, at high temperatures, AN interacts strongly with MeTHF to inhibit parasitic reactions, while at low temperatures, AN displays strong interaction with THF to suppress salt precipitation. This adaptation property enables the service temperature of electrolyte in a wide range of −60 to 55 °C.

It can be seen that ether solvents exhibit excellent performance at low temperatures. A key factor limiting their application is the stability at high voltage. Although in SIBs, ≈4 V is at the critical voltage for ether decomposition, higher voltages are unavoidable in order to improve energy density. It is usually believed that the decomposition potential of solvents limits the stability of ether electrolytes under high voltages, but recent work has pointed to the fact that the solvation structure is also critical for the oxidative stability of ether electrolytes. Yan et al.^[^ ^129^ ^]^ chose four solvents of 1,2‐diethoxyethane (DEE), DME, DEGDME, and TEGDME, and used the same sodium salts to prepare four electrolytes with different solvated structures. Based on theoretical calculations, the four solvents were found to have similar HOMO energy levels, their coordination strengths with Na^+^ dominated their differences in oxidative stability. Ether solvents with weak coordination ability (DEE and TEGDME) introduce anions into the primary solvated sheath layer, which lifts the HOMO level up (Figure 9c) and suppresses the onset oxidation potential, making both electrolytes unsuitable on the high‐voltage NVPF cathodes (4.3 V). In contrast, the strongly solvent‐coordinated DME and DEGDME electrolytes build up low‐impedance CEI, which keeps stable on the high‐voltage cathodes. It is noteworthy that the development of high‐voltage ether‐based electrolytes has become the focus of research recently. Through the concept of high‐concentration electrolyte and localized high‐concentration electrolyte, a large number of cations can be coordinated with the ether‐based electrolyte, avoiding the presence of free ether solvents, which makes them less susceptible to oxidation. For example, Wu et al.^[^ ^130^ ^]^ reported a highly coordinated high‐voltage ether electrolyte with a rational design of the solvation structure for SIBs. By adding DOL to dissolve 3.04 M NaPF_6_ in DEGDME, the solvent of the designed electrolyte was highly coordinated, and the free components were reduced. On top of that, the use of this electrolyte formed an inorganic‐rich and robust interface, enabling stable cycling of the high‐voltage NVPF cathode (Figure 9d).

Unlike the LIBs in which high‐voltage ether electrolyte research is extensive, the high‐voltage ether research in SIBs is relatively little at present. This direction in SIBs is in our view very necessary, as high‐voltage NVPF and high‐voltage layered oxides can improve the energy density of the SIBs. If we can successfully match these materials, ether electrolyte will become a very advantageous choice. Current work on ether electrolytes for SIBs focuses on designing weakly solvated structures to promote desolvation and accelerate the kinetic rate, while this undoubtedly ignores the behavior of co‐intercalation in HC anodes. As HC being the most common anode material for SIBs, ether electrolytes can avoid the slow desolvation process by co‐intercalation, and theoretically have faster ion transport. If this mechanism can be rationally applied, more suitable ether electrolytes for SIBs will come to use.

#### 3.1.3 Other Organic Electrolytes

There are some miscellaneous solvents such as nitrile, sulfone, and sulfoxide for SIBs. With the deepening of our understanding, these solvents become growingly attractive to SIB electrolytes. Zheng et al.^[^ ^131^ ^]^ proposed a multiple functional bond integration strategy from the design of electrolyte solvation structure by adding SN, FEC, and NaPF_6_ into PC‐based electrolyte to regulate the interfacial composition and reconstructed the solvation structure. A CEI with multiple functional bond integration consisting of NaN_x_O_y_, NaF and NaP_x_O_y_ was formed on the cathode surface. As revealed, this electrolyte has high oxidative stability, and enables the NFM||HC pouch battery to cycle stably for 1000 cycles. Furthermore, Jinhan et al.^[^ ^132^ ^]^ reconfigured the first and second solvated sheath layers by introducing two solvents with different solvation capacities, EMC and ethoxy (pentafluoro) cyclotriphosphazene (PFPN), into the PC‐based electrolyte. Due to its stronger coordination ability, EMC is able to enter the first solvated layer to compete with PC for Na^+^, while PFPN interacts with other solvents in the outer layer, thus weakening the inner solvation energy of Na^+^. The desolvation barrier is effectively reduced by such a coordinated solvation structure, and PFPN can also participate in the formation of the interface to improve the cycle stability of the battery. It provides superior electrochemical performance over a wide temperature range when applied to Ah‐level NFP||HC pouch cells.

Sulfones usually contain sulfonyl groups, of which cyclobutyl sulfone (SL) is a representative to be added to electrolyte as a solvent or additive. Chou et al.^[^ ^79^ ^]^ found through theoretical calculations that SL exhibits higher HOMO levels and lower LUMO levels than commonly used ester‐based solvents, and is able to preferentially decompose at the electrode surface to form S‐containing interfaces. Classical molecular dynamics (MD) revealed changes in the coordination environment. By adding SL, SL enters the inner solvation layer, weakening the coordination of anions and solvents with Na^+^. An electrolyte containing 10 wt% SL can make Prussian blue (PB)||HC full cells exhibit stable cycling performance.

At present, the application of other components in SIB electrolytes is still limited, and these components, other than ether and ester electrolytes, usually have additional functions, such as high working voltage, and acid removal. Therefore, many studies use them as additives. If they are used as solvents, factors such as the overall ionic conductivity and working temperature of the electrolyte need to be considered as limiting factors for application.

### 3.2 Ionic Liquids

**Figure 10:**
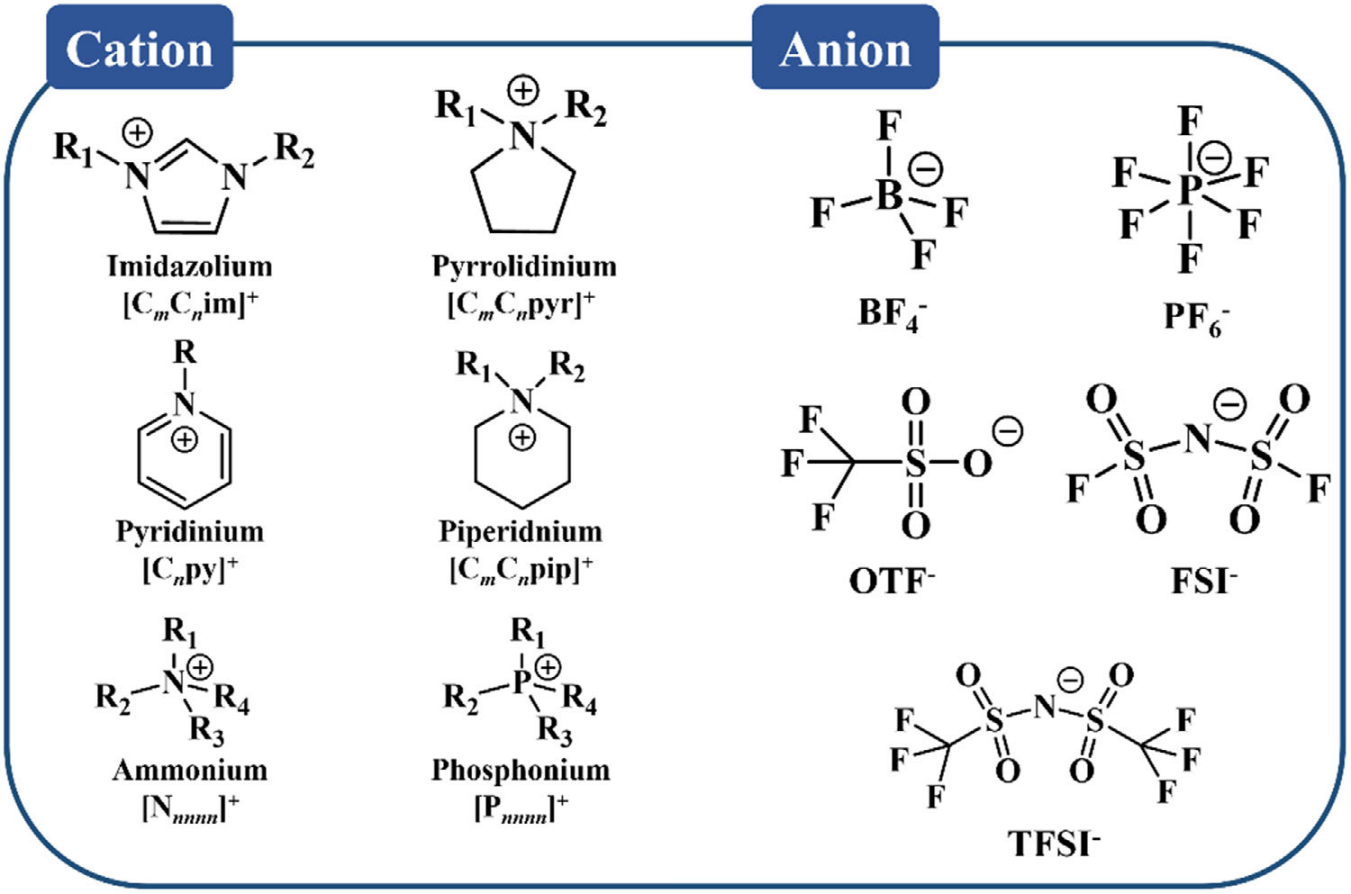
Cations and anions of common ILs in electrolytes.

Ionic liquids (ILs) are organic molten salts that remain liquid at temperatures below 100 °C. They are composed of cations and anions, and their physical and chemical properties, such as polarity and donor ability, can be finely tuned. To date, millions of different ILs have been predicted or synthesized.^[^ ^133^, ^134^, ^135^, ^136^ ^]^ The first IL debatably dates back to 1914, when Paul Walden reported the discovery of ethylamine nitrate,^[^ ^137^ ^]^ which has a melting point of 12–14 °C. However, its discovery did not attract much interest at that time because it is very unstable in air. It was not until late 1990s that ILs began to be used as an electrolyte in rechargeable batteries. It was found that ILs had a good application prospect as an electrolyte, with a wide electrochemical window and thermal stability.^[^ ^138^ ^]^ However, the high viscosity and high melting point of some ILs, as well as high cost issues limit the practical use of ILs.^[^ ^134^, ^139^ ^]^ Although there are many types of ILs, the ones that can be used as electrolyte components are very limited. Here we summarize the cations and anions of ILs commonly found in electrolytes, as shown in **Figure** **10**. The cations mainly include imidazolium, pyrrolidinium, ammonium and phosphonium, and the anions include mainly BF_4_^−^, PF_6_^−^, OTF^−^, FSI^−^, and TFSI^−^. Different combinations of them result in different properties of ILs.

**Figure 11:**
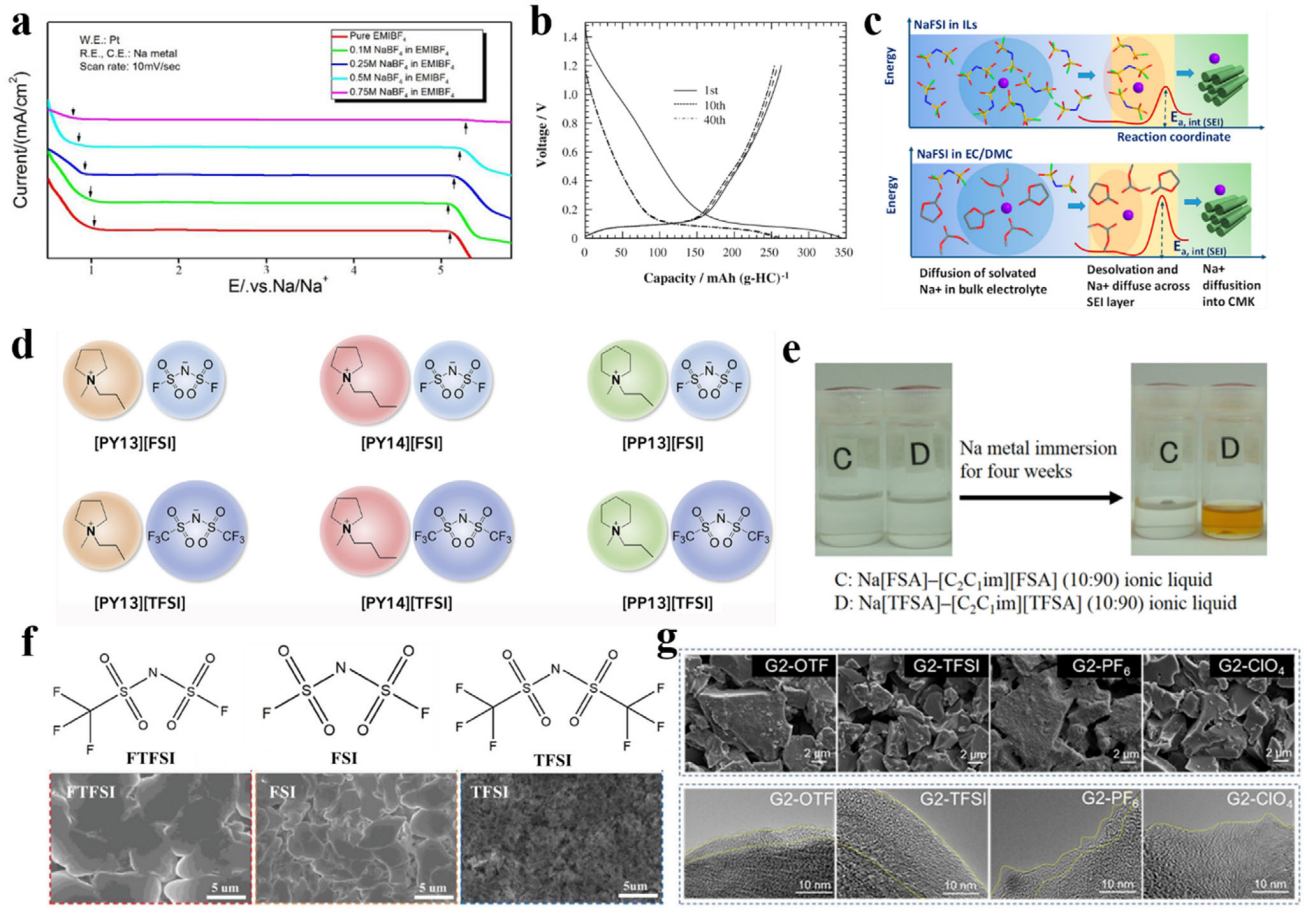
a) The LSV curves of different concentrations of NaBF_4_ in EMIBF_4_.^[^ ^141^ ^]^ Copyright 2016, American Chemical Society. b) Charge discharge curves of HC half‐cell using IL electrolyte at 363 K.^[^ ^143^ ^]^ Copyright 2014, Elsevier. c) Schematic diagram of SEI formed using different electrolytes.^[^ ^145^ ^]^ Copyright 2021, American Chemical Society. d) Six types of IL studied in this work.^[^ ^146^ ^]^ Copyright 2019, Elsevier. e) Optical photographs of sodium metal after four weeks of immersion in different electrolytes.^[^ ^147^ ^]^ Copyright 2016, Elsevier. f) Structure of different anions and deposition morphology using different anions.^[^ ^150^ ^]^ Copyright 2023, Wiley‐VCH. g) Electrode and SEI morphology after cycling with different anions.^[^ ^151^ ^]^ Copyright 2025, Wiley‐VCH.

Imidazolium‐based ILs have relatively low viscosity and high ionic conductivity: As early as 2010, Yamaki et al.^[^ ^140^ ^]^ reported the application of NaBF_4_/C_2_min[BF_4_] in SIBs, and the use of this IL electrolyte ensures a good thermal stability, which makes the NVP cathode highly safe. Afterward, Wu et al.^[^ ^141^ ^]^ dissolved NaBF_4_ in 1‐ethyl‐3‐methylimidazolium‐bis‐tetrafluoroborate (EMIBF_4_) at different concentrations, and found it had a wide electrochemical window (≈4.0–4.5 V) in the LSV tests, which depended on Na salt concentration (**Figure** **11**a). In practical applications, IL is rarely used on its own, and organic electrolytes are added to reduce the cost and viscosity of IL. Johansson et al.^[^ ^142^ ^]^ added BMImTFSI, EMImTFSI, and Pyr_13_TFSI to EC/PC‐based organic electrolytes, and the ionic conductivity dropped as the IL content increased due to the rapid increase in viscosity. When the addition amount of IL was below 20%, the ionic conductivity was similar to that of the EC/PC‐based electrolyte. By comparison of CV curves, Pyr_13_TFSI added as IL has better sodium plating/stripping behavior, and the hybrid electrolyte enables the HC anode to provide higher capacity. Regardless of the IL type, it can improve the safety of the battery, and the electrolyte is completely non‐flammable when the content is sufficiently high. Understandably, the amount of IL added is a critical factor, and an optimal value needs to be found to balance flammability and ionic conductivity.

Pyrrolidium‐type ILs have a wider electrochemical window than imidazolium‐type ILs, of which the most studied ones are Pyr13 and Pyr14. Meanwhile, pyrrolidium‐type ILs have a greater viscosity, and sacrifice some of the ionic conductivity. Its compatibility with sodium metal is good, and the electrochemical window can reach more than 5 V. Beside the metal anode, the researchers investigated the electrochemical performance of pyrrolidium‐based ILs for HC anodes using NaFSI‐Pyr_13_FSI.^[^ ^143^ ^]^ At a temperature of 363 K, the IL electrolyte renders the HC anode a specific capacity of ≈250 mAh g^−1^ (Figure 11b) and a fast‐charging possibility at a high rate of 4 C. In order to further improve the room temperature performance of pyrrole‐based ILs, many researchers introduce some organic solvents into the electrolyte while preserving the flame retardancy of the electrolyte, such as Passerini et al.^[^ ^144^ ^]^ prepared a hybrid electrolyte by dissolution of Pyr_13_FSI, Pyr_13_TFSI and EC. The addition of mixed ILs can effectively improve the Coulombic efficiency of SIBs compared with the traditional carbonate electrolyte, and the Pyr_13_^+^ cation can form a stable SEI on the HC. However, further research is needed on whether the addition of carbonate solvents to ILs can still ensure the safety of batteries and how far the improvement in electrochemical performance can be improved.

Pyridininium‐type ILs have also gained a lot of interest. Forsyth et al.^[^ ^145^ ^]^ investigated the interfacial chemistry of carbon materials in SIBs in an ultra‐concentrated NaFSI/C_3_mpyrFSI electrolyte. Compared to conventional carbonate electrolytes, IL‐based electrolytes were able to form inorganic‐rich thinner SEI on the surface of the carbon materials due to the decomposition of anions involved in the film formation (Figure 11c). Such an ideal interface enabled the Na||mesoporous carbon (CMK) cell to be cycled stably for 3500 cycles, providing a high reversible capacity of 320 mAh g^−1^ at 0.5 A g^−1^. This work demonstrates that IL can form anion‐derived interfaces compared to ordinary electrolytes due to its anion component, which is crucial for cycle stability. Beyond carbon anode, Chen et al.^[^ ^146^ ^]^ applied IL‐based electrolyte to quinone cathode, which has high capacity and abundant resources while its application is limited due to the fact that quinone cathode is easily soluble in organic electrolyte. This work investigates the inhibition of quinone cathode dissolution by comparing six ILs (Figure 11d). The comparison reveals that IL has a lower donor numbers and fewer interactions than ether electrolytes (DME as solvent). All of them inhibit solvation of the quinone cathode, and the use of IL enables calix[4]quinone materials to have higher capacity and better cycle stability. This work is a good example of how IL can be matched with other SIB materials to find task‐specific applications.

Besides cations, chemistry of the anions has become a research hotspot in recent years, mainly driven by excellent performance of anion‐derived SEI. Different anions have individual properties, among which FSI^−^ and TFSI^−^ have been widely used in the development of new electrolytes due to their excellent film‐forming properties and high conductivity. The similar structure in both raises new questions: 1) which one will provide better battery performance and (2) what is the difference between the two SEIs formed by these two ILs? It has been shown that the presence of FSI‐ has a large impact on the stability of imidazolium‐based IL electrolytes on sodium metal electrodes.^[^ ^147^ ^]^ After storing the sodium metal without oxide layer in NaFSI‐[C_2_C_1_im]FSI and NaTFSI‐[C_2_C_1_im]TFSI, respectively, for 4 weeks, the changes of their electrolytes are shown in Figure 11e. It can be seen that the TFSI based electrolyte turns yellow, which indicates that it reacts with the Na metal, whereas the reactivity of the Na metal in the FSI^−^‐based electrolyte is much lower than that of the TFSI^−^, which may be due to the fact that FSI^−^ can form a dense passivation layer on the sodium metal surface, thus inhibiting the reduction reaction of cations. Wang et al.^[^ ^148^ ^]^ designed a new electrolyte by dissolving sodium salt in a liquid precursor of salt (SIPS), while in the selection of sodium salt they pointed out that FSI^−^ anion can form more NaF‐rich inorganic SEI than TFSI^−^ anion, and therefore NaFSI was chosen as the salt in the SIPS. Moreover, Ming et al.^[^ ^149^ ^]^ compared TFSI^−^ and FSI^−^ in the same solvent system and found that FSI^−^ exhibited better electrochemical performance than TFSI^−^.Through the construction of the model, researchers found that the difference in performance originated from the different solvation structures and their different interfacial behaviors. Compared to TFSI^−^, FSI^−^ has stronger interactions with cations; it weakened the solvent‐cation interactions and therefore accelerated the desolvation process and formation of an anion‐rich solvation structure, which facilitates the deposition of cations at the interface. Similarly, considering the structural similarity, researchers proposed a new asymmetric structure of FTFSI^−^ anion by comparing TFSI^−^ and FSI^−^,^[^ ^150^ ^]^ through which it was found that this asymmetric anion was able to enhance the interaction with the solvent, and weaken the coordination of the solvent and the cation, facilitating the reversible deposition and stripping of the cation (Figure 11f). Besides FSI^−^ and TFSI^−^, Xu et al.^[^ ^151^ ^]^ compared the application of OTF^−^, TFSI^−^, PF_6_^−^, and ClO_4_^−^ in SIBs, in which OTF^−^ has a high donor number and a high binding energy with Na^+^. Thus OTF^−^ can form a solvation structure dominated by the OTF^−^ anion, which makes the OTF^−^‐derived SEIs rich in inorganic compounds, e.g., NaF and Na_2_O, with a dense and homogeneous structure (Figure 11g), and favors Na^+^ transport and long‐term stability. In summary, FSI^−^ can form more inorganic‐rich SEIs with better overall performance than TFSI^−^ due to a more optimal solvation structure. The nature of the different interfacial behaviors derived from different anions may be originated from the difference in solvation structure. It encourages researchers to investigate the cation‐anion and solvent‐anion interactions in future.

Some groups mixed IL with co‐solvents to prepare locally highly concentrated electrolytes. In some metal anodes, since previous studies have demonstrated that the cations in IL do not participate in the solvation structure, so that the cations in IL produce an effect similar to electrostatic shielding on the surface of the metal anode, promoting uniform deposition.^[^ ^152^, ^153^, ^154^ ^]^ Such a multifunctional effect promotes the use of IL. One of the co‐solvents is often the fluorinated ethers. For example, Wu et al.^[^ ^155^ ^]^ formulated a localized high‐concentration electrolyte, namely N‐methyl‐N‐propylpyrrolidinium bis(fluorosulfonyl)imide (Py_13_FSI) as solvents and 1,2‐bis (1,1,2,2‐tetrafluoroethoxy) ethane (TFEE) as co‐solvent. The introduction of co‐solvents is beneficiary as it reduces the viscosity, and improves the ionic conductivity, and the overall non‐flammability of the electrolyte. The presence of abundant anions in the electrolyte due to the introduction of IL leads to a solvated structure of anionic coordination, which forms an anion‐derived inorganic SEI at the anode.

In SIBs, ILs have been used relatively rarely, mainly because of their high cost, which is contrary to the merits of SIBs. Moreover, SIBs have a faster kinetic rate than LIB, and this merit is forfeited when IL is introduced due to the increase in viscosity because IL addition affects the migration of Na^+^. The advantages of IL are thermal stability and its capability of forming interfaces with excellent performance, besides its structural diversity to obtain a huge structure library for task‐specific electrolyte systems. In order to maintain the advantages of IL without increasing the cost, IL can be introduced in a small amount, saying 5 wt%, as an additive to enhance the cell performance.

### 3.3 Aqueous Electrolyte

**Figure 12:**
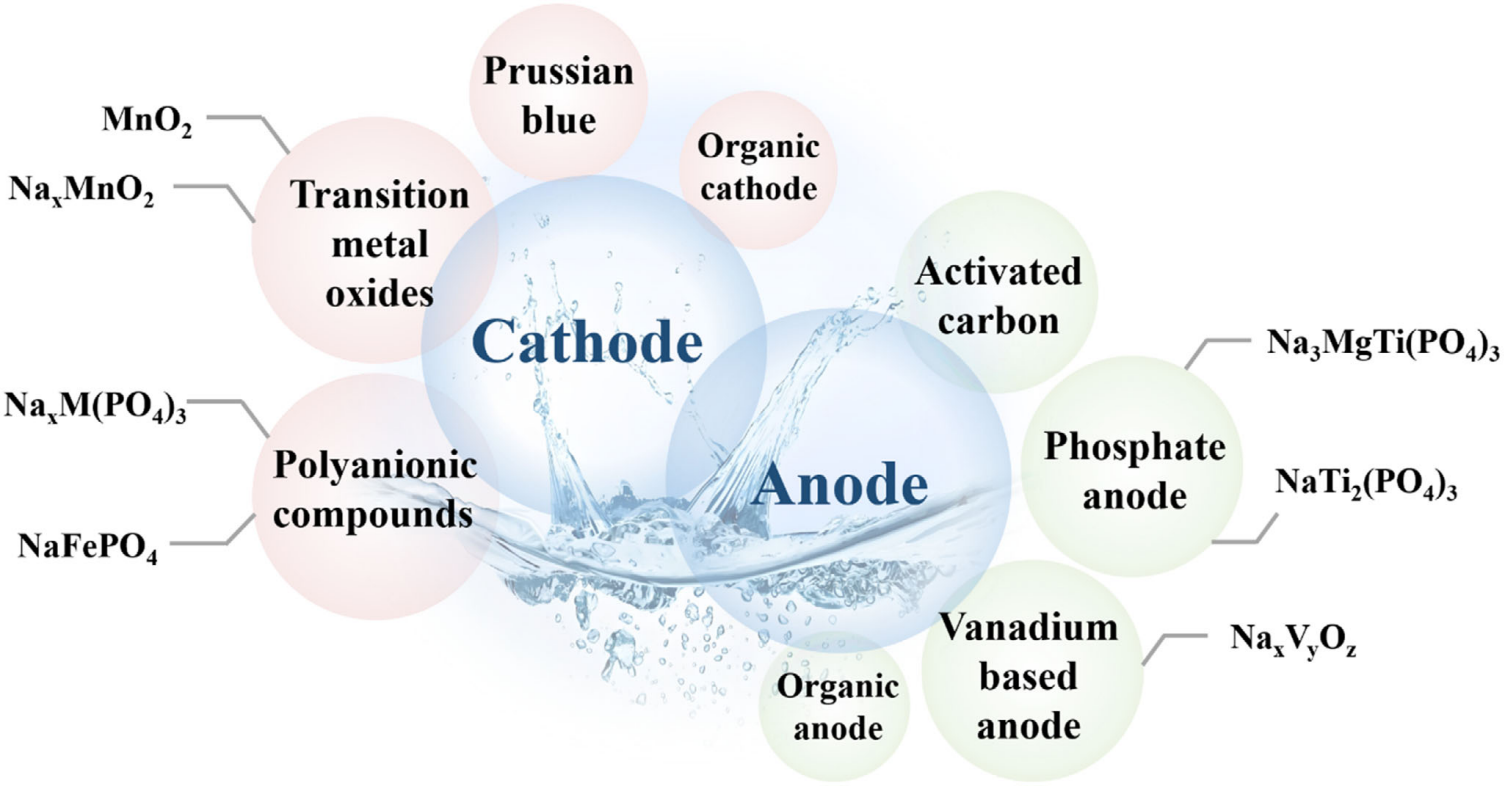
Common anode/cathode materials for ASIBs.

Due to the flammability of organic electrolytes, battery safety issues have always been urgent. Developing non‐flammable electrolytes is the fundamental way to solve this problem. Compared with ILs or non‐flammable organic electrolytes, the aqueous electrolyte is of a lower cost, environmentally friendly, and also a high ionic conductivity, so that it is considered as a promising electrolyte system.^[^ ^156^ ^]^ Currently, aqueous electrolytes are often applied in zinc ion batteries (ZIBs), while there are also a number of studies in LIBs and SIBs. Compared to organic electrolytes, aqueous sodium‐ion batteries (ASIBs) have been less studied and developed slowly. This is mainly due to the limitations of water solvent. First, the aqueous solution decomposes, leading to side reactions of hydrogen/oxygen evolution. Additionally, the electrochemical window of aqueous electrolytes is 1.23 V, which leads to the fact that the cathode voltage of ASIBs can't be too high, and the anode voltage can't be too low. This problem has limited the application scope of materials in ASIBs.^[^ ^54^, ^157^, ^158^ ^]^ Some of the electrode materials and their intermediates formed during charging and discharging processes are prone to reaction with H_2_O or O_2_, which can lead to failure of the electrode materials. This requires chemical and physical stability of the electrode materials in aqueous solution. Moreover, due to the presence of side reactions in aqueous solution, such as hydrogen evolution, oxygen evolution, co‐embedding of protons or water molecules, it is difficult to control the pH of the electrolyte, which requires the electrode materials of ASIBs to be stable at a broad range of pH values.^[^ ^159^ ^]^ Finally, the film‐forming ability of the aqueous electrolyte is much weaker, which limits the stability of the electrode materials during long cycling.^[^ ^160^ ^]^ Considering the above‐mentioned problems, the electrode materials applied in ASIBs are very restricted, as listed in **Figure** **12**. The cathodes mainly include manganese oxides, polyanionic compounds, Prussian blue analogues, and organic polymer materials, while the anodes are dominated by activated carbon (AC), polyanionic anodes, vanadium‐based anodes and organic anodes.

**Figure 13:**
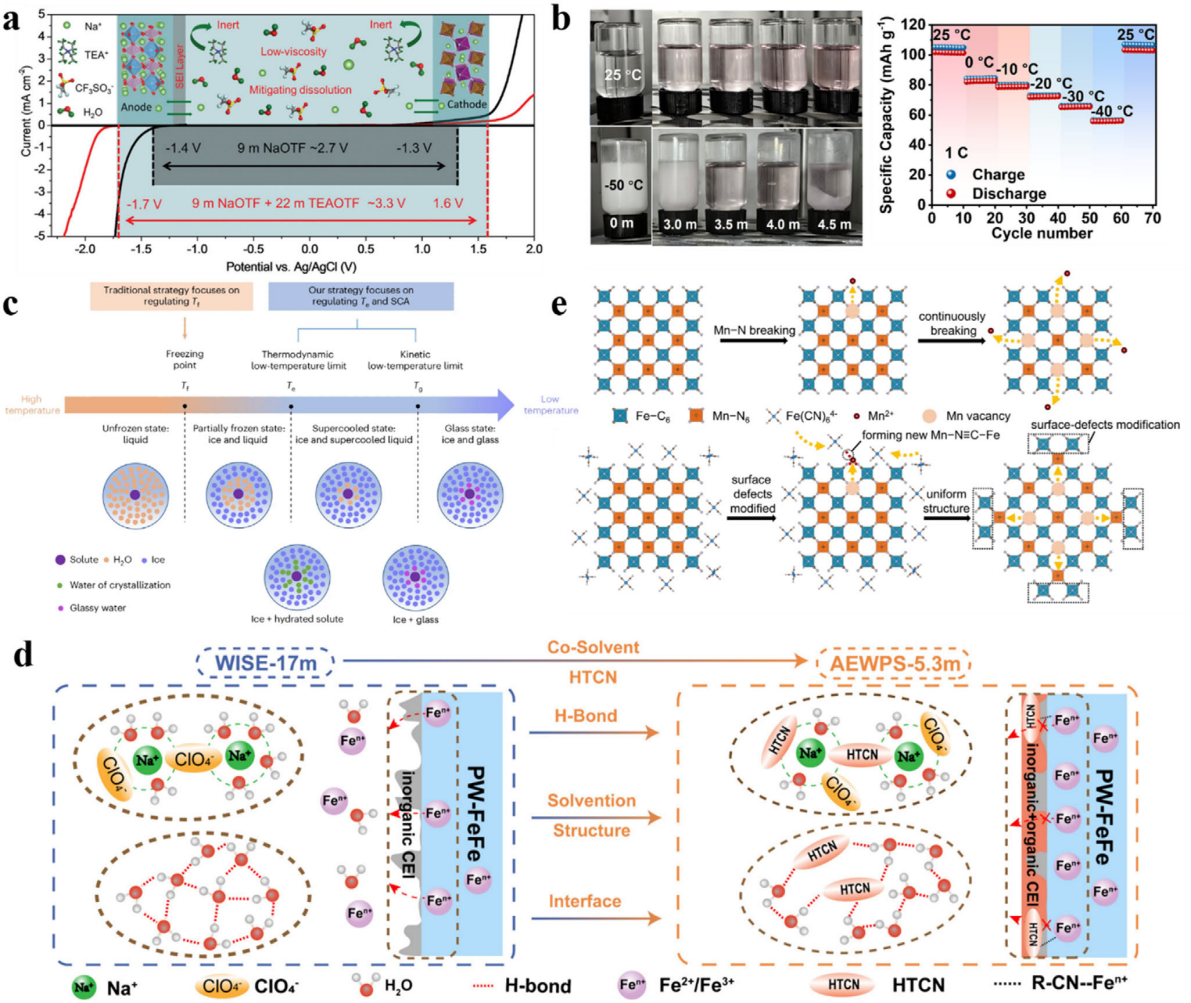
a) High concentration electrolyte (9 m NaOTF +22 m TEAOTF) broadens the electrochemical window to 3.3 V while maintaining low viscosity.^[^ ^168^ ^]^ Copyright 2019, Wiley‐VCH. b) 0.5 m NaCl + 4.0 m MnCl_2_ 4H_2_O electrolyte maintains liquid state at −50 °C and enables Na_2_CoFe(CN)_6_||AC full cell to operate at −40 °C.^[^ ^174^ ^]^ Copyright 2024, Elsevier. c) Physical changes from high to low temperatures of dilute solutions in the H_2_O‐solute system^[^ ^177^ ^]^ Copyright 2024, Springer Nature. d) Schematic diagram of the action mechanism of HTCN in electrolyte.^[^ ^180^ ^]^ Copyright 2024, American Chemical Society. e) Schematic diagram of the cation capture process by additives.^[^ ^181^ ^]^ Copyright 2023, Springer Nature.

Similar to organic electrolytes, ASIB electrolytes consist of sodium salts, aqueous solvents, and additives, but the choice of sodium salts in aqueous electrolytes varies somewhat. Na_2_SO_4_, NaCl, and NaNO_3_ have been reported, with 1 M Na_2_SO_4_ in H_2_O being the most common, though some other reports employ NaClO_4_ and NaOH. For example, as early as 2012, Whitacre et al.^[^ ^161^ ^]^ assembled an ASIB consisting of 1 M Na_2_SO_4_ in H_2_O as electrolyte, MnO_2_ as cathode, and activated carbon as anode. This electrolyte formulation is difficult to solve the problem in association with the narrow electrochemical window, so that researchers start to design high‐concentration aqueous electrolytes. This strategy is taken from aqueous LIBs, and increasing salt concentration can effectively expand the electrochemical window of the electrolyte and stabilize electrode materials.^[^ ^162^, ^163^ ^]^ Due to the high solubility of sodium salts in water, researchers have investigated aqueous electrolytes at ultra‐high salt concentrations. For example, Okada et al.^[^ ^164^ ^]^ set the concentration of NaClO_4_ at 17 m. The electrochemical window was extended from 1.9 V (as limited by the concentration of 1 M) to 2.8 V. The use of 17 m NaClO_4_ aqueous electrolyte opens up a voltage of 2 V for Na_2_Mn[Fe(CN)_6_]||KMn[Cr(CN)_6_] cells. Recently, the “water in salt” electrolyte has been proposed, which defines a state of electrolyte at a concentration of sodium salt higher than 9 m. At such state, the interaction between anions and sodium ions in the solvation structure is strong, which weakens the coordination between water molecules and Na^+^. This unique solvated structure promotes the anion reduction and the formation of a stable SEI film on the electrode surface to widen the electrochemical window.^[^ ^165^, ^166^, ^167^ ^]^ In consideration of the relatively lower solubility of Na^+^ salts than Li^+^ and K^+^ salts, salts carrying large‐sized cations are often introduced to enhance the solubility. For example, Suo et al.^[^ ^168^ ^]^ mixed tetraethylammonium trifluoromethanesulphonate (TEASO_3_CF_3_) with NaSO_3_CF_3_ at 22 m and 9 m, respectively, to design an anion‐rich ultra‐high concentration aqueous electrolyte. Through this strategy the viscosity is lower than other high‐concentration electrolytes, and meanwhile extends the electrochemical window up to 3.3 V (**Figure** **13**a), rendering the NaMnFe(CN)_6_||NaTiOPO_4_ cell with good cycle stability due to the participation of anions in film formation.

In aqueous electrolytes, the existence of a high number of hydrogen bonds between water molecules results in a high freezing point, solidification at low temperatures (<0 °C), and slower ion diffusion.^[^ ^169^, ^170^, ^171^ ^]^ In some studies, the salt concentration was increased to extend the electrochemical window at the expense of aqueous electrolytes capable of operating at low temperatures. Consequently, it is crucial to explore aqueous electrolytes working at low temperatures in order to adapt a wider range of operation environments. Encompassing the low‐temperature range, organic solvents of low melting points are often mixed,^[^ ^172^, ^173^ ^]^ which mitigates the original intention of using aqueous electrolytes, being non‐flammable and environmentally friendly. Therefore, researchers have proposed other strategies. For instance, He et al.^[^ ^174^ ^]^ introduced MnCl_2_·4H_2_O into aqueous electrolytes. Because of the strongly interacting Mn^2+^ and water molecules, the hydrogen bonding network of water is disrupted, thereby inhibiting the solidification of aqueous solutions at low temperature. This strategy enables the mixed electrolyte to remain liquid at −50 °C and has high ion‐conductivity. Using this electrolyte, the assembled Na_2_CoFe(CN)_6_||AC full cell was able to operate properly at −40 °C and reached 54.9% of the room temperature capacity (Figure 13b). Similarly, other inorganic antifreeze agents, such as CaCl_2_.^[^ ^175^, ^176^ ^]^ with the aim of breaking the hydrogen bond networks to widen the temperature range of aqueous electrolytes. Recently, Hu et al.^[^ ^177^ ^]^ proposed general guidelines for designing low‐temperature aqueous electrolytes based on an in‐depth understanding of H_2_O‐solute equilibrium. It points out that the low eutectic temperature (*T*_e_) and kinetic glass‐transition temperature (*T*_g_) is more important than that of low freezing point (*T*_f_), as shown in Figure 13c, where *T*_f_ varies when the salt concentration changes, but the value of *T*_e_ is constant; the mixture can still maintain a sufficiently high ionic conductivity before it completely freezes into a crystalline state of *T*_e_. In addition, having a strong super‐cooling ability (SCA) can maintain a supercooled liquid state for a long time at temperatures between *T*_e_ and *T*_g_. These factors guide us in designing aqueous electrolytes with lower *T*_e_ and *T*_g_. Experimentally, it was found that the introduction of some cations with high ionic potentials and solvents with high donor number could reduce T_e_ and T_g_. This recent study designed a series of low‐temperature electrolytes by mixing Al^3+^, Ca^2+^, and ethylene glycol (EG) with aqueous electrolytes, which achieved low *T*_e_ (−53.5 to 72.6 °C) and low *T*_g_ (−86.1 to −117.1 °C); the assembled NaFeMnHCF/Na‐H_2_O‐Ca/PTCDI pouch cell is capable of operation at −80 °C. This study proposes not only a low‐temperature aqueous electrolyte, but more importantly, a guiding principle for low‐temperature electrolyte design, which supports aqueous batteries operating in extreme environments.

In addition to solvents, additives are often used in aqueous electrolytes to enhance the performance of batteries. Kumar et al.^[^ ^178^ ^]^ added 2 vol% of VC to an aqueous electrolyte of 10 M NaClO_4_, which effectively widened its electrochemical window. Some additives are able to form a protection layer on the electrode surface to isolate the electrode material from the reaction with O_2_ and prevent the corrosion reaction that occurs in highly concentrated electrolytes. Additional functional additives are also included. For example, some inorganic salts or small organic molecules as additives can disrupt the hydrogen bonding network in water to improve low‐temperature electrical conductivity and decrease activity of the water, thus widening the electrochemical window.^[^ ^179^ ^]^ You et al.^[^ ^180^ ^]^ introduced weakly polar hexanetricarbonitrile (HTCN) into an aqueous electrolyte to broaden the electrochemical window of the electrolyte to 3.5 V. HTCN partially replaced ClO_4_^−^ in the solvation structure and acted as a hydrogen‐bonding donor to weaken the hydrogen‐bonding network in solution. Due to the regulation of the solvation structure by HTCN, a homogeneous and stable inorganic/organic hybrid CEI was formed, to inhibit the dissolution of transition metal Fe in the electrode material (Figure 13d). To note, there are some task‐specific additives. Wang et al.^[^ ^181^ ^]^ proposed a cation capture method that introduces sodium ferrocyanide (Na_4_Fe(CN)_6_) as a support salt in a high concentration (17.6 m) NaClO_4_‐based aqueous electrolyte to in situ repair the Mn vacancies formed during the cycling process of Na_1.58_Fe_0.07_Mn_0.97_Fe(CN)_6_·2.65H_2_O (NaFeMnF) (Figure 13e); the additive has no effect on the solvated structure while effectively mitigating the structural distortion of the material brought by the Jahn‐Teller effect.

The cost and environmental advantages of aqueous electrolytes make them attractive, and preparation costs have been significantly reduced due to tolerance to air and water. However, the research on ASIB is still very limited, especially at the solid‐liquid interface. The passivation layer on the electrode surface in ASIB not only improves the cycling stability, but also widens the electrochemical stability window. The different anions and solvents in the electrolyte determine the structural properties of the passivation layer and the ion transport capacity, so to improve the performance of ASIB. It is necessary to select the ideal solvent to balance the pH and remove the dissolved oxygen in water. On top of that, additives can be developed that are more suitable for ASIB. From the perspective of function, the additives should be able to improve the interface stability and inhibit the side reactions of high‐concentration electrolytes. The main reason why ASIB is difficult to apply is the narrow electrochemical window and the specificity of the electrode material. Although a high salt concentration can widen the electrochemical window, it amplifies the cost. How to enhance the electrochemical window without using electrolytes at ultra‐high concentration is believed to be a key issue for future ASIB research. For electric vehicles or consumer battery products, high energy density is crucial, so large‐scale energy storage may be a more suitable scenario for ASIBs.

### 3.4 Gel Electrolytes

Solid‐state electrolytes are considered to be one of the complete solutions to battery safety. The associated challenge is the low ionic conductivity and high interfacial impedance, which affect ion migration.^[^ ^182^, ^183^, ^184^ ^]^ Therefore, it has been proposed to use polymers to solidify the electrolyte to form a gel electrolyte, which combines the merits of the safety of a solid electrolyte and the transport properties of a liquid electrolyte. It first originated from Feuillade et al.’s attempt in 1975^[^ ^185^ ^]^ to introduce a non‐protonic solvent containing metal salts into a polymer‐alkali metal salt electrolyte, thus forming a gel electrolyte. Gel electrolytes are obtained by swelling the polymer after absorbing a liquid electrolyte, which maintains the shape of a solid and has a high ionic conductivity. The main methods of preparation include physical mixing, sol–gel, cross‐linking, and electrostatic spinning.^[^ ^186^ ^]^ In SIBs, the development and application of gel electrolyte is much less than that of organic electrolytes. However, due to the advantages in safety, it has gained more and more attention recently. Gel polymer electrolytes (GPEs) consist of a polymer matrix and plasticizers, as well as salts dissolved in them. Common gel polymer matrices include polyethylene oxide (PEO), polyvinylidene difluoride (PVDF), poly(methyl methacrylate) (PMMA), and cyanide polymers, while plasticizers are typically organic solvents with high dielectric constants, such as EC, PC, and NMP.^[^ ^187^ ^]^

PEO‐based gel electrolytes are the earliest and most widely studied polymer matrices. Their main drawback is their low ionic conductivity at room temperature. Adding organic solvent plasticizers to PEO to prepare gel electrolytes can effectively improve ionic conductivity. When crown ethers are used as plasticizers, they not only improve conductivity but also effectively reduce the impedance at the electrode‐electrolyte interface.^[^ ^188^ ^]^ PEO‐based polymers have poor mechanical properties and thermal stability at high temperatures, which can be alleviated by blending, cross‐linking, or adding inorganic fillers. For example, Li et al.^[^ ^189^ ^]^ prepared a gel electrolyte with a dense structure, strong mechanical properties, and good flexibility by cross‐linking polyethylene glycol diglycidyl ether (PEGDE) and diaminopolypropylene oxide (DPPO), ensuring uniform deposition and stripping of Na^+^. PVDF‐based gel electrolytes are also widely used due to their good film‐forming properties, high dielectric constant, and high glass transition temperature. To address the issues of low ionic conductivity and mechanical strength, researchers first proposed the use of copolymerization of vinylidene difluoride with hexafluoropropylene (HFP) in 1999, which was then applied to LIBs.^[^ ^190^ ^]^ Subsequently, in 2010, Hashmi et al.^[^ ^191^ ^]^ synthesized a structurally stable gel electrolyte using NaOTF as the Na salt, PVDF‐HFP as the copolymer matric, and EMIOTF as the IL. Its electrochemical window reached 5 V, with an ionic conductivity as high as 5.74 × 10^−3^ S cm^−1^ (27 °C). PMMA‐based polymers are cost‐effective and easy to prepare. They contain a large number of ester groups, which confer good compatibility with many organic solvents and enable them to absorb electrolytes, thereby enhancing ionic conductivity. However, PMMA‐based polymers are brittle. They are typically blended or crosslinked with other polymers. For example, Goodenough et al.^[^ ^192^ ^]^ used 2,2′‐azobis(2‐methylpropionitrile) (AIBN) as an initiator to synthesize crosslinked PMMA from methyl methacrylate (MMA) and tetraethylene glycol dimethacrylate (TEGDMA), thereby improving the mechanical strength of the electrolyte and enhancing ionic conductivity. Additionally, due to the high polarity of cyano‐groups, incorporating them into the polymer matrix effectively increases the oxidation decomposition potential of the electrolyte. Common cyano‐containing polymer gel electrolytes include polyacrylonitrile (PAN), cyanoethylated polyvinyl alcohol (PVA‐CN), and polycyanoacrylate ethyl ester (PECA).^[^ ^193^, ^194^, ^195^ ^]^

**Figure 14:**
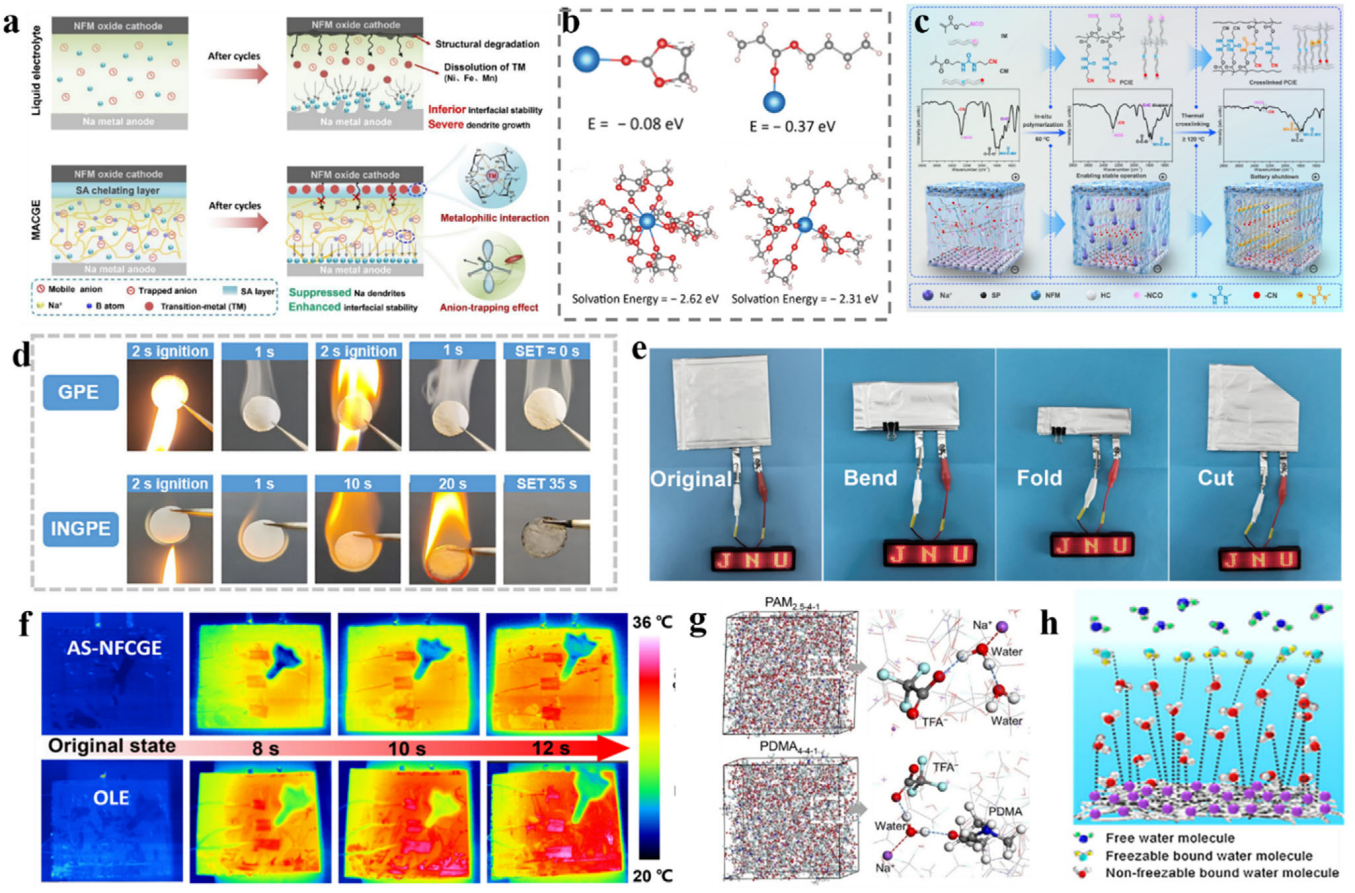
a) Schematic diagram of the modification mechanism of MACGE on anode and cathode.^[^ ^196^ ^]^ Copyright 2023, Elsevier. b) Comparison of typical solvated structures of liquid electrolyte (left) and gel electrolyte (right).^[^ ^197^ ^]^ Copyright 2022, Wiley‐VCH. c) Schematic diagram of thermal response crosslinking mechanism of PCIE.^[^ ^198^ ^]^ Copyright 2025, Springer Nature. d) Self‐extinguishing time experiments of GPE and INGPE.^[^ ^199^ ^]^ Copyright 2025, Wiley‐VCH. e) Operation of LED lights under abnormal working conditions.^[^ ^200^ ^]^ Copyright 2024, Elsevier. f) Temperature changes in pouch cells using OLE and AS‐NFCGE under puncture testing.^[^ ^200^ ^]^ Copyright 2024, Elsevier. g) Solvation structure of water molecules in PAM 2.5‐4‐1and PDMA 4‐4‐1.^[^ ^203^ ^]^ Copyright 2023, Wiley‐VCH. h) Schematic diagram of super‐hydrophilic hydrogel electrolyte strategy.^[^ ^204^ ^]^ Copyright 2024, American Chemical Society.

In addition to common polymer matrices, many studies have developed novel gel electrolytes using different polymers. Recently, research on gel electrolytes has moved beyond single‐aspect improvements. For example, Liu et al.^[^ ^196^ ^]^ reported a bifunctional B‐containing gel electrolyte (MACGE) modified by sodium alginate (SA), in which the B‐containing polymer captures anions through Lewis acid‐base interaction, thereby reducing concentration polarization at the anode interface and promoting uniform Na deposition. The grafted SA is rich in carboxyl groups, which can effectively inhibit the dissolution of transition metals on the cathode side through chemical chelation (**Figure** **14**a), improving the cycle stability. As a result, the assembled NFM||Na cell retains 85.5% of its capacity after 800 cycles at 3 C. Tao et al.^[^ ^197^ ^]^ dissolved butyl acrylate (BA) and AIBN initiator in a liquid electrolyte, then polymerised them in situ to form a multifunctional poly(butyl acrylate) (PBA)‐based gel electrolyte. It exhibits an excellent room‐temperature ionic conductivity of 1.6 mS cm^−1^ and inhibits the formation of Na dendrites. Additionally, DFT calculations show that BA has a higher binding energy with Na^+^ (−0.37 eV vs −0.08 eV) compared to EC in liquid electrolytes, enabling it to replace EC in the solvation structure. Due to the larger steric hindrance of BA molecules, the solvent content in the entire solvation structure decreases, thereby reducing the desolvation energy (Figure 14b), improving the Na^+^ diffusion rate. In addition to the common functions of interface stability, solvent structure regulation, and conductivity enhancement, Cui et al.^[^ ^198^ ^]^ developed a smart gel polymer electrolyte (PCIE) based on in situ polymerization of cyanoethylurea‐containing methacrylate monomer (CM) and isocyanate‐based methacrylate monomer (IM). This PCIE not only forms a stable SEI/CEI but also possesses a smart thermal response cross‐linking mechanism (Figure 14c): When the temperature exceeds 120 °C, nucleophilic addition reaction will occur between the urea group and isocyanate group in the electrolyte, leading to further cross‐linking reactions, thereby increasing the glass transition temperature of the polymer, reducing free volume, and blocking ion transport. Additionally, the cross‐linked electrolyte will inhibit the gas cross effect, enabling the HC||O_3_–NaNi_1_/_3_Fe_1_/_3_Mn_1_/_3_O_2_ cell to exhibit not only long‐term cycling stability but also enhanced safety at high temperatures. This work provides important insights into the development of multifunctional gel electrolytes.

Considering safety, many researchers opt for non‐flammable phosphate esters as solvents for preparing gel electrolytes. For instance, Jiao et al.^[^ ^199^ ^]^ blended ethoxylated trimethylpropane triacrylate (ETPTA) with triethyl phosphate (TEP) and FEC to create a flame‐retardant gel electrolyte (AS‐NFCGE). The carbonyl group on ETPTA promotes uniform migration of Na^+^, FEC facilitates the formation of a stable F‐rich interface, and TEP enhances the flame‐retardant properties of the electrolyte, enabling the GPE to remain unignited after a 2 s ignition attempt, with a self‐extinguishing time approaching 0 s (Figure 14d). Similarly, Liu et al.^[^ ^200^ ^]^ prepared a flame‐retardant gel electrolyte using PEGDA cross‐linker, AIBN initiator, and TEP‐based carbonate electrolyte. To verify its safety in practical applications, pouch batteries were assembled, as shown in Figure 14e, which enabled the LED lights to function normally under bending, folding, or even cutting. During puncture tests, the temperature at the puncture site only slightly increased (Figure 14f), demonstrating the high safety of the prepared gel electrolyte in practical applications.

Apart from some organic solvents, researchers combined the concepts of aqueous batteries and gel electrolytes to prepare hydrogel electrolytes. The hydrogel electrolyte has certain flexibility but low mechanical strength, and water molecules cause the same side reactions as in aqueous electrolytes, such as hydrogen evolution and metal ion dissolution. The electrochemical window of hydrogel electrolyte is narrow.^[^ ^201^, ^202^ ^]^ Increasing the salt concentration can effectively solve this problem. For example, Cui et al.^[^ ^203^ ^]^ eliminated hydrogen bonding interactions within and between polymer molecule chains through methylation modification, thereby enhancing the solvation capacity of Na^+^ in the polymer and promoting the dissolution of Na salts, ultimately achieving a high salt concentration (44 mol%). This method broadens the electrochemical window. According to MD simulation (Figure 14g), PAM 2.5‐4‐1 contains aggregated water molecules that cannot be ignored. When the salt concentration increases (PDMA 4‐4‐1), the water molecules become isolated, and the O atom coordinates with Na^+^, reducing the water molecules that form hydrogen bonds, thus inhibiting the side reactions. The assembled Na_3_V_2_(PO_4_)_3_||NaTi_2_(PO_4_)_3_ full cell has a long cycle life. Moreover, Zhi et al.^[^ ^204^ ^]^ proposed a method to prepare super‐hydrophilic hydrogel electrolytes, transforming water molecules in the gel electrolyte into bound water (Figure 14h), reducing free water molecules, thereby inhibiting decomposition and broadening the electrochemical stability window of the gel electrolyte. The gel electrolyte developed by this method can be used in a variety of batteries (LIB, SIB, and potassium‐ion battery). Hydrogel is often used in flexible/stretchable electronic products because of its flexibility and elasticity. However, when applied to secondary batteries, it is necessary to solve the problem of deformation or rupture. In addition, although hydrogel electrolytes have higher ionic conductivity, side reactions and a narrower electrochemical window further limit their application in SIBs.

**Figure 15:**
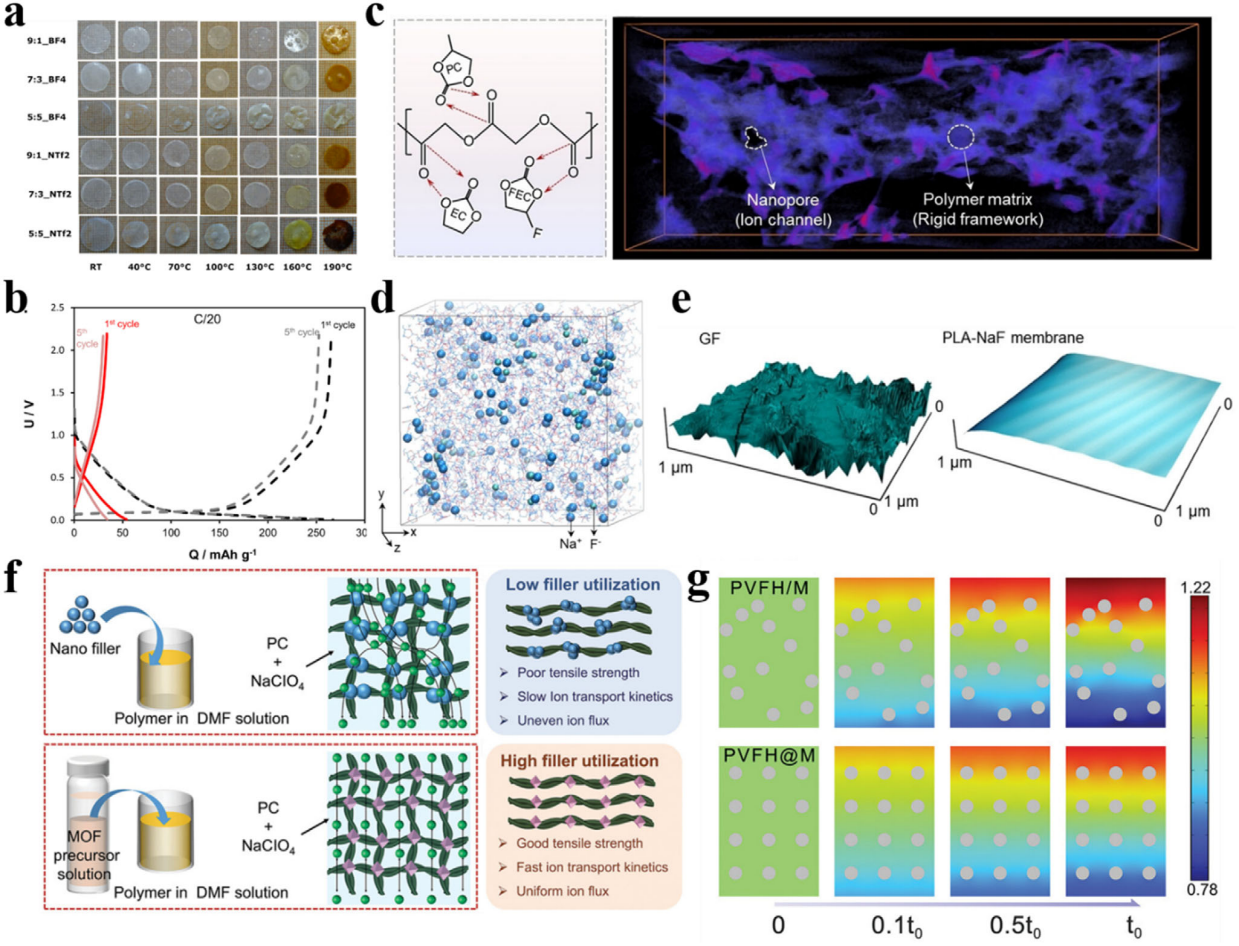
a) Thermal dimensional shrinkage of gel electrolytes with different ratios.^[^ ^205^ ^]^ Copyright 2023, Elsevier. b) Sodium storage performance of HC||Na half cell with different gel electrolytes.^[^ ^205^ ^]^ Copyright 2023, Elsevier. c) Dipole interaction and 3D structure of gel electrolyte.^[^ ^207^ ^]^ Copyright 2024, Wiley‐VCH. d) Solvation environment of PLA‐NaF‐GPE.^[^ ^207^ ^]^ Copyright 2024, Wiley‐VCH. e) Comparison of the uniformity of different electrolyte membranes.^[^ ^207^ ^]^ Copyright 2024, Wiley‐VCH. f) Comparison of gel electrolytes synthesized by the traditional method and the solution processable method.^[^ ^208^ ^]^ Copyright 2024, Wiley‐VCH. g) Finite element simulation of the electric field distribution of gel electrolytes synthesized by different methods.^[^ ^208^ ^]^ Copyright 2024, Wiley‐VCH.

ILs can be added to polymer electrolytes as a plasticizer to obtain gel electrolytes, and the performance of gel electrolytes containing ILs is also enhanced. For example, Gabryelcyzk et al.^[^ ^205^ ^]^ used different ratios of polyacrylonitrile and poly(methyl methacrylate) (PMMA) to make up a polymer matrix, and used ionic liquid plasticizers carrying the mixture of sulfolane and imidazolium‐based cations and BF_4_^−^ or NTf_2_^−^ anions to form gel electrolytes. By evaluating the thermal dimensional shrinkage of the electrolyte (**Figure** **15**a), it was found that the more PMMA in the polymer matrix, the softer the electrolyte becomes and the more likely it is to break at high temperatures. The study on the compatibility of HC anodes revealed that sulfolane/[EMIm][NTf_2_] as a binary plasticizer favors the gel electrolytes in SIBs, to enhance the Na^+^ storage performance of HC anodes (Figure 15b).

One of the limiting factors in the use of gel electrolytes in SIBs is interfacial compatibility. This mainly includes the contact of different electrode materials with the electrolyte and the formed SEI/CEI.^[^ ^206^ ^]^ Chen et al.^[^ ^207^ ^]^ dissolved NaF in a porous fibrous framework of polylactide (PLA) to establish highly compatible interfaces by modifying the solvation structure. There is a strong dipole‐dipole interaction between PLA polymer chains and solvent molecules, and the 3D structure of the electrolyte was confirmed by cryogenic TEM (Figure 15c), which includes nanopores for fast ion transport. Through molecular dynamics simulation, NaF can be completely dissolved in the electrolyte, and the PLA‐NaF membrane has a flatter morphology, which gives it better contact with the electrode (Figure 15d,e). As verified by experiments, the gel electrolyte designed in this work is compatible with a variety of cathodes, such as NVP, Na_2+2x_Fe_2‐x_(SO_4_)_3_, and Na_0.72_Ni_0.32_Mn_0.68_O_2_, and anodes such as FeS_2_@N and HC. The high compatibility greatly broadens the application scope of this gel electrolyte.

Zhang et al.^[^ ^208^ ^]^ proposed an innovative in situ solution method for the preparation of gel electrolytes, as shown in Figure 15f. It enhances the cycle stability of SIBs by improving the tensile strength and facilitating rapid ion transport. This approach solves the issues of aggregation and low utilization of traditional nanofillers. The electric field distribution of the gel electrolytes produced by different preparation methods under a certain current is simulated by finite element, as shown in Figure 15. It is observed that the solution‐processable method can ensure the homogeneity of the electric field of the cell, thus inducing the uniform deposition of Na^+^.

Although the application of gel electrolytes in SIBs has attracted interest recently, their high cost and relative complexity in preparation for practical applications necessitate the establishment of innovative preparation methods. If this problem can be solved and the interface can be optimized, gel electrolytes will have a bright future in SIBs.

### 3.5 Solid Electrolyte

Solid electrolytes are believed to be able to fundamentally solve the safety issues of batteries, and solid electrolytes for sodium batteries originated from the application of Na–β‐Al_2_O_3_ to Na–S batteries by Kummer et al.^[^ ^209^ ^]^ in 1967. So far, researchers have been exploring various solid electrolytes. While at present, solid‐state sodium batteries are still in the laboratory research stage, which are mainly divided into two kinds: inorganic solid electrolytes and polymer solid electrolytes.

#### 3.5.1 Inorganic Solid Electrolytes

Inorganic solid electrolytes include β‐Al_2_O_3_ solid electrolytes, NASICON‐type solid electrolytes, sulfide solid electrolytes, etc. Conductivity is an important parameter of solid electrolytes, and depends on the type of electrolytes. Sulfide solid‐state electrolytes usually have the highest conductivity, followed by NASICON materials. While doping can further improve their conductivity, it often comes at the expense of good interfacial contact, potentially leading to high interfacial impedance. In comparison, some oxide solid electrolytes, such as β‐Al_2_O_3_, require higher temperatures to exhibit good conductivity.^[^ ^210^, ^211^ ^]^ The following sections describe these solid electrolytes in detail.

##### β‐Al_2_O_3_ Solid Electrolyte

**Figure 16:**
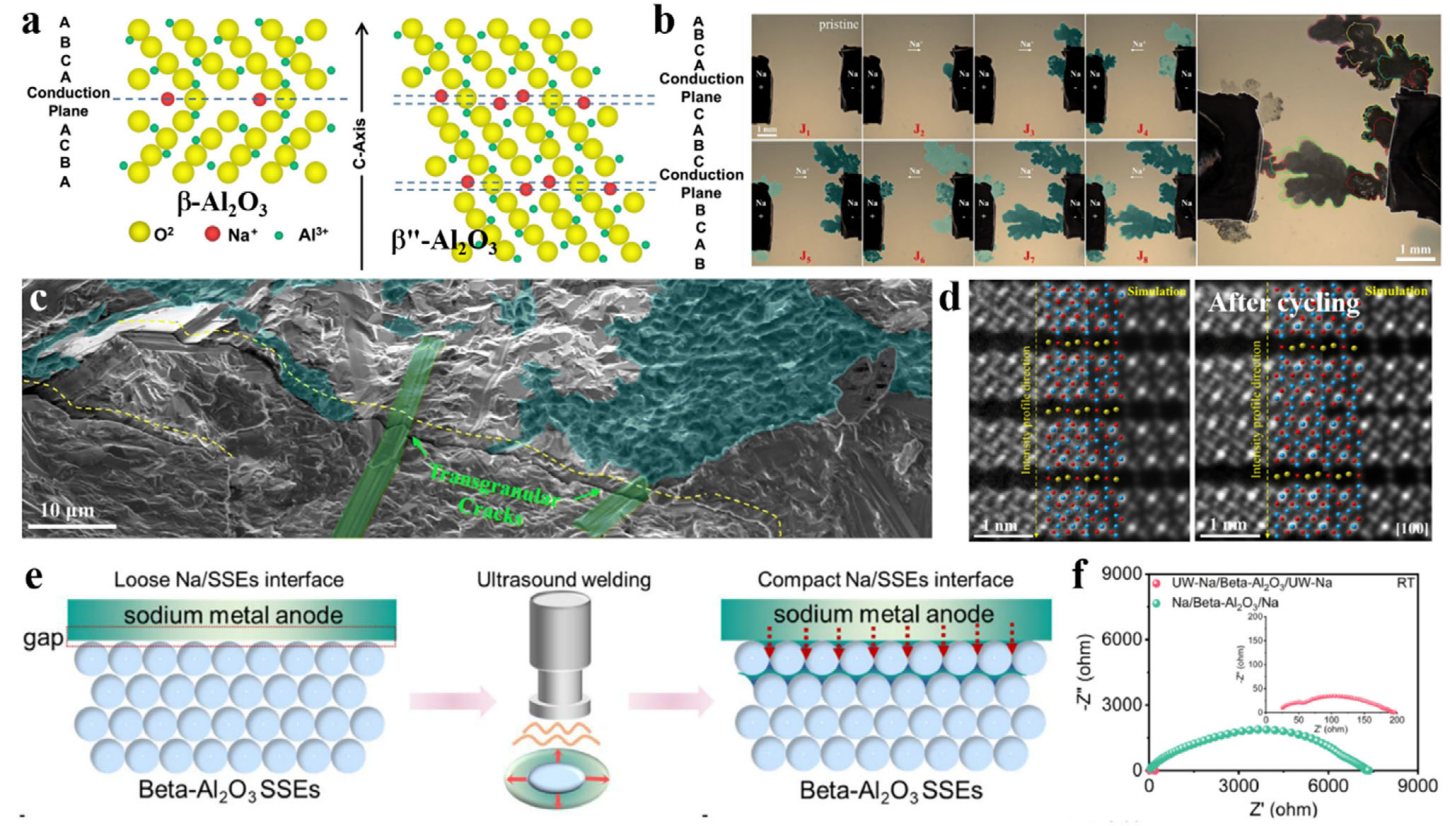
a) Schematic diagram of stacking sequence and ion conduction plane for β‐ Al_2_O_3_ and β“‐Al_2_O_3_.^[^ ^219^ ^]^ Copyright 2018, Elsevier. b) Observation of sodium dendrite growth process in symmetric cells using β”‐Al_2_O_3_ electrolyte by in situ optical microscope.^[^ ^217^ ^]^ Copyright 2023, Royal Society of Chemistry. c) SEM images of sodium dendrites and cracks.^[^ ^217^ ^]^ Copyright 2023, Royal Society of Chemistry. d) HAADF‐TEM image of the initial and cyclic β"‐Al_2_O_3_ structure.^[^ ^217^ ^]^ Copyright 2023, Royal Society of Chemistry. e) Schematic illustration of ultrasonic welding strategy.^[^ ^218^ ^]^ Copyright 2023, Elsevier. f) Comparison of EIS of symmetric cells processed with ultrasonic welding strategy and conventional strategy.^[^ ^218^ ^]^ Copyright 2023, Elsevier.

β‐Al_2_O_3_ was first reported for Na^+^ transport in 1967,^[^ ^209^ ^]^ which is a composite oxide of Na_2_O and Al_2_O_3_ with two crystal types, both of which are layered structures composed of stacked spinel structures. As shown in **Figure** **16**a, one is a hexagonal crystal system with the space group *P*6_3_/*mmc*, which is denoted as β‐Al_2_O_3_, and the other is a tripartite system with the space group *R*3*m*, which is denoted as β“‐Al_2_O_3_. β”‐Al_2_O_3_ contains a relatively large amount of Na^+^, and its unique structure gives it a high ionic conductivity at high temperatures.^[^ ^212^, ^213^ ^]^ At 300 °C, the ionic conductivity of single‐crystal β“‐Al_2_O_3_ reaches as high as 1 S cm^−1^, while the ionic conductivity of polycrystalline β”‐Al_2_O_3_ is 2 × 10^−3^ S cm^−1^ at room temperature and 0.2–0.4 S cm^−1^ at 300 °C.^[^ ^214^ ^]^ Although the ionic conductivity is high, the pure phase of β“‐Al_2_O_3_ is prone to reacting with water and has poor thermal stability. Therefore, during the preparation process, the β”‐Al_2_O_3_ phase is often stabilized by doping with ions such as Li⁺ and Mg^2^⁺.

The synthesis of β‐Al_2_O_3_ mainly includes solid‐phase, sol–gel, and coprecipitation methods. Studies usually aim to increase the proportion of β“‐Al_2_O_3_ to elevate the overall ionic conductivity during the synthesis. Adding crystal phase stabilizers, such as Li_2_O, MgO, TiO_2_, and MnO_2_, is effective to stabilize and thus increase the portion of the β”‐Al_2_O_3_ phase.^[^ ^215^, ^216^ ^]^ Since β“‐Al_2_O_3_ has higher conductivity at high temperature, it is easy to cause serious dendrite problems in its application. Recently, Huang et al.^[^ ^217^ ^]^ investigated the growth of sodium dendrites using β”‐Al_2_O_3_ through multi‐scale imaging and morphological dynamics tracking techniques. By in situ optical microscopy, sodium dendrites nucleate preferentially from the middle of the electrode and then expand to the side with the increase of current (Figure 16b); the deposition and stripping of the dendrites were found to follow the previously formed dendritic pathways after several cycles. In the SEM analysis of β“‐Al_2_O_3_ after the generation of dendrites (Figure 16c), it was observed to undergo through‐crystalline fracture, and the fracture surface was filled with reticulated Na metal, proving that the solid electrolyte was fractured due to the generation of sodium dendrites. From the HAADF imaging of β”‐Al_2_O_3_ before and after cycling (Figure 16d), the electrochemical cycling was found to be able to close the Na^+^ conduction planes of β“‐Al_2_O_3_. This work offers a deep understanding of the growth pathways and effects of sodium dendrites when using β”‐Al_2_O_3_ solid electrolytes, which helps address the interfacial issues of β″‐Al_2_O_3_. As for solid‐state batteries, the regulation of the interface is crucial. Dong et al.^[^ ^218^ ^]^ developed an ultrasonic welding strategy (Figure 16e) capable of quickly welding β‐Al_2_O_3_ and sodium metal together, thus decreasing the interfacial impedance (Figure 15f) to facilitate Na^+^ transport.

β“‐Al_2_O_3_ has become the mainstream electrolyte in Na–S batteries and is the only commercially available solid electrolyte currently for sodium batteries. In order to control the ratio of β/β”, many efforts have been committed to exploring new synthetic methods. However, the ionic conductivity of β″‐Al_2_O_3_, which can only be achieved at high temperatures, can no longer meet the growing demands, posing a major limitation to its further development.

##### NASICON Solid Electrolyte

**Figure 17:**
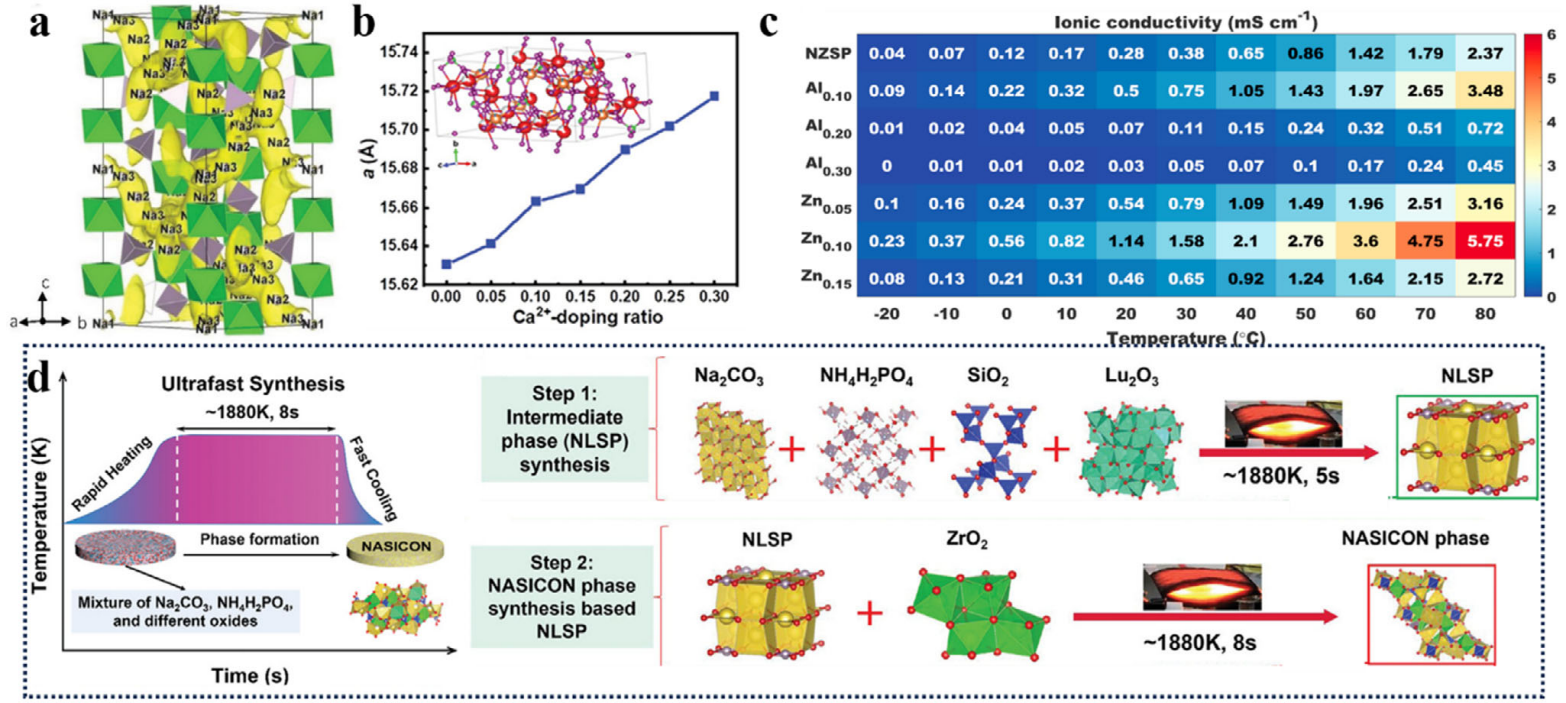
a) NASICON crystal structure at 1400 K.^[^ ^229^ ^]^ Copyright 2019, Wiley‐VCH. b) The variation of lattice parameters with Ca^2+^ doping ratio.^[^ ^221^ ^]^ Copyright 2023, Wiley‐VCH. c) The effect of different doping contents of Al and Zn on ionic conductivity at −20 to 80 °C.^[^ ^222^ ^]^ Copyright 2025, Elsevier. d) Schematic diagram of the process of ultrafast high‐temperature synthesis of NASICON solid electrolyte.^[^ ^228^ ^]^ Copyright 2023, Wiley‐VCH.

NASICON solid electrolyte is a material that allows Na^+^ to be transported in a 3D channel. Its crystal structure is shown in **Figure** **17**a, and its rate in transport of Na^+^ is faster than the 2D conduction of β‐Al_2_O_3_. This material was first reported by Goodenough et al.,^[^ ^220^ ^]^ and the general formula for NASCION type solid electrolytes is Na_1+x_Zr_2_Si_x_P_3‐x_O_12_ (0 ≤ x ≤ 3). At x = 2, Na_3_Zr_2_Si_2_PO_12_ exhibits the highest ionic conductivity, being the object of many studies. For NASICON, it has an open material framework to allow high‐valent cations to be replaced by other ions. When elements of different valence are doped into the NASICON material, both the concentration of Na^+^ and the local structure change, which eventually affect the transport channel of Na^+^, and lower the diffusion barrier to improve ionic conductivity. Therefore, researchers often modify NASICON solid electrolytes through a doping process. Wang et al.^[^ ^221^ ^]^ doped Ca^2+^into Na_3_Zr_2_Si_2_PO_12_. As the Ca^2+^ doping level increases, the lattice parameters a, b, and the monoclinic angle 𝛽 increase, while c decreases (Figure 17b). This change is induced by the larger radius of Ca^2+^ and increased concentration of Na^+^ in the lattice for charge compensation. Therefore, the prepared solid electrolyte promotes the Na^+^ transport and allows electrochemical processes at −20 °C. Moreover, Jin et al.^[^ ^222^ ^]^ synthesized Al^3+^, Zn^2+^‐doped NASICON‐type solid electrolytes using the solid phase reaction method, as shown in Figure 17c. They compared the effect of doping with different amounts of Al^3+^ and Zn^2+^ on the ionic conductivity at varied temperatures. Specifically, the ion conductivity tended to decrease with the increase of Al^3+^ doping amount, while the ion conductivity reached its highest value at a Zn doping amount of Zn_0.1_, showing a better effect than that of Al^3+^ doping. The optimized Na_3.20_Zr_1.90_Zn_0.10_Si_2_PO_12_ electrolyte exhibited a conductivity four times higher than that of undoped electrolyte. Similar doping ions include Sc^3+^, Mg^2+^, Ni^2+^, Co^2+^, etc.

Beyond compositional modification, efforts are also devoted to optimizing the synthesis method of NASICON solid electrolytes. The traditional solid‐phase method is to reduce the impedance of the electrode‐electrolyte interface by sintering at temperatures >1000 °C, which undoubtedly is energy demanding and tends to volatize Na and P, producing impurities of low ionic conductivity such as ZrO_2_. Sol–gel synthesis is a common liquid‐phase method. The raw materials are dissolved in the solvent, and the solvent is allowed for evaporation to form gel, which is subjected to high‐temperature sintering to form NASICON. Although the process is complex, the sintering temperature is lower than the solid phase method, while the conductivity of the synthesized electrolyte is usually higher.^[^ ^223^, ^224^ ^]^ In addition, other methods were reported for the preparation of NASICON solid electrolytes, such as ball milling method,^[^ ^225^ ^]^ solution‐assisted solid‐phase method,^[^ ^226^ ^]^ and discharge plasma sintering method.^[^ ^227^ ^]^ Wan et al.^[^ ^228^ ^]^ also proposed an ultrafast high‐temperature synthesis (UHS) technique to directly synthesize NASICON solid electrolytes from mixed precursor powders. The specific synthesis process is shown in the Figure 17d. Through this method, the synthesis time is shortened to a few seconds, and the synthesized electrolytes have higher ion conductivity than traditional methods.

NASICON solid electrolytes are promising because of their appropriate ionic conductivity and good electrochemical stability, pushing forward further activities in improving the conductivity. The key problem with NASICON solid electrolytes is the poor interfacial contact, which is common in solid electrolytes. Introducing several compatible modifiers at the interface can ameliorate this problem, including liquid electrolytes and polymer/sulfide solid electrolytes with low interfacial impedance. Despite such efforts, interfacial issues remain as the biggest obstacle in NASICON solid electrolytes for practical application.

##### Sulfide Solid Electrolyte

Sulfide solid electrolytes have higher conductivity and lower interfacial impedance due to the larger radius of S atom and lower electronegativity than oxygen, which reduces the interaction between S and Na^+^ to facilitate the transport of Na^+^. In the preparation of sulfide solid electrolytes, only cold‐pressing treatment is needed to have good contact with the electrodes, so it has received growing interest recently.

**Figure 18:**
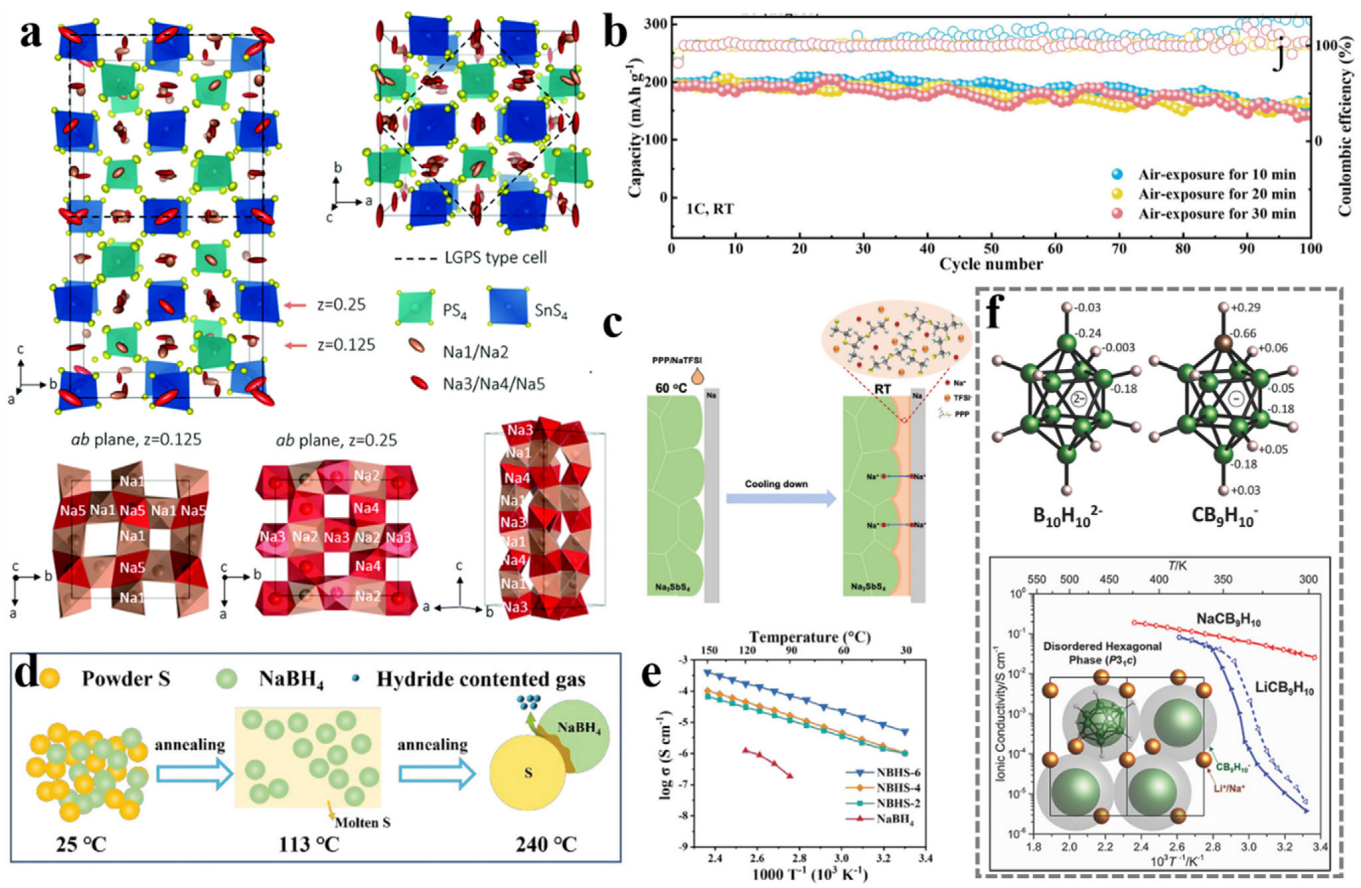
a) Crystal structure of Na_11_Sn_2_PS_12_.^[^ ^231^ ^]^ Copyright 2017, Royal Society of Chemistry. b) Cycle performance of the prepared air‐stable solid electrolyte at 50% relative humidity.^[^ ^232^ ^]^ Copyright 2024, Wiley‐VCH. c) Schematic diagram of polymer electrolyte as an intermediate layer to stabilize the interface.^[^ ^233^ ^]^ Copyright 2021, Wiley‐VCH. d) Schematic diagram of the process for synthesizing thio‐borohydride (Na‐B‐H‐S) electrolyte by dehydrogenation method.^[^ ^234^ ^]^ Copyright 2023, Wiley‐VCH. e) Variable temperature conductivity curves for different electrolytes.^[^ ^234^ ^]^ Copyright 2023, Wiley‐VCH. f) Schematic diagram of the strategy for reducing phase transition temperature by modifying anions with C.^[^ ^237^ ^]^ Copyright 2016, Wiley‐VCH.

Common sulfide solid electrolytes include Na_3_PS_4_ and Na_3_SbS_4_, both of which have two crystal structures, i.e., tetragonal phase and cubic phase, cubic phase is stable at high temperatures, while the tetragonal phase is stable at low temperatures, with the cubic phase having higher conductivity.^[^ ^230^ ^]^ Currently, the primary method for enhancing the conductivity of sulfide solid electrolytes is elemental doping. In Na_3_PS_4_, tetravalent ions such as Sn⁴⁺, Ge⁴⁺, Ti⁴⁺, and Si⁴⁺ are used to replace P⁵⁺. In addition, in 2017, Nazar et al.^[^ ^231^ ^]^ predicted a Na_11_Sn_2_PS_12_ sulfide electrolyte by ab initio molecular dynamics (AIMD), with a tetragonal crystal structure as shown in **Figure** **18**a.

Sulfide solid electrolytes are mostly unstable in humid air. They are prone to absorbing water and undergoing decomposition reactions, resulting in the production of toxic H_2_S gas. So, how to enhance the stability of sulfide electrolytes in air is a problem that needs to be addressed. Zhang et al.^[^ ^232^ ^]^ designed a sulfide solid electrolyte by coupling the vacancy effect and conformational entropy, which exhibits excellent interfacial stability to sodium metal and extraordinary tolerance to humid atmosphere and even water. Even in air at 50% relative humidity for 30 min, it can stably cycle at a rate of 1 C (Figure 18b). The challenge in sulfide electrolytes lies in the interface stability issue. To circumvent this issue, similar to NASICON, an intermediate layer can be introduced to stabilize the interface, such as phase change polymer electrolytes, as an intermediate layer to stabilize the interface, as reported by Wang et al.^[^ ^233^ ^]^ (Figure 18c). The interlayer consists of a deblock copolymer of poly(ethylene glycol)‐*block*‐poly(propylene glycol)‐*block*‐poly(ethylene glycol) (PEG‐*b*‐PPG‐*b*‐PEG) blended with sodium salt. The intermediate layer promotes the contact between the sulfide electrolyte and the electrode, enabling uniform deposition of Na^+^.

The research on sulfide electrolytes for SIBs is in its infancy stage, and the development of advanced electrolytes is relatively scarce. Sulfide solid electrolyte has high ionic conductivity, while its stability in air and its own chemical stability need to be further improved.

##### Compound Hydride Solid Electrolyte

There are other solid electrolytes in SIBs, e.g., compound hydride solid electrolytes that have been widely researched and focused on sodium borohydride and its derivatives. Song et al.^[^ ^234^ ^]^ presented a synthetic method towards novel thio‐borohydride electrolytes by a one‐step dehydrogenation reaction (Figure 18d). In this method, thio‐borohydride was in situ generated via a chemical reaction between two low‐cost feedstocks of NaBH_4_ and S. The reaction temperature could be drastically reduced to 240 °C with the help of partial dehydrogenation of NaBH_4_. The ionic conductivity of the synthesized Na‐B‐H‐S (NaBH_4_/Na‐B‐S) electrolyte was boosted by three orders of magnitude compared to the pristine NaBH_4_ (Figure 18e). Via this strategy, not only, the cost and energy consumption reduced, but also the migration of Na^+^ was increased. Compared to small anions, compound hydrides containing large anions (*e.g*., B_12_H_12_^−^, B_10_H_10_^−^) exhibit higher ionic conductivity above the order‐disorder structural phase transition temperature.^[^ ^235^, ^236^ ^]^ However, the phase transition temperature of compound hydrides is too high. To meet the actual needs, the methods of anionic chemical modification, anionic mixing, and grain nano‐crystallization/disorder are often used to reduce the phase transition temperature, e.g., introducing C for anion modification reduces the phase transition temperature of NaCB_11_H_12_ to 380 K (Figure 18f).^[^ ^237^ ^]^ Additionally, future research should consider the electrochemical window of compound hydrides to match electrode materials at higher operating voltages.

##### Halide Solid Electrolyte

In recent years, emerging halide solid electrolytes have shown good oxidative stability and good compatibility with cathodes.^[^ ^238^, ^239^ ^]^ Meanwhile, the weak bonding of Na^+^ with halide ions and the high polarizability of halide anions make halide electrolytes possess excellent mechanical deformability. All these features position halide electrolytes as one of the most promising solid electrolytes.

Since the ionic conductivity of lithium halides was reported in the 1930s, a number of halide electrolytes containing metallic elements have been prepared and investigated.^[^ ^240^, ^241^ ^]^ However, due to the limited ionic conductivity at room temperature, research on halide electrolytes was largely overlooked until 2018, following the breakthrough discovery of Li_3_YCl_6_ and Li_3_YBr_6_ by Asano et al.^[^ ^238^ ^]^ Compared to LIB, the development of sodium‐based halide electrolytes has lagged behind, and a common halide electrolyte can be denoted as Na_a_MX_b_, where M is the metal element, X for halide, and ab varies according to the valence state of M. The metallic elements mainly include the third and fourth subgroup elements (Sc, Y, La‐Lu, Zr, Hf, etc.), the fifth subgroup (Nb, Ta, etc.) and the third main group (Al, Ga, In, etc.).

**Figure 19:**
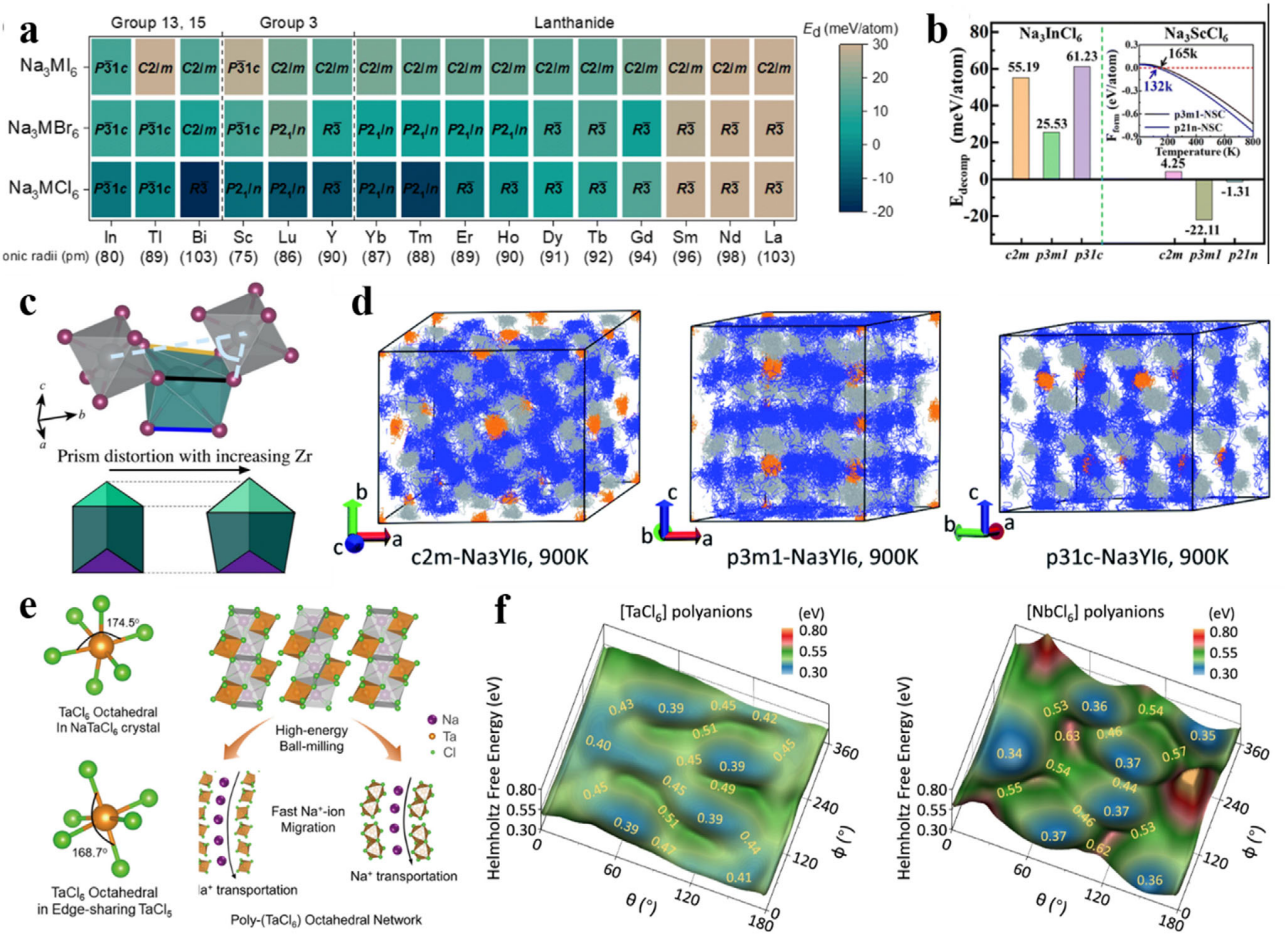
a) The most stable structural phase and decomposition energy of Na_3_MX_6_.^[^ ^243^ ^]^ Copyright 2022, Royal Society of Chemistry. b) Decomposition energies of Na_3_InCl_6_ and Na_3_ScCl_6_ with different structures.^[^ ^244^ ^]^ Copyright 2022, Royal Society of Chemistry. c) Schematic diagram of structural transformation with increasing Zr.^[^ ^245^ ^]^ Copyright 2020, American Chemical Society. d) Transport behavior of sodium ions in Na_3_YI_6_ with different structures simulated by AIMD.^[^ ^248^ ^]^ Copyright 2021, Royal Society of Chemistry. e) Transport mechanism of Na^+^ in NaTaCl_6_ halide solid electrolyte.^[^ ^251^ ^]^ Copyright 2024, Elsevier. f) Helmholtz free energy surface of NaTaCl_6_ and NaNbCl_6_ at 600K.^[^ ^252^ ^]^ Copyright 2025, Springer Nature.

Na_3_MX_6_ has various crystal structures, including the trigonal $\bar{P3}$1c, rhombic $\bar{R3}$ phase, and monoclinic *P*2_1_/n and *C*2/m structures.^[^ ^242^ ^]^ The most stable structure and the decomposition energy of each Na_3_MX_6_ are summarized in **Figure** **19**a. Chung et al.^[^ ^243^ ^]^ investigated the effect of different halides on the structure of electrolytes and found that Na_3_MCl_6_ and Na_3_MBr_6_ tended to form the $\bar{P3}$ *1c*, *P2_1_/n*, and $\bar{R3}$ phases, while Na_3_MI_6_ tended to form the *C2/m* phase. Among them, the *C2/m* phase of Na_3_MI_6_ exhibited a high ionic conductivity of about 10^−4^ S cm^−1^. The conductivity can be further enhanced to about 10^−3^ S cm^−1^ by combining Br to form Na_3_MBr_3_I_3_. The most researched of Na_3_MX_6_ is Na_3_MCl_6_ (M = In, Sc, Er, Y, etc.), where the type of M and the ionic radius determine the stable structure of the electrolyte. Ouyang et al.^[^ ^244^ ^]^ evaluated the stability, mechanical properties, and sodium ion diffusion mechanisms of Na_3_InCl_6_ and Na_3_ScCl_6_ with different structures by theoretical calculations. Compared with the *P31c* structure, the *C2/m* and *P3m1* structures have better stability and wider electrochemical windows (Figure 19b). The electrolyte with the *P3m1* structure has the highest ionic conductivity. Na_3_ErCl_6_ is a monoclinic structure with the space group *P2_1_/n*. Zeier et al.^[^ ^245^ ^]^ synthesized Na_3‐x_Er_1‐x_Zr_x_Cl_6_ by using Zr^4+^ to replace Er^3+^. The introduction of Zr increased the vacancy concentration in the crystal to boost the ionic conductivity. Due to the much smaller ionic radius of Zr^4+^, the crystal cells contracted and the structure was gradually transformed into a tetragonal one as the Zr content increased (Figure 19c). In addition, yttrium‐based sodium halides exhibit significant advantages in terms of ionic conductivity and electrochemical stability.^[^ ^246^ ^]^ Na_3_YCl_6_ is usually monoclinic with a space group of *P2_1_/n*, but different synthesis methods affect its crystal structure, with some quenching or ball milling being able to obtain the *P2_1_/n* phase, while slow cooling forms the thermodynamically stable $\bar{R3}$ phase.^[^ ^247^ ^]^ Wang et al.^[^ ^248^ ^]^ investigated the transport behavior of sodium ions in different structures of Na_3_YI_6_ (Figure 19d), Na^+^ diffusion in the *C2/m* and *P3m1* structures follows a 3D cross‐linked network, whereas in $\bar{P3}$ *1c*‐Na_3_YI_6_ it diffuses along the axial 1D channels. This explains the low ionic conductivity of the $\bar{P3}$ *1c*‐Na_3_YI_6_ electrolyte.

Jung et al.^[^ ^249^ ^]^ prepared NaAlCl_4_ by ball milling and applied it to all‐solid‐state SIBs, which showed tenfold enhancement in ionic conductivity compared to high‐temperature annealed samples, NaCrO_2_||Na_3_Sn cells using this electrolyte exhibited excellent cycling performance at both 30 and 60 °C. NaAlCl_4_ is an electrolyte with significant advantages in cost, but its ionic conductivity remains low, which can be improved by ionic doping. For instance, Tang et al. achieved an ionic conductivity of 3.56 × 10^−5^ S cm^−1^ at room temperature for a sample by F doping. Furthermore, Manthiram et al.^[^ ^250^ ^]^ prepared O‐doped NaAlCl_4‐2x_O_x_ electrolyte using Na_2_O as the oxygen source, and measured the variation of its ionic conductivity with x value. Via investigation, the essential reason for the enhancement of the ionic conductivity is the in situ generation of Al_2_O_3_ nanoparticles inside the electrolyte.

In 2024, Sun et al.^[^ ^251^ ^]^ developed an amorphous NaTaCl_6_ halide solid electrolyte with an ultra‐high ionic conductivity of 4 × 10^−3^ S cm^−1^ at room temperature. The conduction mechanism of Na^+^ is shown in Figure 19e. The TaCl_6_^−^ octahedra anions are close to each other and undergo significant deformation to form a poly(TaCl_6_) octahedral network, the Na^+^ and Cl^−^ interaction is weakened due to the electrostatic force, which facilitates the migration and conduction of Na^+^. This study successfully combines Na halide electrolyte with poly anionic Na_3_V_2_(PO_4_)_3_ cathode for the first time and achieves excellent performance. Recently, Hu et al.^[^ ^252^ ^]^ also prepared a NaTaCl_6_ electrolyte, which exhibited a high ionic conductivity of 3.3 mS cm^−1^ at 27 °C, two orders of magnitude higher than that of NaNbCl_6_. The authors proposed a “paddle wheel mechanism” to explain why the NaTaCl_6_ electrolyte has a higher ionic conductivity: compared to [NbCl_6_], the rotation of [TaCl_6_] polyanions is easier to achieve at room temperature. Additionally, NaTaCl_6_ exhibits higher structural disorder, further promoting the significant rotation of [TaCl_6_] anions. Figure 19f shows the Helmholtz free energy surface of the two electrolytes at 600 K. It can be seen that the Cl ligand bound to Ta atoms exhibits a shallower and flatter energy landscape, indicating that the Cl ligand in NaTaCl_6_ has a lower rotational potential barrier. The rotation of polyanions temporarily expands the bottleneck of Na^+^ ion migration and causes significant fluctuations in the local configuration, thereby reducing the diffusion energy barrier of Na^+^ and increasing the migration entropy. This work reveals the influence of anions on Na^+^ diffusion behavior, which is conducive to further development of solid electrolytes with better performance.

#### 3.5.2 Polymer Solid Electrolytes

**Figure 20:**
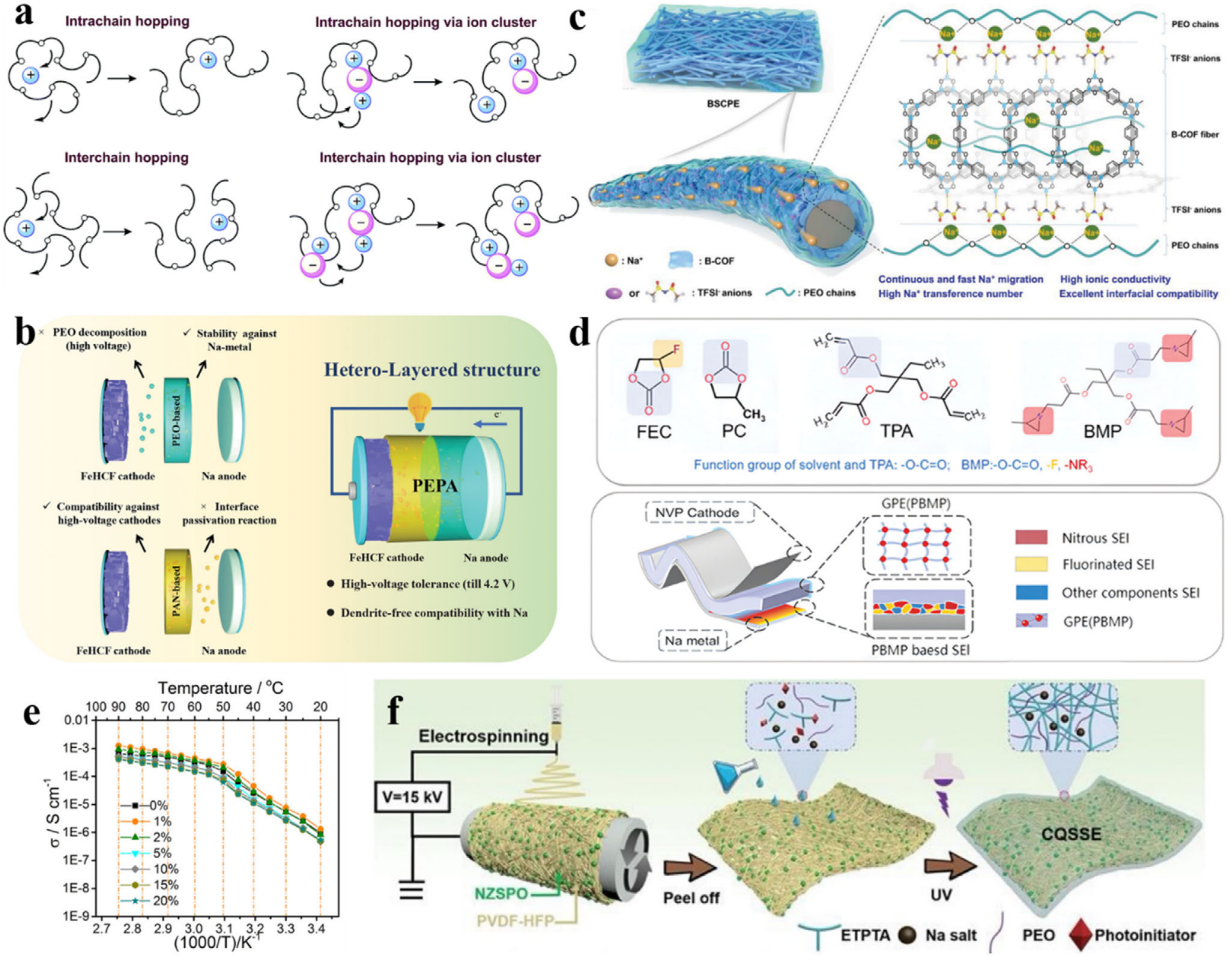
a) Mechanism of ion transport in PEO.^[^ ^254^ ^]^ Copyright 2015, Royal Society of Chemistry. b) Schematic diagram of PEO and PAN composite as electrolyte application.^[^ ^255^ ^]^ Copyright 2023, Wiley‐VCH. c) Schematic diagram of B‐COF promoting Na^+^ transport in polymer electrolytes.^[^ ^256^ ^]^ Copyright 2024, Wiley‐VCH. d) Schematic diagram of the molecular structure of solvents in polymer electrolytes and their protection against Na metal.^[^ ^257^ ^]^ Copyright 2024, Wiley‐VCH. e) Ionic conductivity of electrolytes various Al_2_O_3_ addition contents.^[^ ^261^ ^]^ Copyright 2019, American Chemical Society. f) Schematic diagram of the preparation of hybrid electrolytes.^[^ ^262^ ^]^ Copyright 2023, Wiley‐VCH.

Polymer solid electrolytes are a class of complexes formed by dissolving Na salts in a polymer matrix. Compared to inorganic solid electrolytes, polymer solid electrolytes have a lower ionic conductivity but better flexibility and closer contact with the electrode.^[^ ^253^ ^]^ Common polymer systems include PEO, PMMA, polyvinylidene fluoride‐hexafluoropropylene (PVDF‐HFP) and polyacrylonitrile (PAN). Among them, PEO is the most used system, which has good solubility for sodium salts and solvation with Na^+^ through oxygen in the chain segments. In the presence of an electric field, the polymer molecular chain segments can move, and cations or ion clusters continuously coordinate and dissociate with the functional groups on the polymer chain, hopping from one coordination site to the next new coordination site, thereby achieving ion transfer. This type of transport is known as hopping transport (**Figure** **20**a).^[^ ^254^ ^]^ The problem with PEO‐based polymer solid electrolytes is that they are commonly crystalline at room temperature, causing low ion motion, so researchers often modify them to address this problem. When PEO is blended with other polymers, it is possible to effectively combine the advantages of each. For example, Ma et al.^[^ ^255^ ^]^ compounded PEO with PAN, effectively utilizing the stability of PEO for the anode and PAN for the high voltage cathode (Figure 20b). Composite electrolytes have better comprehensive performance. Of course, it is also possible to modulate the PEO structure itself. For example, Liu et al.^[^ ^256^ ^]^ distributed a boron‐containing covalent organic framework (B‐COF) network with anion trapping function in PEO (Figure 20c). The abundant Lewis acid sites on the B‐COF network promote the dissociation of sodium salts, and a high Na^+^ migration number of 0.71 can be achieved due to anion trapping. The ionic conductivity can reach 5.28 × 10^−4^ S cm^−1^ at 60 °C by this method.

Although PEO is currently considered a default polymer electrolyte with excellent comprehensive performance, various new polymer solid electrolyte systems are being explored. Zhao et al.^[^ ^257^ ^]^ synthesized two polymers with 3D network structures, i.e., poly‐bis(3‐(2‐methylaziridin‐1‐yl) propanoate) (PBMP) and poly(trimethylolpropane triacrylate) (PTPA). The molecular structure is shown in Figure 20d. Compared to PTPA, PBMP with N‐containing functional groups is more inclined to react with metal Na to form N‐containing SEI, which promotes Na^+^ transport and hence better cycling performance. Moreover, both of the developed polymer solid electrolytes perform better than the common carbonate liquid electrolyte.

Regardless of the type of solid electrolytes, the improvement of battery safety is obvious, while two challenges still exist in solid electrolytes, i.e., the ion conductivity to be improved and the interface contact to be optimized. There are existing methods to improve ionic conductivity, but how to provide a comparable ionic transport rate with a liquid electrolyte at room temperature is the next direction to work on. Currently, solid‐state sodium batteries typically use sodium metal as the anode, partly due to the higher energy density it offers, and partly because poor interfacial compatibility makes it challenging to integrate other anode materials with solid electrolytes. However, owing to the limitation of active Na metal, solid‐state sodium batteries need to be prepared in a special gaseous environment, which undoubtedly increases the operational difficulty and cost. Therefore, developing new anodes and improving the compatibility of solid electrolytes is crucial for further practical application.

#### 3.5.3 Hybrid Electrolytes

As mentioned earlier, inorganic solid electrolytes have higher ionic conductivity, while polymer solid electrolytes have good film‐forming properties and high toughness. A single material is unable to simultaneously meet the various requirements of high‐energy‐density solid‐state batteries, whereas mixing multiple materials can synergistically integrate the advantages of each component to enhance overall performance. Inorganic‐organic polymer hybrid solid electrolytes are typically composed of organic polymeric macromolecules, sodium salts, and inorganic fillers. The fillers are primarily divided into two categories: The first is the inert filler that lacks ion‐conducting capabilities, such as Al_2_O_3_, SiO_2_, MgO, and TiO_2_. These inert fillers can act as cross‐linking centers to hinder the recrystallization of polymer segments, thereby reducing the polymer's crystallinity. Additionally, the functional groups on the surface of inert fillers can interact with sodium salts, promoting their dissociation.^[^ ^183^, ^258^ ^]^ The second is the active filler that can transport ions itself, which can provide additional ion transport pathways, thereby further improving the ion conductivity of the electrolyte.^[^ ^259^ ^]^

Hwang et al.^[^ ^260^ ^]^ combined TiO_2_ with NaClO_4_/PEO. When 5 *wt*% TiO_2_ was added, the ionic conductivity increased to 2.62 × 10^−4^ S cm^−1^ (60 °C). This is because the addition of TiO_2_ increased the amorphous region in PEO, which facilitated the creep of PEO segments and accelerated Na⁺ transport. Moreover, Hu et al.^[^ ^261^ ^]^ added Al_2_O_3_ to NaFSI/PEO and found that when the addition was less than 2 wt%, the ionic conductivity improved, while it decreased at the content beyond 5 wt% (Figure 20e). This indicates that for inert fillers, the addition amount must be strictly optimized through experimentation to determine the optimal value.

Active fillers are typically inorganic ion conductors. For example, Jiao et al.^[^ ^262^ ^]^ used electrospinning technology to uniformly disperse Na_3_Zr_2_Si_2_PO_12_ (NZSPO) on PVDF‐HFP fibers and introduced an ethoxylated trimethylolpropane triacrylate (ETPTA) precursor solution to serve as a fast ion channel (Figure 20f). This method enables the hybrid electrolyte to achieve an ultra‐high ionic conductivity of 4.1 mS cm^−1^ at room temperature. Due to the preferential reducibility of PVDF‐HFP, a stable interface rich in NaF is formed, enabling the NVP||Na cell to cycle stably for 400 times. In addition, Yao et al.^[^ ^263^ ^]^ prepared a hybrid solid electrolyte by solution casting method using Na_3.4_Zr_1.9_Zn_0.1_Si_2.2_P_0.8_O_12_ mixed with PEO and [Py_13_][NTf_2_], with a NASICON type electrolyte content of 80%.This electrolyte not only exhibits high ionic conductivity at room temperature (1.48 × 10^−4^ S cm^−1^), but also has overall flame retardancy due to the introduction of IL, making the battery highly safe.

Although inorganic–organic polymer hybrid solid electrolytes have attracted attention, research is still in its exploratory stage, and multi‐performance synergistic optimization faces systemic challenges. Specifically, these challenges include: 1) dual constraints of ionic conductivity and interfacial dynamics; 2) trade‐offs between mechanical strength and ionic transport; 3) agglomeration issues during the preparation of nanofillers; and 4) electrochemical windows that are still difficult to meet the application requirements of high‐voltage materials.

#### 3.5.4 Solid Electrolyte Interface

The contact, ion transport, and stability of interfaces in solid electrolytes are crucial for improving the comprehensive performance of batteries. The interfaces in solid electrolytes mainly consist of three parts, namely the one between the electrolyte and the electrode, the one between the grains within the electrolyte, and the one between the conductive agent and the active material of the electrode.^[^ ^264^ ^]^ Liquid electrolytes have intrinsically high fluidity and good wettability with electrode materials, ensuring low contact resistance. However, in solid‐state batteries, the electrodes and electrolytes are in solid‐solid contact, resulting in a small contact area due to surface roughness and other conditions, and correspondingly a high interface resistance. Currently, interface modification work for solid‐state batteries mainly focuses on improving the contact between the electrolyte and the electrode to reduce impedance. Different types of solid electrolytes have different interface contacts. Among them, inorganic solid electrolytes probably have the smallest contact area with the electrode due to their rigid structure. Therefore, modification efforts are primarily concentrated on inorganic solid electrolytes. In this section, we provide a summary of various interface modification strategies.

##### Electrode Material Modification Engineering

**Figure 21:**
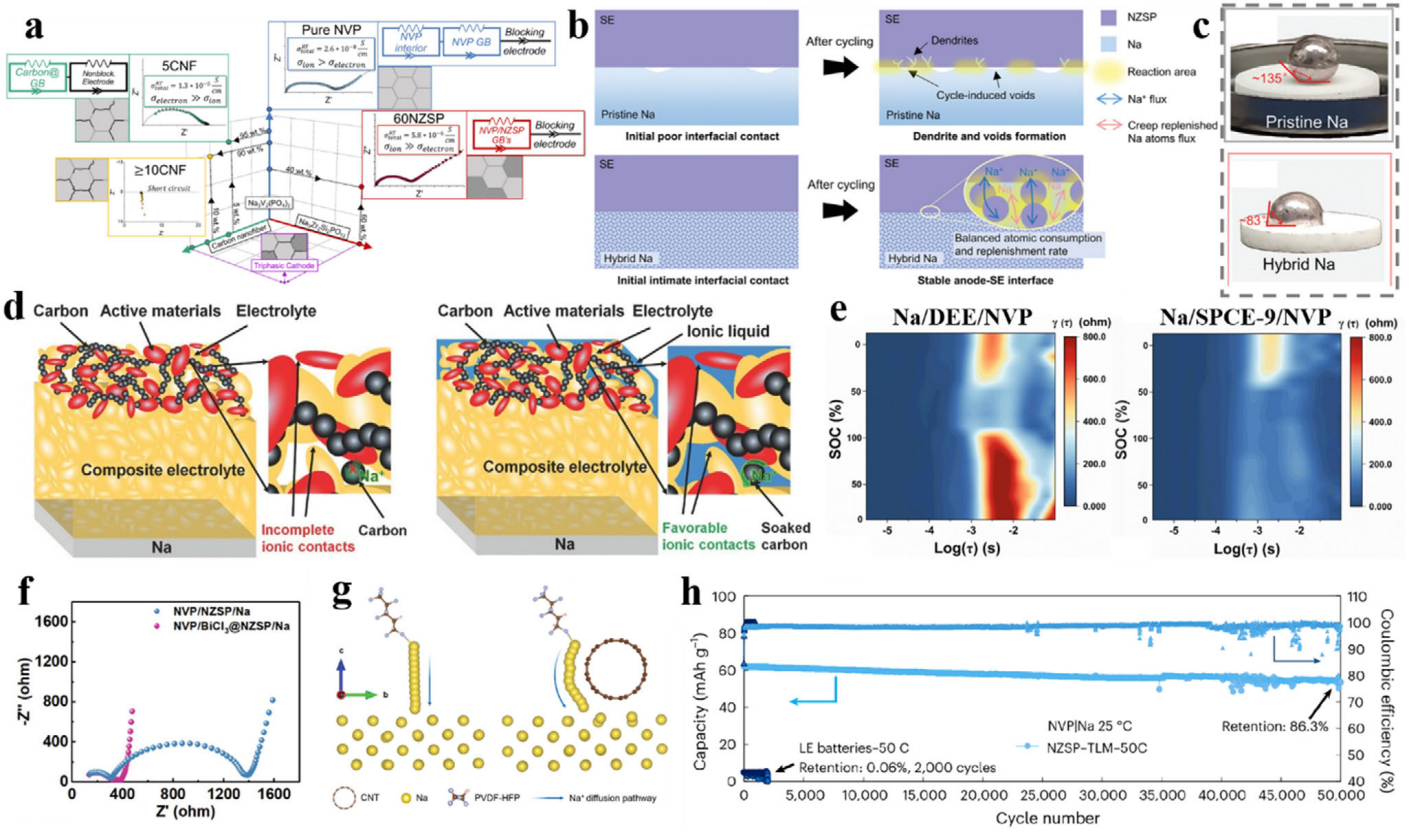
a) Schematic diagram of the structural modification effect of cold sintered hybrid cathode.^[^ ^266^ ^]^ Copyright 2021, American Chemical Society. b) Schematic diagram of interface modification by hybrid Na anode.^[^ ^268^ ^]^ Copyright 2021, Wiley‐VCH. c) Comparison of the wettability of different Na on NZSP.^[^ ^268^ ^]^ Copyright 2021, Wiley‐VCH. d) Schematic diagram of the effect of IL addition on interfacial ion transport.^[^ ^270^ ^]^ Copyright 2016, Wiley‐VCH. e) In situ EIS using DEE and SCPE‐9 electrolytes.^[^ ^271^ ^]^ Copyright 2025, Wiley‐VCH. f) EIS curves of NVP/BiCl_3_@NZSP/Na and NVP/NZSP/Na full cells.^[^ ^273^ ^]^ Copyright 2024, Wiley‐VCH. g) Different diffusion pathways of Na^+^.^[^ ^274^ ^]^ Copyright 2024, Wiley‐VCH. h) Long cycle performance of NVP||Na full cell at 50 C.^[^ ^275^ ^]^ Copyright 2025, Springer Nature.

Porous and rigid cathode materials result in poor interface contact and usually have high resistance. Contact problems are not only found at the electrode/electrolyte interface, but also inside the electrode. The composite of cathode active materials and electrolytes (e.g., polymers and sulfides) can promote ion transport and has good toughness, thereby reducing interface resistance. Wang et al.^[^ ^265^ ^]^ prepared a mesoporous carbon‐based Na_2_S‐Na_3_PS_4_‐CMK‐3 composite cathode through a melt‐casting‐annealing‐precipitation process, achieving close contact between the cathode material, electrolyte, and conductive additive. In addition, Randall et al.^[^ ^266^ ^]^ employed a cold sintering method to mix NVP cathode material, Na_3_Zr_2_Si_2_PO_12_ (NZSP) electrolyte, and conductive carbon nanofibers (CNP), effectively increasing interface contact, thereby enhancing ionic conductivity and reducing interface impedance (**Figure** **21**a).

For anode materials, composite anodes and alloy anodes are often used to improve interfacial ion transport and enhance interfacial stability. For example, Luo et al.^[^ ^267^ ^]^ prepared a Na–SiO_2_ composite anode by combining amorphous SiO_2_ with metallic sodium. When using NASICON electrolyte, it has excellent wettability and stability, and the battery impedance was reduced drastically from 1658 to 101 Ω cm^−2^. Jiang et al.^[^ ^268^ ^]^ prepared a hybrid anode by immersing Na_3.4_Zr_2_Si_2.4_P_0.6_O_12_ (NZSP) powder in molten Na. This method increased the contact area between the anode and the electrolyte and improved wettability, with the contact angle dropping from 135° to 83° (Figure 21b,c). To note, some alloy anodes are often used to improve ion transport at the solid electrolyte interface.

Nanostructuration of electrode materials is also an effective method to improve interfacial contact. For example, Wang et al.^[^ ^269^ ^]^ used Na_3_PS_4_–Na_2_S‐C nano‐composite materials as the cathode. Compared to micron‐scale Na_3_PS_4_, nano‐scale Na_2_S can ensure sufficient interface contact, thereby reducing battery impedance.

##### Solid–Liquid Mixture Engineering

Adding some interface wetting agents, such as liquid electrolytes, to transform the “point” contact between solid particles into “surface” contact is an effective method for reducing interface impedance. For example, Huang et al.^[^ ^270^ ^]^ introduced PP_13_FSI‐IL to enhance interfacial wetting. Due to the non‐flammability of ILs, the introduction of ILs does not affect battery safety. Meanwhile, it enables more intimate contact between the active material and the electrolyte (Figure 21d), thereby increasing the Na^+^ transport rate and reducing interfacial resistance. In addition to ILs, some non‐flammable plasticizers are used as well to enhance the conductivity of the electrolyte. Hu et al.^[^ ^271^ ^]^ mixed succinimide (SN) with a polymer electrolyte to construct a solid‐state plastic crystal electrolyte (SPCE‐9). The polymer segments enhance the dissociation of Na salts, while Na^+^ rapidly migrates through the electrolyte via interactions with SN. This efficient Na^+^ migration pathway enables the battery to achieve excellent kinetic rates. As shown by in situ EIS characterization (Figure 21e), the interfacial impedance in SPCE‐9 is lower than that in the electrolyte without SN at different SOC.

##### Artificial Interface Layer

Since solid electrolytes undergo chemical or electrochemical reactions with electrodes to form an intermediate layer, the properties of the intermediate layer have a significant impact on interface impedance. Therefore, it is necessary to optimize the intermediate layer to reduce interface impedance. Some flexible polymer intermediate layers are often used to alleviate interface contact, such as PEO and PAN‐based polymers.^[^ ^214^, ^272^ ^]^ What's more, there are some composite interface layers. Fan et al.^[^ ^273^ ^]^ used spin coating to build an organic–inorganic composite BiCl_3_/PTFE flexible interface layer on the NZSP surface. During cycling tests, BiCl_3_ reacts with sodium to form a multifunctional interface layer composed of Na_x_Bi and NaCl. Na_x_Bi has a low ionic diffusion energy barrier, promoting Na^+^ transport, while NaCl hinders electron injection. Using this modified electrolyte to assemble an NVP||Na full cell, the impedance decreased from 1383.6 to 383.4 Ω (Figure 21f) and inhibited Na dendrite growth.

During sodium deposition, the morphology and rate of deposition are highly dependent on the concentration of ions at the interface and the electric field. To promote Na nucleation, Liang et al.^[^ ^274^ ^]^ used oxidized carbon nanotubes (@CNT) to form a mixed‐ion/electron‐conducting hybrid solid electrolyte (MIECHSE) to regulate interfacial built‐in electric field (IBEF). Specifically, the MIECHSE altered the electric field at the interface, thereby influencing the Na^+^ diffusion path (Figure 21g). The intermediate layer, on the one hand, homogenizes the electric field distribution at the interface, reducing the Na^+^ diffusion energy barrier; on the other hand, it eliminates local charge aggregation and tip effects on the electrode surface, promoting uniform sodium deposition. This interface with a regulated electric field also reduces interface resistance, enhancing the battery's cycling stability. Recently, Wang et al.^[^ ^275^ ^]^ pointed out that when solid electrolytes are mixed with liquid electrolytes, there is a problem of kinetic imbalance, resulting in different Na^+^ transport rates. This leads to the formation of Na vacancies on the surface of the solid electrolyte, causing the generation of local electric fields, which accelerate the decomposition of organic solvents and affect battery performance. To address this issue, their research team designed an ion‐anchored intermediate layer (PETEA, i.e., pentameric tetrapropylene acrylate). Through screening, PETEA was found to possess moderate solvation capacity and low polarity, enabling rapid Na^+^ migration at the interface, reducing impedance, and avoiding the issue of kinetic imbalance. The NVP||Na full cell using this artificial interface can achieve 50 000 ultra‐long cycles at a high rate of 50 C (Figure 21h). This work provides a new direction for high‐performance solid‐state SIBs.

The interface resistance and compatibility issues of solid electrolytes have long been the bottleneck limiting the development of all‐solid‐state batteries. These interface defects not only significantly reduce the battery's room‐temperature ionic conductivity and rate performance, but also easily trigger harmful side reactions, contact failure, or even sodium dendrite growth, soundly impairing the battery's cycle life and safety. Therefore, overcoming this bottleneck is crucial for the realization of future high‐performance all‐solid‐state SIBs. Future research is encouraged to investigate the complex physicochemical processes at solid‐solid interfaces, developing models for interfacial ion transport based on these findings, and exploring novel interfacial modification strategies to achieve a significant reduction in interfacial resistance, thereby paving the way for the practical application of all‐solid‐state SIBs.

## 4 Interfacial Chemistry of SIBs

The concept of the SEI has been mentioned in previous sections. The SEI film has been intensively studied in batteries, as it can protect electrode materials and prevent the continuous decomposition of electrolyte during cycling. SEI begins to form during the first cycle. Because SEI also plays a role in the transport of ions, the structure, properties, and stability of the SEI are relevant in terms of operational stability for a long time. The formation of SEI mainly comes from the decomposition and oxidation of the electrolyte on the electrode surface, and the SEI in SIBs is more easily dissolved than that in LIBs.^[^ ^21^, ^276^ ^]^ As such, there is a greater obstacle to the design of SEI film in SIBs. In this section, we elaborate on the formation and aging process of SEI, and summarize the characteristics and influencing factors of an ideal SEI. Furthermore, we have noticed the emerging concept of “SEI free” battery, which usually occurs in the co intercalation phenomenon of ether electrolytes. Due to the existence of the co‐intercalation mechanism, some researchers debatably believe that there is no formation of SEI. Therefore, we include all these aspects below and provide our own insights.

### 4.1 Formation and Aging of SEI

#### 4.1.1 SEI Formation

**Figure 22:**
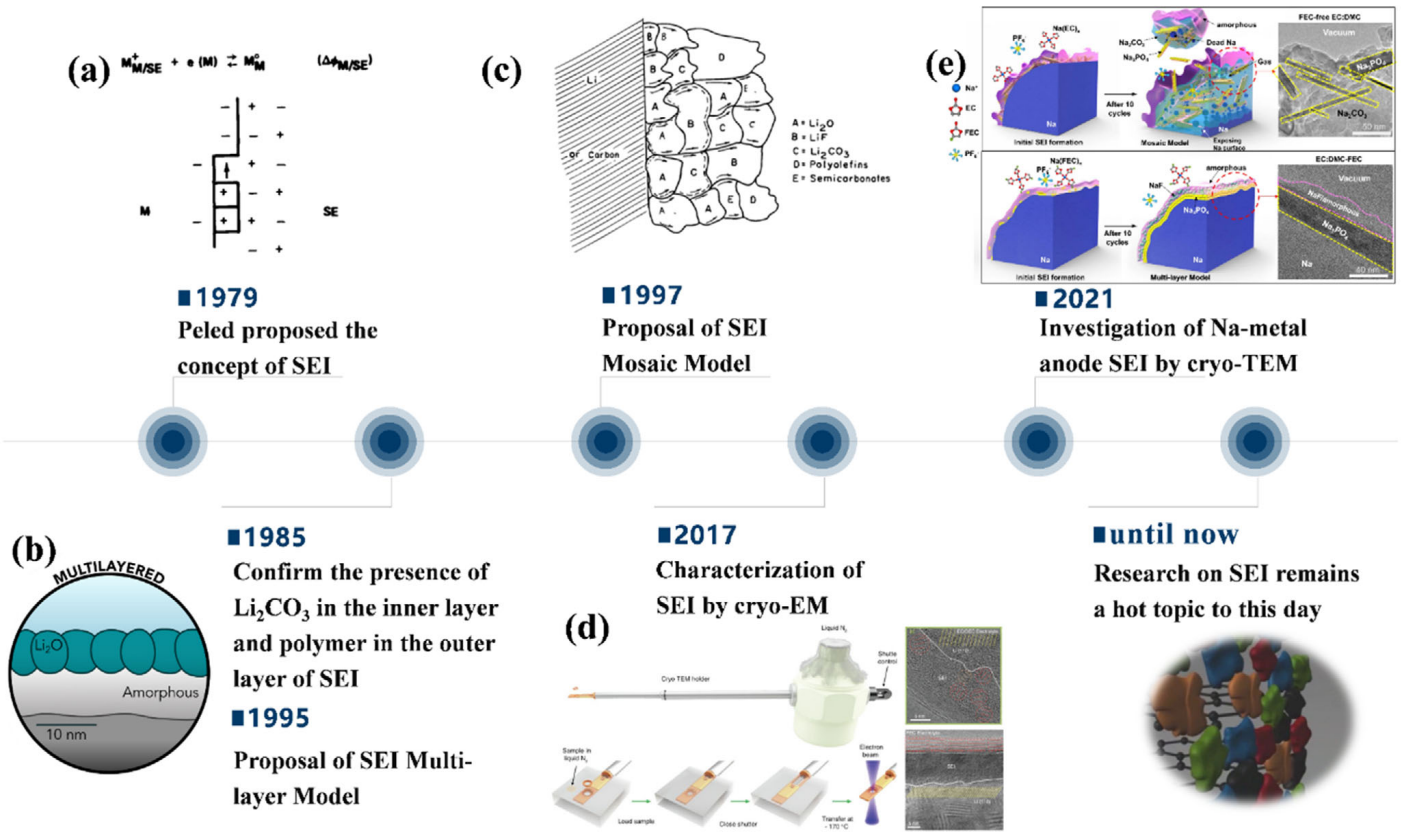
Partial research progress timeline of SEI. a) The concept of SEI was first proposed in 1979.^[^ ^277^ ^]^ Copyright 1979, The Electrochemical Society. b) Schematic diagram of SEI multilayer model.^[^ ^289^ ^]^ Copyright 2023, Elsevier. c) Schematic diagram of SEI mosaic model.^[^ ^283^ ^]^ Copyright 1997, The Electrochemical Society. d) SEI morphology measured by cryo‐EM.^[^ ^287^ ^]^ Copyright 2017, The American Association for the Advancement of Science. e) Characterization of SEI of Na‐metal by cryo‐TEM.^[^ ^288^ ^]^ Copyright 2021, Springer Nature.

Since Peled introduced the concept of SEI in 1979 (**Figure** **22**a),^[^ ^277^ ^]^ researchers have intensively explored its formation mechanism. Generally, it is believed that the formation process of SEI film runs as follow: during the first cycle of the battery, the electrolyte decomposes to produce a series of organic and inorganic substances, which accumulate on the electrode surface into a passivation film.^[^ ^278^ ^]^ The decomposition of the electrolyte is due to the fact that its LUMO energy level is lower than the Fermi level of the anode, so the electron flows from the anode to the LUMO to initiate the reduction reaction. The LUMO level here refers not to a single solvent or sodium salt, but to the overall solvation structure. Although some literatures use the LUMO level of a single solvent to compare the reduction ability, this only represents a tendency. A more accurate method should compare the reduction ability of the entire solvation structure.^[^ ^279^, ^280^ ^]^ The SEI formed after the first cycle is thin and incomplete, and cannot continuously resist electron transfer. Therefore, the electrolyte continues to decompose, leading to secondary growth of the SEI film until complete electrical insulation is achieved.^[^ ^281^, ^282^ ^]^ In summary, the SEI film grows during the early stage of battery cycling, which determines the composition, thickness, mechanical strength, and other properties of the final stable SEI. A stable SEI can not only protect the electrode material, but also improve the reaction kinetics at the interface. A deep understanding of the formation process of SEI is helpful to fundamentally design and construct a stable interface.

After proposing the concept, researchers aspire to describe the structure of the SEI through models, the most typical of which are mosaic model^[^ ^283^ ^]^ and multilayer model^[^ ^284^ ^]^ (Figure 22b,c). With further study, the multilayer structure of SEI is thought to be divided into two main layers: the inner layer near the anode is inorganic, and the outer layer is made up of organic compounds.^[^ ^285^ ^]^ It was confirmed by in situ XRD as early as 1985 by Muller et al.^[^ ^286^ ^]^ who found Li_2_CO_3_ in the inner layer of SEI and the presence of polymers in the outer layer. In recent years, the rapid development of advanced characterization techniques has led to new insights into the structure of SEI. For example, Cui et al.^[^ ^287^ ^]^ observed the SEI structure on the surface of lithium metal through cryogenic electron microscopy (cryo‐EM) (Figure 22d). When the FEC additive was introduced, it was able to transform the SEI structure from a pristine mosaic structure to an ordered multilayer structure; it was concluded that the ordered multilayer SEI could provide higher mechanical durability. The application of this new characterization tool facilitates further observation of the SEI structure. Under the same conditions, Gu et al.^[^ ^288^ ^]^ investigated the SEI of Na metal (Figure 22e) and showed that FEC was able to make the SEI thinner and more homogeneous.

Beside the structural modelling of SEI, several concepts were proposed to explain the formation mechanism of SEI. As early as 1853, Helmholtz proposed the electrical double layer (EDL) model to explain the charge distribution between the surface of the electrode and the electrolyte. Then, Gouy and Chapman revised the model to comply with higher electrolyte concentrations, Stern combined the Helmholtz and Gouy–Chapman models and proposed the inner/outer Helmholtz plane.^[^ ^290^, ^291^ ^]^ In the inner layer, some solvent molecules and anions adsorb on the electrode surface and preferentially participate in decomposition. The EDL structure plays a crucial role in the formation of SEI.^[^ ^292^ ^]^ Later, the Bockris–Devanathan–Mϋller model took into account interfacial solvent depolarization and suggested that solvent molecules close to the electrode interface played a decisive role.^[^ ^293^ ^]^ Although to date, the EDL theory is still the most widely used in the field of electrochemistry, these aforementioned other theories have helped us with a deeper understanding of the formation and growth of interfaces.

#### 4.1.2 SEI Aging

After the formation of SEI, the battery can exhibit ideal cycle stability if it remains stable. However, the battery capacity will decay after a certain period, which is mainly due to the aging of SEI. The aging of SEI is affected by various factors, such as the continuous decomposition of electrolyte, dissolution/reconstruction of SEI, the production of hazardous substances, and the influence of electrode materials. In parallel to these internal factors, harsh external environments (high/low temperatures), fast charging, and high voltage can accelerate SEI aging, making SEI aging a complex process.

**Figure 23:**
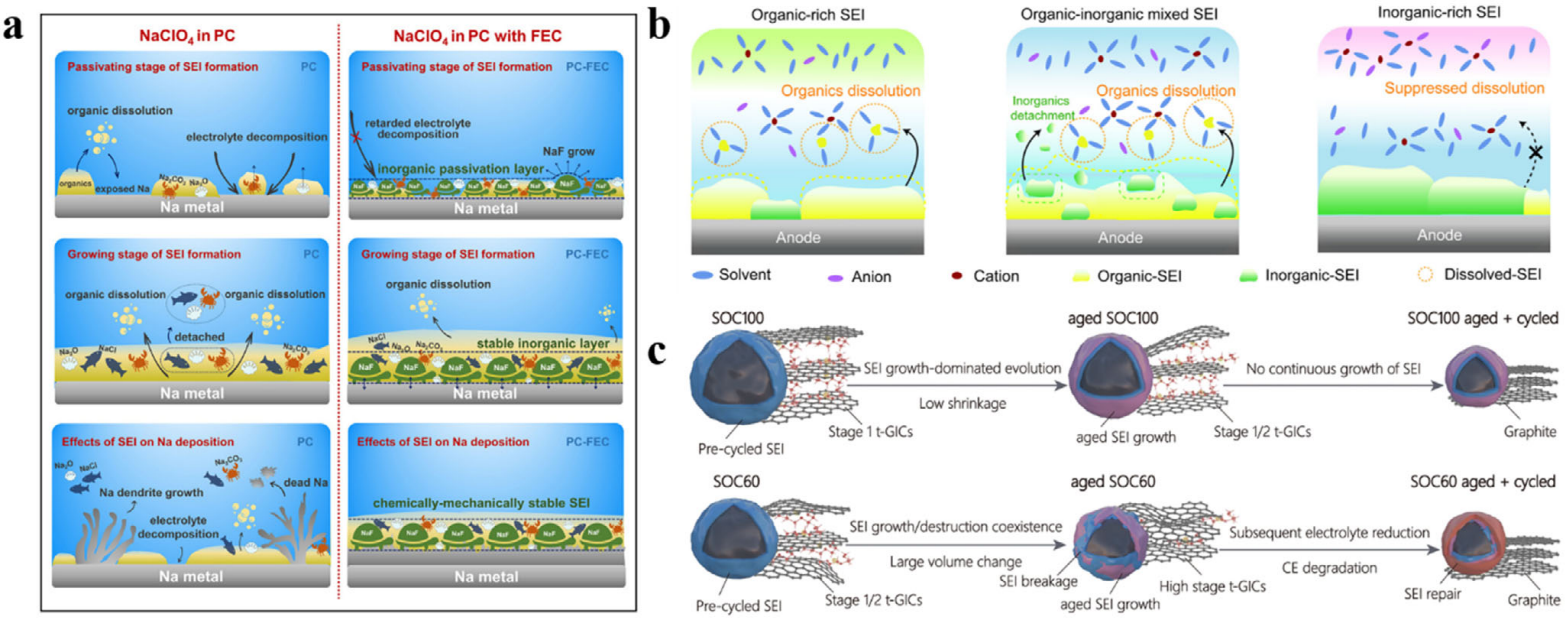
a) Schematic illustration of SEI formation.^[^ ^294^ ^]^ Copyright 2023, Elsevier. b) Schematic diagram of dissolution behavior of SEI with different components.^[^ ^295^ ^]^ Copyright 2024, Wiley‐VCH. c) Schematic diagram of SEI aging comparison of graphite anode at different SOC.^[^ ^298^ ^]^ Copyright 2024, Royal Society of Chemistry.

The solubility of sodium compounds in organic solvents is greater than that of lithium compounds (NaF > LiF, Na_2_CO_3_ > Li_2_CO_3_).^[^ ^276^ ^]^ Reza Younsei et al.^[^ ^21^ ^]^ designed an experiment to directly compare the solubility behavior of salts in the same electrolyte system in SIB and LIB: after pre‐cycling 10 times to form a stable SEI, it was paused for 100 hours, and the voltage change during the pause was monitored. When the voltage increased during the pause, it meant that the alkali ions were consumed. It was found that the voltage change in SIB was significantly greater than that in LIB, corresponding to capacity loss. Namely, SIB lost 71 mAh g^−1^, while LIB lost 38 mAh g^−1^, equivalent to 80% of the sodium battery's charging capacity, whereas the lithium battery's charging capacity was only 20%. Therefore, the SEI of SIB appears more soluble than that of LIB, which continuously decomposes the electrolyte and regenerates SEI. Yang et al.^[^ ^294^ ^]^ investigated the origin of the dissolution and instability of SEI on sodium metal anode through a series of in situ characterization techniques. They pointed out that the high solubility of some organics in SEI leads to the repeated formation and dissolution of organic components in SEI. When the FEC additive is introduced, an inorganic passivation layer (mainly NaF) is formed on the electrode surface in advance; then some organics grow on the inorganic layer to form a layered structure, and the SEI with this structure can effectively resist the growth of dendrites. In situ quantification of SEI dissolution mass loss using electrochemical quartz crystal microbalance revealed that the inorganic‐rich interface formed by FEC can effectively prevent SEI dissolution (**Figure** **23**a). Zhu et al.^[^ ^295^ ^]^ investigated the effects of SEI components and electrolyte composition on the solubility of SEI on the hard carbon surface of SIBs, and similarly revealed that some of the organics in SEI are more soluble. This is because it has a “like dissolves like” mechanism with the organic electrolyte. By controlling the salt concentration, they designed three types of SEIs, i.e., organic‐rich, hybrid, and inorganic‐rich ones; surprisingly, the solubility of the hybrid SEI was greater than that of the organic‐rich SEI. Through analysis of the SEI structure, it is believed that the dissolution of organic components leads to the consequent detachment of some of the inorganic components that have been growing on the surfaces (Figure 23b), resulting in obvious damage to the SEI. In short, the components and structure of SEI seriously affect the solubility of SEI. It seems that the solubility problem of SEI can be drastically alleviated when there are more inorganic components in SEI. It is important to understand that the dissolution of SEIs is now often quantified by in situ electrochemical quartz crystal microbalance (EQCM),^[^ ^296^ ^]^ which facilitates our understanding of the dissolution mechanism. As the SEI begins to dissolve, the electrolyte in direct contact with the electrode surface starts to decompose. This cyclic process accelerates the degradation of the battery capacity, so that it is necessary to introduce more low‐solubility components when constructing the ideal SEI for SIBs.

In the process of charging and discharging, the electrode material often undergoes volumetric expansion and structural deformation. SEI with insufficient mechanical strength is difficult to resist the huge deformation and thus rupture, resulting in weakened contact between the electrode and electrolyte. The lack of protection by a robust SEI film will accelerate the deterioration of the interface.^[^ ^297^ ^]^ Zhai et al.^[^ ^298^ ^]^ investigated the volumetric expansion and the SEI evolution process on the graphite anode of SIBs. Graphite, as a SIB anode, operates through the intercalation mechanism of ether electrolytes, and this can cause over 300% volumetric expansion and damage the SEI. By establishing a link to the state of charge (SOC), it was found that the aged graphite anode at SOC60 exhibits more capacity loss (Figure 23c). Through multi‐scale characterization of SEI, the specific reason for SEI damage was due to the enormous volumetric expansion of graphite anode during the formation of intercalation compounds. As for cathode materials, transition metals (TMs) dissolved at the cathode interact with the interfacial film, which is known as the crosstalk effect.^[^ ^299^ ^]^ Due to the presence of trace water in the electrolyte, the electrolyte undergoes a side reaction to generate acids, accelerating the dissolution of TMs and CEI/SEI. In particular, some layered oxide cathode materials, in which Ni and Mn elements show stronger Lewis basicity due to more pronounced electron delocalization of the long‐axis‐oriented M─O bonds, have a stronger electron loss capacity and are easily dissolved by reaction with acids.^[^ ^300^ ^]^ A good CEI film can protect cathode materials and inhibit the dissolution of TMs. Researchers use electrolyte engineering and artificial interfacial methods to construct an ideal CEI. Similar to SEI, the CEI is crucial for battery cycling performance, but is less researched, especially in SIBs. Care should be taken that side reactions weigh more when the battery is operated in harsh environments, such as high/low temperatures and high‐current charging. Electrolytes are more prone to decomposition at high temperatures, which accelerates the rupture of CEI. While at low temperatures, the charge transfer rate drops significantly, and the desolvation process slows down, leading to severe concentration polarization on the anode surface and a rise in interfacial impedance. At low temperatures, the rate of Na^+^ entering the anode decreases, which can easily lead to excessive deposition of Na^+^ and accelerate the formation of dendrites.^[^ ^301^, ^302^ ^]^

**Figure 24:**
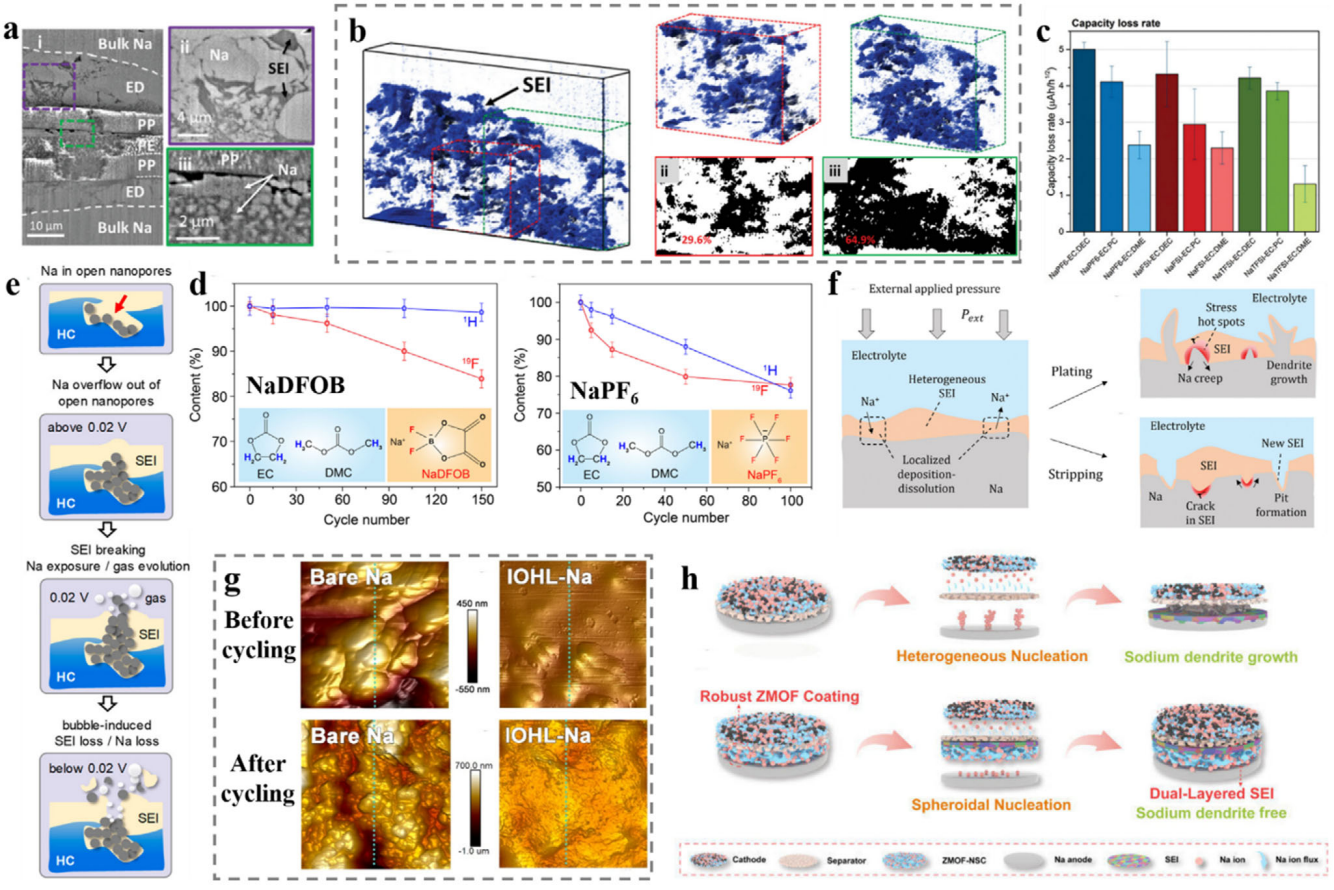
a) Cross‐sectional SEM image of Na||Na symmetric cell after cycling.^[^ ^303^ ^]^ Copyright 2024, Wiley‐VCH. b) 3D reconstruction image of SEI.^[^ ^303^ ^]^ Copyright 2024, Wiley‐VCH. c) Capacity loss rate of different electrolytes.^[^ ^304^ ^]^ Copyright 2023, Wiley‐VCH. d) In situ NMR measurements using different electrolytes.^[^ ^305^ ^]^ Copyright 2022, The American Association for the Advancement of Science. e) Schematic diagram of Na penetration through SEI below 0.02 V.^[^ ^307^ ^]^ Copyright 2024, American Chemical Society. f) Schematic diagram of structural changes during plating/stripping process of heterogeneous SEI.^[^ ^308^ ^]^ Copyright 2025, Wiley‐VCH. g) AFM images of Bare Na and IOHL‐Na before and after cycling.^[^ ^311^ ^]^ Copyright 2023, Wiley‐VCH. h) Schematic diagram of the action mechanism of MOF coating.^[^ ^312^ ^]^ Copyright 2024, American Chemical Society.

SEI is difficult to maintain completely stable due to its dynamic nature. During the cycle process, it continuously consumes electrolyte and constantly undergoes restructuring and regeneration, leading to thickening of the SEI until the battery fails. To analyze the composition and structural changes of SEI, advanced dynamic in situ characterization techniques are required. Warner et al.^[^ ^303^ ^]^ designed an experimental method to directly observe undismantled Na||Na symmetrical cells using cryogenic‐electron microscopy and analyzed the failure mechanism of the sodium anode. The presence of SEI was observed in the cell cross‐section (**Figure** **24**a). A 3D reconstruction of the entire unassembled battery (Figure 24b) showed that SEI was distributed inside the cell and may have accumulated at the interface between the Na anode and the separator. The SEI can be divided into low‐density regions (displayed by red box) and high‐density regions (displayed by green box). This uneven SEI may lead to electrolyte infiltration, and continuous decomposition, causing the SEI to break and regenerate throughout the process, and ultimately battery failure. Since SEI restructuring consumes alkali metal ions, causing battery capacity loss, Younesi et al.^[^ ^304^ ^]^ quantified the capacity loss during SEI formation, dissolution, and restructuring in SIBs. They used different electrolyte compositions to investigate SEI stability. Specifically, they used a repeated cycle‐open circuit pause cycle method, combined with GC‐MS, NMR, and XPS techniques, to investigate the relationship between SEI thickness‐composition and capacity loss. Figure 24c summarizes the capacity loss rate in different electrolytes. When EC/DME solvent is used, the capacity loss is the least, and there is no definite relative relationship with sodium salt. This also indicates that the influence of the solvent on capacity loss is greater than that of the salt. After a 50‐h pause, the capacity loss of the EC/DEC electrolyte can reach 25–35 µAh, with losses below 10 µAh possibly caused by SEI dissolution, while the remaining losses are attributed to the regrowth of SEI during subsequent. The decrease in solvent concentration during the pause time also indicates that the electrolyte is continuously consumed. Since the cycling process operates on a long‐term basis, the capacity loss caused by SEI reconstruction accounts for a significant proportion in the battery. Through this study, we also found that the composition of the electrolyte (mainly the solvent) has a significant impact on the subsequent SEI restructuring behavior. Besides, Kong et al.^[^ ^305^ ^]^ investigated the effect of NaDFOB on electrolyte consumption during cycling. Firstly, DFOB^−^ can decompose preferentially, participating in SEI formation, thereby enhancing battery cycling stability. Through in situ NMR tests (Figure 24d), the consumption of various components in the electrolyte during battery cycling was quantified. When PF_6_^−^ was used, the ^1^H and ^19^F signal intensities continued to decrease, indicating that the solvent and anion continued to decompose during cycling; however, when DFOB^−^ was used, the ^1^H signal intensity remained almost unchanged during cycling, indicating that the SEI formed by DFOB^−^ participating in decomposition effectively suppressed the decomposition of the organic solvent. This work shows that anions can fully influence the SEI reconstruction behavior, but unlike solvents, they mainly do so by pre‐forming a stable SEI, thereby avoiding electron tunnelling and inhibiting solvent decomposition.

Repeated electrochemical reactions and the related electrode expansion/contraction during the cycle process can lead to mechanical failure of the SEI. It will cause direct contact between the electrode and the electrolyte, thereby consuming the electrolyte, repeatedly forming the SEI, and leading to battery deactivation or more severe safety issues.^[^ ^306^ ^]^ For SIBs, the low voltage plateau of the HC anode is close to the sodium plating potential, which easily causes sodium dendrite formation and SEI rupture. Qiao et al.^[^ ^307^ ^]^ analyzed the sodium storage behavior and SEI evolution of HC electrodes at different cut‐off voltages using titration mass spectrometry (TMS). Comparing HC anodes with cut‐off voltages of 0.2 V and 0 V, it was observed that the contents of dead Na, NaH, and solid carbonate in the 0 V electrode were reduced. Based on the analysis of the sodium storage mechanism using online electrochemical mass spectrometry (OEMS), a unique Na^+^ reaction pathway was proposed (Figure 24e). At voltages between 0.1 and 0.02 V, Na exists in a quasi‐metallic state within the open pores. Once the open pores are completely filled, some Na begins to exist below the SEI. As the voltage continues to decrease, an increasing amount of Na penetrates the SEI, to rapture the SEI in company with the generation of gas. However, when FEC as additives are introduced, the derived SEI becomes thinner and more stable, thereby suppressing SEI rupture. This work helps understand the mechanical failure mechanism of SEI in HC. In parallel, Mukherjee et al.^[^ ^308^ ^]^ investigated the impact of SEI heterogeneity on battery stability and failure mechanisms. They found that current tends to concentrate in low‐resistance SEI regions, thus depositing Na unevenly. After cycling, new SEI forms on the unevenly deposited Na in the form of pits (Figure 24f). Simulations of SEI with different structures showed that the formation of pits increases mechanical stress at the interface during the electroplating/stripping process. Furthermore, the stress during the Na dissolution process is greater than during the deposition process, and this imbalance accelerates SEI dissolution and pitting growth. Therefore, constructing a structurally uniform SEI is crucial to avoid mechanical damage during cycling.

Electrodes such as Na metal inevitably undergo volume expansion during cycling, and this dynamic volume change exerts enormous mechanical stress on the SEI covering their surface. Although the brittle SEI exhibits good chemical stability. It is difficult to withstand the volume deformation of the electrode, leading to cracks and fragmentation. Once the SEI ruptures and the fresh electrode surface is exposed, it will trigger new and intense interface reactions, consume Na^+^ and electrolyte, and form a thicker and more uneven SEI, which paradoxically accelerates interface degradation.^[^ ^309^ ^]^ An ideal SEI should not only be robust but also a “dynamically adaptive” interface layer capable of maintaining its protective integrity while withstanding a certain degree of deformation. How to achieve this can first start with the components of SEI. Current research on the SEI focuses on introducing more inorganic components, as an inorganic‐rich SEI can promote ion transport and enhance stability. However, organic components possess excellent flexibility, which can alleviate stress caused by volume expansion and reduce the risk of SEI rupture during cycling.^[^ ^310^ ^]^ Therefore, researchers began to construct inorganic/organic hybrid SEIs. For example, Jiao et al.^[^ ^311^ ^]^ prepared a solution composed of SnCl_2_ and 4‐chloro‐2,6‐dimethylphenol, which reacted with Na metal to produce a stable inorganic/organic hybrid interface (IOHL‐Na). Atomic force microscopy (AFM) characterized the different electrodes before and after cycling. IOHL‐Na clearly had a flatter surface (Figure 24g) and a higher Young's modulus than Bare‐Na (7.78 GPa vs 3.4 GPa). The synergistic effect of inorganic and organic components can complement each other, enabling sodium metal batteries to achieve long cycle life at high current densities. Some researchers have constructed multi‐layered SEI structures to address this issue. Ideally, the SEI layer closer to the electrode should have good adhesion and sufficient flexibility to adapt deformation, while the outer SEI layer should be denser and more robust to provide good chemical stability. For example, Cao et al. constructed a metal–organic framework nanostructure coating. The MOF layer is porous and ordered, providing good conductivity and mechanical strength, promoting uniform nucleation of Na, and suppressing volume expansion. Furthermore, the coating effectively anchors FSI‐, enabling the outer layer to form a robust inorganic SEI layer through anion decomposition. This dual‐layer SEI structure, with an inner MOF layer and an outer inorganic layer, effectively enhances battery stability (Figure 24h). In addition to optimizing SEI itself, electrode structures with some buffer space can also be designed, functional binders can be used for non‐metallic electrodes to enhance the overall mechanical strength of the electrode, and reduce relative displacement and local stress between particles. Resolving the contradiction between SEI mechanical robustness and electrode volume change is the key to achieving long cycle life of electrode materials with high‐volume change. This requires advanced characterization techniques to be combined to gain a deeper understanding of the failure mechanisms of the SEI under dynamic stress, thereby helping to design an SEI with optimal mechanical properties.

The aging of SEI is one of the most important factors responsible to battery capacity degradation. As for SIBs, the solubility of SEI seems to be of utmost importance. It remains under debate whether SEIs with higher inorganic content are truly effective in resisting dissolution. With the development of advanced characterization techniques, the composition and structure of SEI can be clearly analyzed. The mechanism of SEI formation and aging has been less investigated, and the concepts and models previously proposed by researchers are still being used today. If we can have a profound understanding of this process, it will be beneficial to construct an ideal SEI to improve the long‐term cycle stability of SIBs.

### 4.2 SEI Optimization Strategy

An ideal SEI should have the following characteristics: 1) dense and homogeneous structure with high mechanical strength and low solubility; 2) fast ion transport rate; 3) good electronic insulation to prevent continuous decomposition of the electrolyte; 4) being stable even under high voltage and high temperature conditions. In order to obtain interfaces with excellent comprehensive performance, researchers have considered various strategies, commonly including electrolyte design and artificial interfaces. With the growth of the battery field, more attention has been paid to the fact that many secondary factors can also have a profound impact on the performance of SEI.

#### 4.2.1 Electrolyte

SEI comes from the decomposition of the electrolyte, and naturally different SEI can be obtained by changing the electrolyte composition. This includes sodium salts, solvents, additives, and more. Some of them have been summarized in the previous sections. In fact, no matter which components are involved, they usually impose an effect on the solvation structure. Therefore, in this section, we mainly focus on the solvation structure in SIB electrolyte to summarize and elaborate on its impact and optimization on SEI.

**Figure 25:**
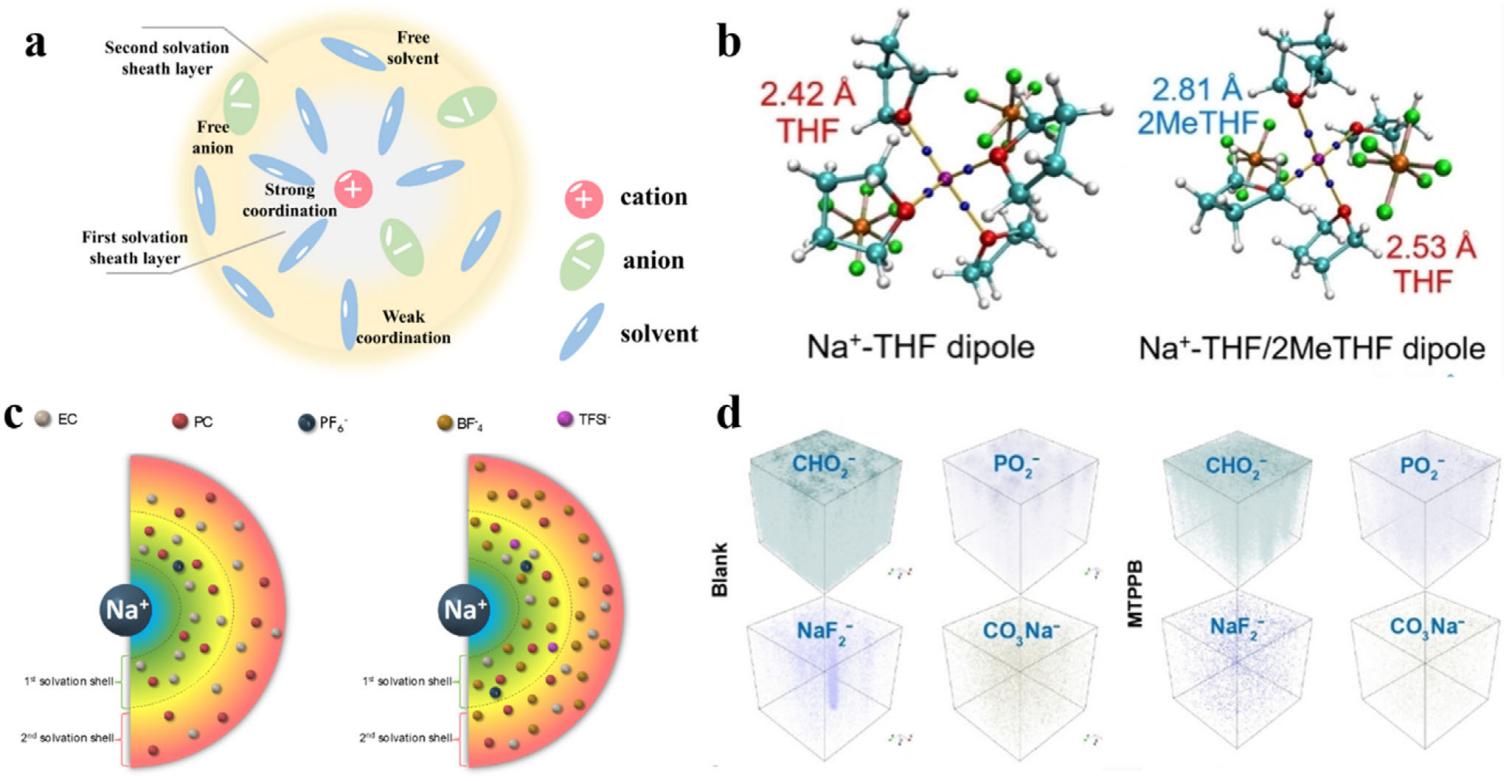
a) Schematic diagram of solvation sheath. b) Typical solvation structures using different electrolytes.^[^ ^315^ ^]^ Copyright 2024, Wiley‐VCH. c) The influence of introducing BF_4_^−^ and TFSI^−^ on the first and second solvation sheaths.^[^ ^316^ ^]^ Copyright 2024, Wiley‐VCH. d) 3D structure of SEI for Blank and MTPPB electrolytes.^[^ ^317^ ^]^ Copyright 2024, Wiley‐VCH.

Solvation sheath is a concept that describes the coordinated structure of various components in an electrolyte. As shown in **Figure** **25**a, some solvents and anions surround the cations to form a sheath layer, which is mainly divided into a first solvation layer and a second solvation layer. In the first solvation layer, the electrolyte component directly coordinates with the cations, while the second solvation shell primarily includes some weakly coordinated free components. Solvation involves various intermolecular interactions, including hydrogen bonding, ionic dipole moments, intermolecular forces, and van der Waals forces. The essence of changing the solvation structure is to alter the strength of the interactions between specific components.^[^ ^313^, ^314^ ^]^ SEI is affected by the solvation structure, and the common methods for adjusting the solvation structure of Na^+^ currently include weakening the solvent coordination and introducing anions. The two are usually complementary, that is, when the solvent coordination is weaker, anions naturally start to enter the solvated sheath, forming an anion‐derived SEI that enhances stability and ion transport kinetics.

Changing the salt concentration can straightforwardly reconfigure the solvated structure. At a high salt concentration, more anions enter the first solvation layer. The electrolyte components can also be customized to achieve the effect of weak solvation. Li et al.^[^ ^315^ ^]^ introduced 2‐methyltetrahydrofuran (MeTHF), as a weaker coordination with Na^+^, into the THF electrolyte, to weaken the overall solvation coordination (Figure 25b), accelerate the desolvation process, and induce the preferential decomposition of anions on the HC anode. As such, an inorganic‐rich SEI forms, which is conducive to the diffusion of Na^+^. Moreover, the introduction of some additives can regulate the solvation structure. Mitra et al.^[^ ^316^ ^]^ introduced two anions of high donor number (BF_4_^−^ and TFSI^−^) into conventional carbonate‐based electrolytes to force Na^+^ to coordinate with more anions and promote the participation of added anions in the reduction process to form SEI. As shown in Figure 25c, the introduction of the anions reconfigured the first and second solvated layers. The structure and composition of the SEI on the HC surface were characterized by HR‐TEM and XPS, and the SEI formed after the introduction of anions was thinner and more homogeneous, with more inorganic components. Li et al.^[^ ^317^ ^]^ introduced methyltriphenylphosphonium bromide (MTPPB) into the electrolyte to reconfigure the solvated sheath. Due to the structural properties of MTPPB, Br^−^ will be coordinated with Na^+^ through anionic interactions, which occupies the chelating site of the solvent molecule and weakens the solvent coordination. Meanwhile, MTPP^+^ and PF_6_^−^ are also coordinated due to ionic interactions. MTPPB is similar to an amphoteric additive, which achieves cross‐coordination in the electrolyte. Due to the π‐π interaction between MTPP^+^ and HC surface, it can adsorb on the HC surface and guide the targeted reduction of PF_6_^−^ anion to form a stable SEI. The 3D structure of the formed SEI was characterized by time‐of‐flight‐secondary‐ion‐mass spectrometry (TOF‐SIMS), as shown in Figure 25d. The addition of MTPPB promoted the formation of ultra‐thin and homogeneous SEI, and contained more NaF due to the decomposition of more anions.

Introducing anions into the solvation structure has become a mainstream method for regulating SEI. Due to the competition of anions, it is believed that it will weaken the coordination of solvents and accelerate the desolvation process. However, this has also been questioned. Since anions are introduced and there is coordination between cations and anions, desolvation not only includes solvents, but also the overall desolvation energy barrier should be calculated. In addition, how do anions form SEIs that are easily desolvated? To address these issues, a model for SEI‐assisted ion desolvation was proposed by He et al.^[^ ^318^ ^]^ They suggested that anion‐derived SEI actually promotes the desolvation process, mainly because the inorganic components produced by anion decomposition can adsorb cations. This work provides a new aspect of the relationship between solvated structures and SEI, and reminds us that SEI can also affect solvated structures, and it is a complex conformational relationship between both.

**Figure 26:**
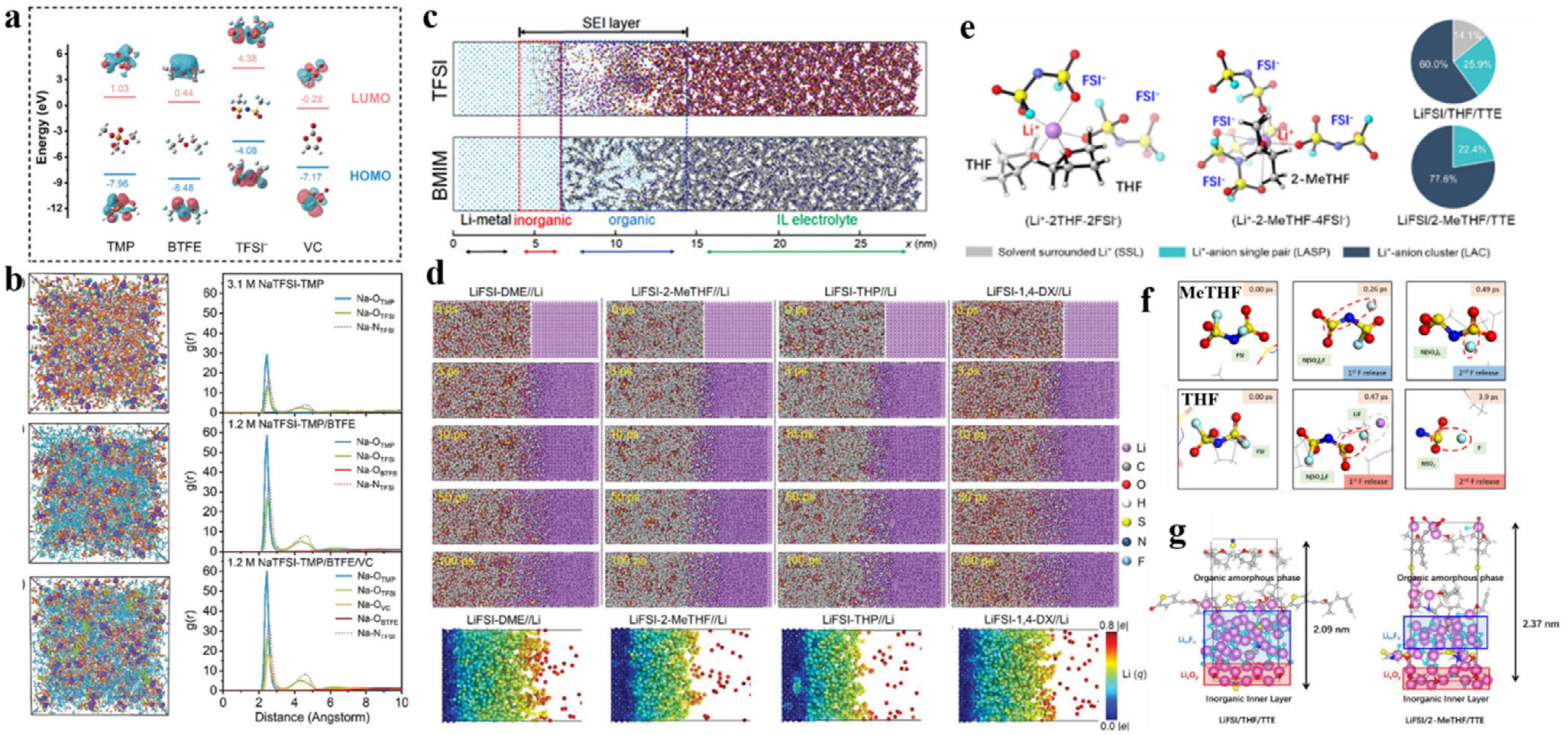
a) HOMO/LUMO energy levels of different molecules.^[^ ^319^ ^]^ Copyright 2021, Wiley‐VCH. b) MD data of different electrolytes.^[^ ^319^ ^]^ Copyright 2021, Wiley‐VCH. c) The structure of simulated SEI.^[^ ^323^ ^]^ Copyright 2022, Wiley‐VCH. d) SEI dynamically formed by different electrolytes.^[^ ^324^ ^]^ Copyright 2023, Wiley‐VCH. e) Typical solvation structures of THF and MeTHF electrolytes.^[^ ^325^ ^]^ Copyright 2024, American Chemical Society. f) Decomposition process of FSI^−^ in different electrolytes at different times.^[^ ^325^ ^]^ Copyright 2024, American Chemical Society. g) SEI formed by different electrolytes in the simulation.^[^ ^325^ ^]^ Copyright 2024, American Chemical Society.

To analyze solvation structures, theoretical calculations and simulations are now commonly used to help us understand electrochemical processes. The most widely used methods are first‐principles calculations and molecular dynamics (MD) simulations. Typical MD simulation methods include the following steps: 1) modeling; 2) setting initial conditions; 3) running the dynamics; and 4) exporting and statistically analyzing the data. Researchers set the number of molecules in each component using actual electrolyte ratios and proportional scaling, placing all electrolyte molecules in a set cube box. After running with the set parameters for a defined period of time, multiple key indicators of the electrolyte were obtained by extracting data. First‐principles calculations primarily address the issue of multi‐body interactions, with the computational principles based on density functional theory (DFT). The DFT method can effectively calculate the reduction energy levels (HOMO/LUMO) of molecules, enabling to determine the sequence of reactions among different components of the electrolyte. Currently, researchers often combine two methods to analyze the solvation structure and film formation possibility of electrolytes. For example, Chou et al.^[^ ^319^ ^]^ calculated the molecular orbitals of different components in the electrolyte (**Figure** **26**a). TFSI^−^ has the highest HOMO energy level, indicating a high priority for decomposition on the cathode surface, and therefore tends to form anion‐derived CEI. Furthermore, after MD calculation, the distribution of electrolyte molecules in the entire cube box was obtained through visualization software (Figure 26b). The radial distribution function of Na^+^ and each component was derived to evaluate and predict the solvation structure. Since the decomposition of the electrolyte to form SEI is a dynamic process that changes over time, it is necessary to us time‐resolved MD for investigation. Reactive force field (ReaxFF) is a force field developed by Van Duin, Goddard, and others that can describe chemical reactions in complex systems.^[^ ^320^ ^]^ The combination of ReaxFF and MD can significantly reduce costs in computational study, improve simulation speed, and describe the dynamic process of reactions at the atomic level. ReaxFF MD is now widely used to simulate the process of electrolyte decomposition to form SEI.^[^ ^321^, ^322^ ^]^ Given the lag in SIB development, some of the most advanced simulation technologies have not been applied to SIB in a timely manner. Therefore, we will draw on the experience gained from LIB and summarize the patterns. Goddard et al.^[^ ^323^ ^]^ established a model of ILs electrolyte and metal anode, and used ReaxFF MD to simulate the details of SEI formation at the interface. As the simulation time increased, SEI gradually grew. As shown in Figure 26c, it was found that the SEI consists of an inorganic inner layer approximately 2.5 nm thick and an organic outer layer approximately 7.5 nm thick, which is consistent with the common SEI bilayer model and confirms the accuracy of the model. This method enables effective prediction of the optimization of the interface for different electrolytes, thereby avoiding a trial‐and‐error process. Huang et al.^[^ ^324^ ^]^ used this method to simulate the SEI structure formed by four single solvents (DME, 2‐MeTHF, THP, and 1,4‐DX) in the prepared electrolyte. As shown in Figure 26d, the thickness of the SEI can be simulated based on the charge distribution, and the porosity of the SEI formed by the four electrolytes can be compared based on the structural evolution of the SEI within 100 ps. The comparison shows that the SEI structure formed by the electrolyte composed of THP solvent is the densest and thinnest. This is due to the high FSI^−^ coordination number in the THP electrolyte, which forms a rich anionic solvation structure, resulting in the most ideal SEI composition. However, the question remains if it is better to have more anions in the solvation structure. Cao et al.^[^ ^325^ ^]^ used two solvents with different solvation capabilities (strong solvation for THF and weak solvation for MeTHF, the strength comparison only lies between the two solvents) mixed with diluents and salts. In the MeTHF electrolyte, FSI^−^ coordinated more (Figure 26e). ReaxFF MD calculations were performed to investigate the decomposition steps and reaction times of FSI^−^. It was found that FSI^−^ begins to decompose at 0.26 ps in MeTHF electrolyte and undergoes a second defluorination at 0.49 ps, while in THF electrolyte, the first defluorination occurs at 0.47 ps and the second at 3.9 ps (Figure 26f). The solvation structure of more anions leads to rapid defluorination at the beginning of the reaction, resulting in a large accumulation on the electrode surface in a short period of time and forming a thicker SEI. The initial accumulation of SEI on the electrode surface affects the decomposition of other components (such as diluents) in the electrolyte. Resulting in an unbalanced SEI structure. Through simulation of the SEI structure, it was found that MeTHF electrolyte formed a thicker SEI (Figure 26g), and some other inorganic components had a low content (Li_x_O_y_).

Based on this report, we recognize that an excessive anion content in the solvation structure is not conducive to the formation of an SEI with better performance, so it is necessary to explore the optimal anion participation. To note, the formation of SEI is a time‐consuming process, and its phased, gradual formation may be more conducive to obtaining a structurally stable and functionally sound SEI.

#### 4.2.2 Coating

**Figure 27:**
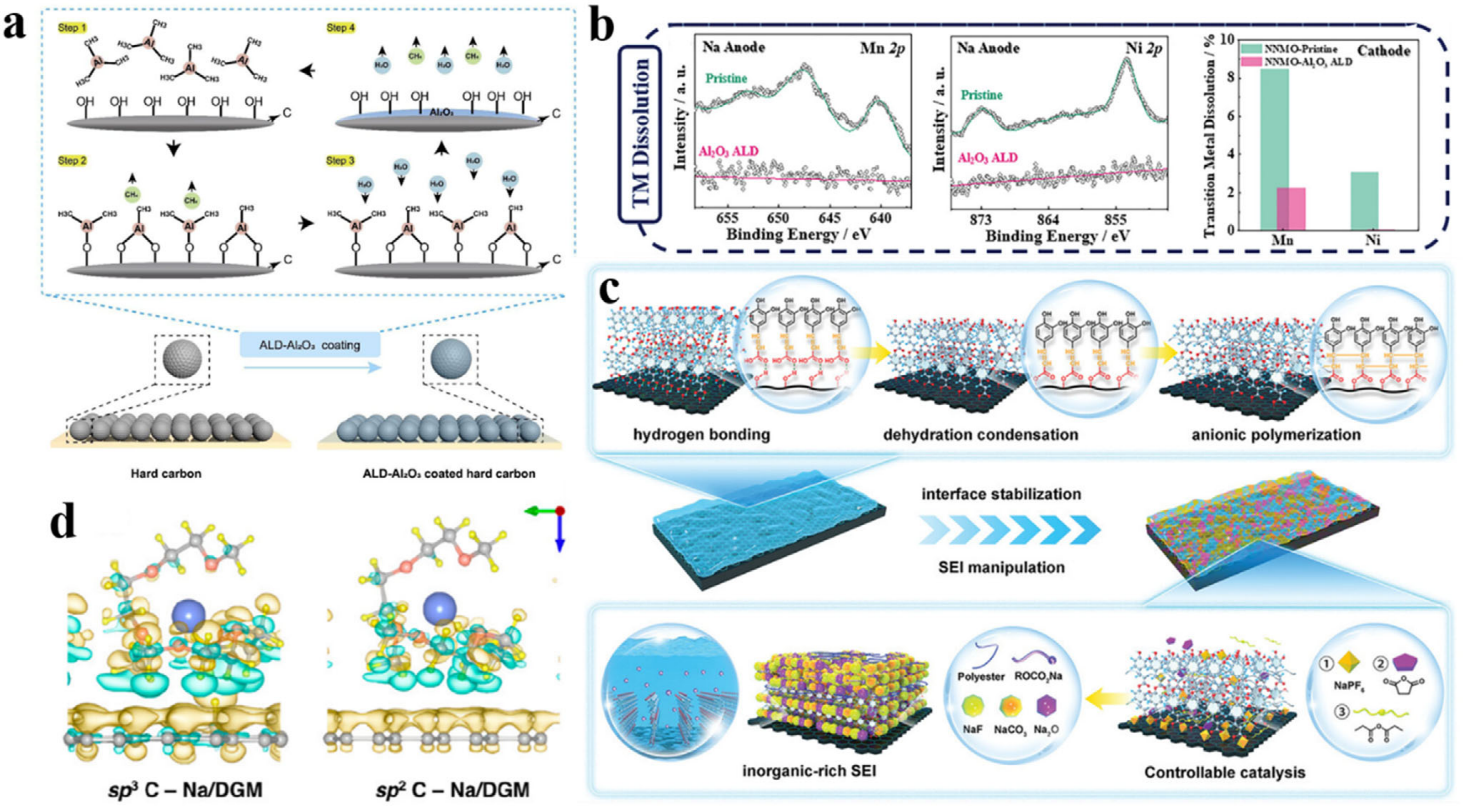
a) Schematic of the process for depositing Al_2_O_3_ on the surface of the HC.^[^ ^328^ ^]^ Copyright 2019, Elsevier. b) Characterization of transition metal dissolution on Na anode surface.^[^ ^333^ ^]^ Copyright 2021, Wiley‐VCH. c) Schematic diagram of strategy for grafting poly CA onto HC surface to improve SEI.^[^ ^334^ ^]^ Copyright 2023, Wiley‐VCH. d) Comparison of the interaction between *sp^2^* C and *sp^3^* C with solvents.^[^ ^335^ ^]^ Copyright 2024, American Chemical Society.

Besides electrolyte decomposition, constructing a protective coating with SEI function on the electrode surface can inhibit Na^+^ depletion and ensure stability. Ideal coatings should have high ionic conductivity and high mechanical strength, and the coating itself should be chemically inert and able to block electron transfer.^[^ ^326^, ^327^ ^]^ Based on the requirements, Al_2_O_3_ has been proposed as a passivation layer, and has been widely used in the cathode/anode of SIBs. For example, Cao et al.^[^ ^328^ ^]^ coated a layer of Al_2_O_3_ material on the surface of HC by atomic layer deposition (ALD) (**Figure** **27**a). As an artificial SEI, it inhibits electrolyte decomposition, reduces Na^+^ consumption, and improves ICE and cycle stability. In addition to metal oxides such as Al_2_O_3_, some metal phosphates and metal fluorides are often applied as inert coatings to stabilize electrode materials.^[^ ^329^, ^330^ ^]^ These are all inert metal coatings, in addition to common carbon coatings and core‐shell structure coatings.^[^ ^331^ ^]^ For example, researchers have introduced zirconium doping and carbon coating into the Na_4_MnV(PO_4_)_3_ (NMVP) cathode, and the carbon coating effectively enhances the conductivity of the material.^[^ ^332^ ^]^

For cathode materials, artificial coatings can hinder the phase transition and dissolution of transition metals. Xiao et al.^[^ ^333^ ^]^ protected Na_2/3_Ni_1/3_Mn_2/3_O_2_ cathodes by using four metal oxide coatings (Al_2_O_3_, TiO_2_, SnO_2_, and WO_3_) and compared their enhancement effect in performance. The Al_2_O_3_ coating was found with the best results, and the dissolution of transition metals on the surface of the Na anode was characterized by XPS and ICP‐AES (Figure 27b). It was found to restrict the dissolution of the transition metals, so to enhance the stability of the SIBs.

Besides some inert coatings, there are also some functionalized coatings that attempt to build up a connection with the electrolyte. Bai et al.^[^ ^334^ ^]^ grafted polymerized caffeic acid (poly‐CA) in situ onto the HC surface, where many C═O bonds were exposed on the surface (Figure 27c). These C═O acted as active centers to adsorb PF_6_^−^, which controllably catalyzed the preferential decomposition of the anion to induce an inorganic‐rich SEI. A similar strategy was proposed by Huang et al.^[^ ^335^ ^]^ to introduce the concept of weak solvation to the coating. Specifically, *sp* ^2^ C‐rich carbon coatings formed on the HC surface via pitch. It was found by DFT calculations (Figure 22d) that *sp^2^* carbon has stronger interactions with PF_6_^−^ and repels the solvent, so that the *sp^2^* C/electrolyte system can shield the solvent molecules and allow PF_6_^−^ to construct SEI at the interface to ensure the high ICE of HC.

#### 4.2.3 Other Strategies

Other factors that have been previously applied to lithium rechargeable batteries, such as mechanical factors, electrode potentials, magnetic fields, and local electric fields,^[^ ^336^, ^337^, ^338^ ^]^ are also studied in SIBs. Li et al.^[^ ^339^ ^]^ changed the EDL composition by applying or removing a localized electric field to directly control the formation of SEI. When no electric field was present, there were more anions in the EDL, and the formed SEI was more inorganic‐rich and thinner. Although the working principle of SIBs is similar to that of LIBs, there are differences in the characteristics of SEIs, so it is necessary to specify the influencing factors and modification strategies of SEI in SIBs in a targeted way.

### 4.3 No SEI?

The decomposition of electrolytes to form SEI during the first cycle has become our consensus. Because of the proposal of the SIB ether electrolyte co‐intercalation mechanism, some studies have questioned the physical existence of SEI in this specific situation. If SEI does exist, could it be a hindrance to the co‐insertion layer? Based on these issues, a number of studies have been carried out.

**Figure 28:**
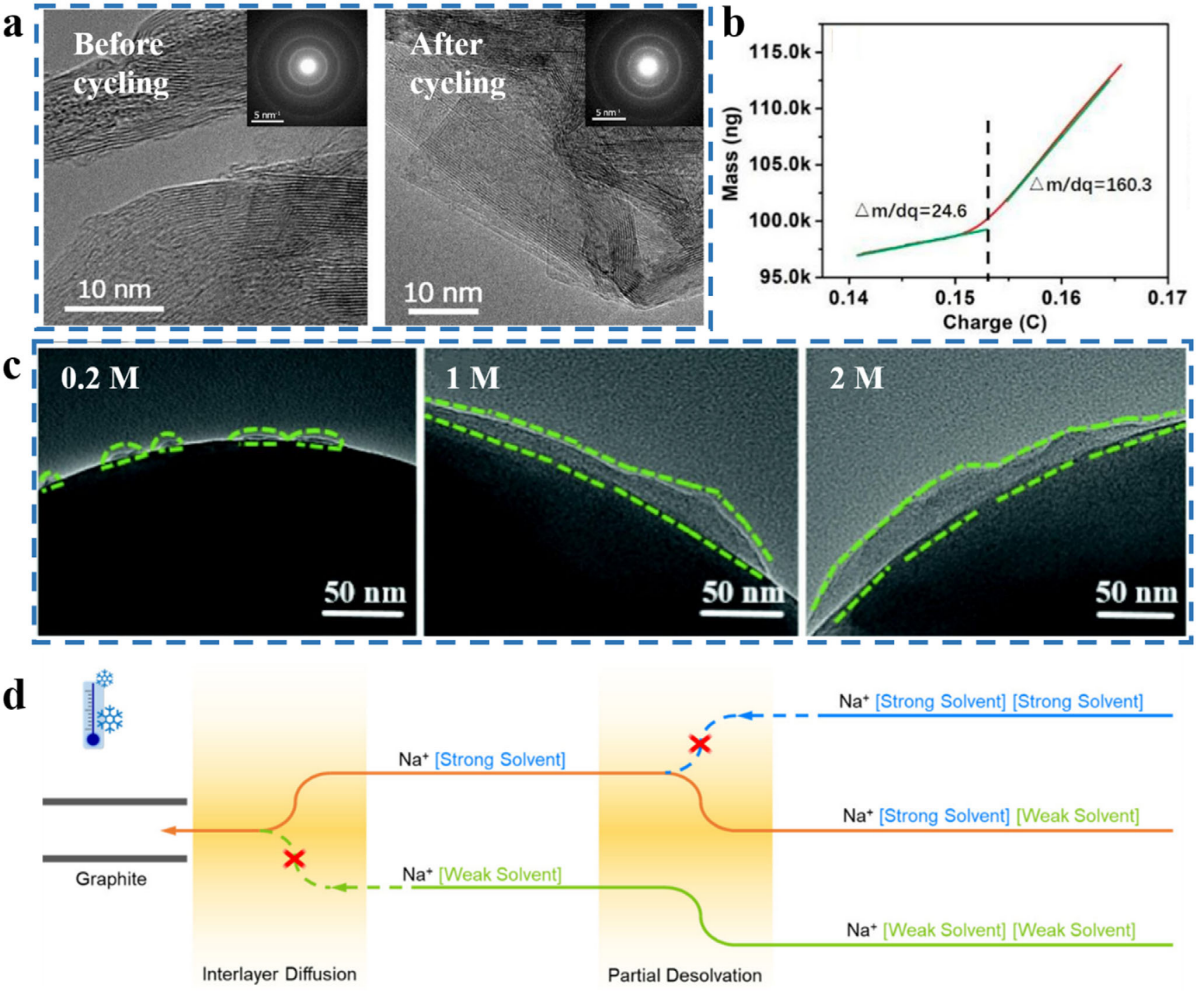
a) TEM images of graphite anode before and after cycling.^[^ ^340^ ^]^ Copyright 2018, Wiley‐VCH. b) Mass changes of hard carbon anode during discharge process.^[^ ^341^ ^]^ Copyright 2021, American Chemical Society. c) TEM images of SEI formed at different sodium salt concentrations.^[^ ^344^ ^]^ Copyright 2023, Elsevier. d) Schematic mechanism of partial desolvation and interlayer diffusion for different electrolytes.^[^ ^345^ ^]^ Copyright 2025, American Chemical Society.

Adelhelm et al.^[^ ^340^ ^]^ believed that graphite, as the SIB co‐intercalated anode, did not form SEI on the surface during the first cycle. They characterized the cycled graphite by TEM (**Figure** **28**a) and were unable to observe the presence of SEI. Moreover, the decomposition and gas production of the electrolyte during cycling were investigated by online electrochemical mass spectrometry (OEMS). They proposed that the electrolyte is not non‐decomposable, but the products from decomposition are soluble, and it is difficult to form a solid interface. Yang et al.^[^ ^341^ ^]^ shifted their research to HC anodes, which have a larger layer spacing and less volume change than graphite. They also discovered that ether electrolytes do not form SEI due to the presence of co‐intercalation mechanism. The change in electrode mass during cycling was analyzed by in situ electrochemical quartz crystal microbalance (Figure 28b), supporting no SEI generation. Since these results are based on the hard carbon anode, they believe that solvent decomposition and subsequent SEI formation may still occur on other electrode materials, particularly those containing transition metals.

The above‐mentioned studies propose a new perspective on battery operation without SEI, which is still under debate in the field. Wu et al.^[^ ^124^ ^]^ observed the presence of SEI on the surface of the HC anode using an ether electrolyte. Additionally, some groups are prone to believing that the ether electrolyte forms an SEI that is too thin to impede the co‐intercalation.^[^ ^342^ ^]^ There is also a view that such SEI exists in a different form from traditional SEI.^[^ ^343^ ^]^ Xing et al.^[^ ^344^ ^]^ proposed the formation of a discontinuous SEI on the HC surface at a salt concentration of 0.2 M (Figure 28c), whereas the formed SEI would uniformly cover the electrode surface to hinder the solvent co‐embedding at the sodium salt concentrations of 1 M and 2 M.

Recently, Liu et al.^[^ ^345^ ^]^ investigated the Na^+^ co‐intercalation mechanism in graphite and proposed, for the first time, a neglected partial desolvation behavior. Specifically, the most stable solvation structure of Na‐G2 is the coordination of two solvent molecules with Na^+^ (Na‐[G2]_2_). However, calculations revealed that the intercalation energy of Na‐[G2]_1_ (−0.3067 eV) is lower than that of Na‐[G2]_2_ (‐0.2242 eV), it indicates that when co‐intercalation occurs, the solvation structure tends to enter the carbon layer in the form of Na‐[G2]_1_. This leads to the partial desolvation of the solvent (one G2 molecule), which was overlooked in previous work. When compared with weakly solvated electrolytes and mixed electrolytes, it was found that due to the slow desolvation of the strongly coordinating solvent (G2), the kinetic rate is limited during the partial desolvation step, resulting in poor low‐temperature performance. The changes in the solvation structure of the three electrolytes during diffusion are shown in Figure 28d. It is worth mentioning that this work also identified the presence of SEI on the graphite surface.

In summary, we attempt to propose a unified interface model to the field. When the electrolyte diffuses to the vicinity of the electrode double layer, the solvation structure changes. It forms a single solvent‐coordinated solvation structure with a low intercalation energy barrier, which promotes the embedding of solvent and Na^+^ into the carbon layer together. Therefore, a partial desolvation process must occur prior to intercalation, and the formation of SEI is likely due to the decomposition of these desolvated solvents. Some researchers have not found SEI, possibly because the SEI structure formed under co‐intercalation behavior is different from the conventional one. After all, SEI is derived from solvents and may dissolve or not be dense, resulting in undetectable SEI. The non‐dense SEI also explains why it does not prevent solvents from entering the interlayer. Nevertheless, the formation of SEI in the presence of co‐intercalation remains a subject of debate awaiting for further exploration.

## 5 Summary and Perspectives

**Figure 29:**
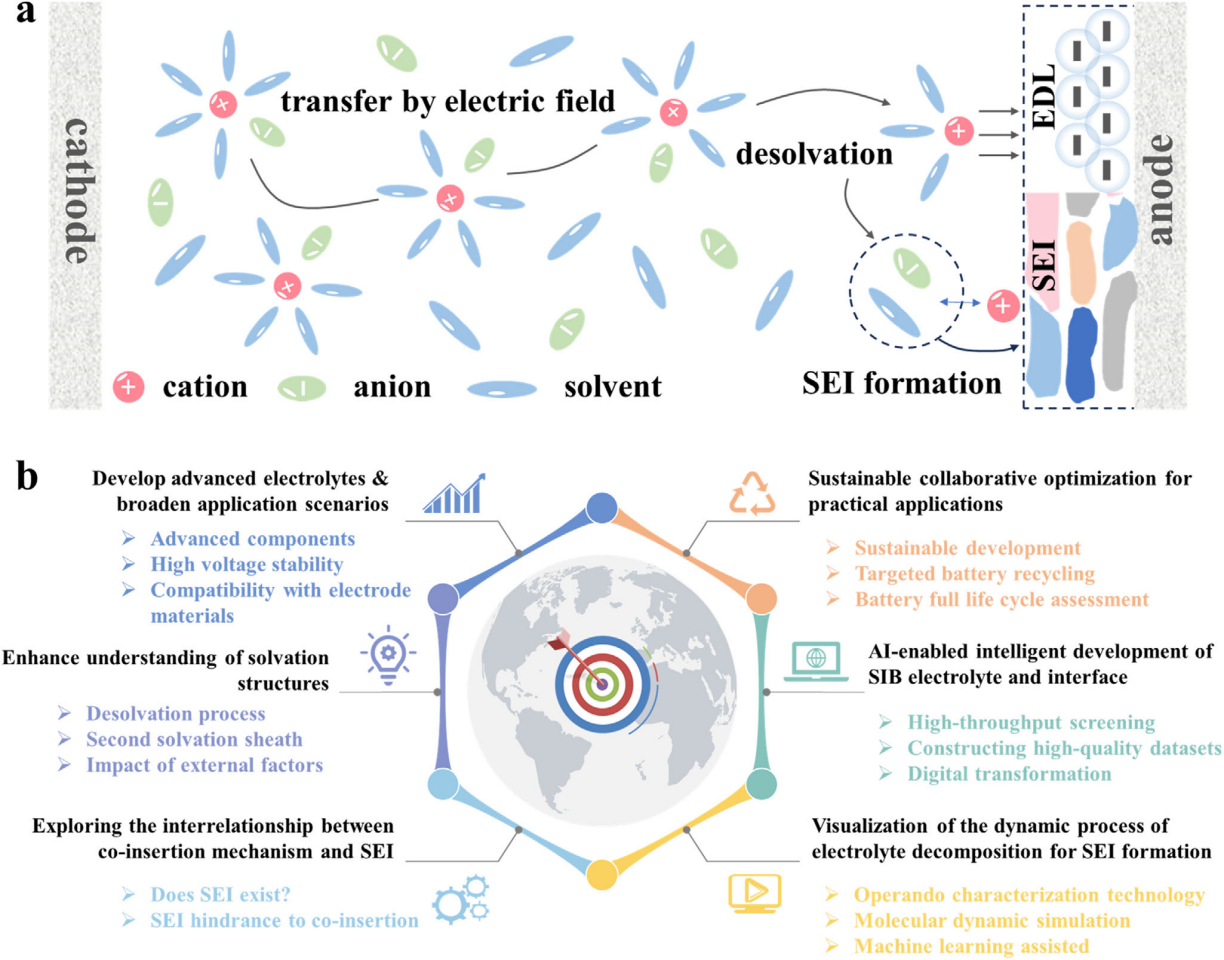
a) Schematic diagram of the ion migration process at the electrolyte and interface. b) Perspectives on future research directions for SIB electrolytes and interfaces.

In this review, we provide an overview of the research progress on SIB electrolytes and the related interface chemistry. As shown in **Figure** **29**a, Na^+^ is transported near the anode EDL through the electrolyte. After desolvation, some components undergo a reduction reaction to form the SEI film. The interface and electrolyte are the main channels and media for the ion transport process, and affect the battery performance. Therefore, starting from the components of electrolytes, we summarize existing electrolyte systems and evaluate their pros and cons in application scenarios. We notice the crucial impact of the solvation structure on electrolyte properties and the interface formation. How to optimize solvation sheaths has become a research hotspot. As for organic ether electrolytes, the unique co‐intercalation mechanism accelerates the ion transport rate, while it also raises the question of whether SEI is truly formed. Despite arguments, the formation of SEI is well accepted to stem from the decomposition of the electrolyte. In order to ensure the long‐term stability of the battery, SEI should have properties such as low solubility and high mechanical strength. Some recent studies prove that SEI interact with the electrolyte, including accelerated desolvation, and induced decomposition. Therefore, in our view, SEI and electrolytes are “twins” and inseparable. In order to promote the practical application of SIBs, the integration of AI techniques will be conducive to accelerating the development of materials and exploring the underlying mechanism. Meanwhile, taking advantage of SIBs’ low cost and abundant resources, sustainable battery recycling technology will help achieve the unity of high performance, low cost, and ecological friendliness. Considering the above elaboration on the SIB electrolyte and interface issues, we provide a summary and perspective as follows (Figure 29b) in the hope of inspiring more to explore these issues in depth, thus facilitating the commercialization of SIBs.

### 5.1 Fundamental Mechanism Research and Deepening Understanding

#### 5.1.1 Enhance Understanding of Solvation Structures

The solvation structure can affect ion transport, SEI formation, and the stability of the electrolyte. The current research has expanded beyond the simple interaction between solvents and cations to the regulation of solvation structures by nearby components and their impact on electrochemical performance. Although weakening the coordination between solvents and cations is considered a universal strategy to accelerate desolvation and improve rate performance, this viewpoint remains controversial: weakening solvent coordination may enhance anion coordination, and the interaction between anions and cations is usually stronger than that between solvents and cations. Therefore, whether this truly accelerates the desolvation process is to be verified. A more accurate evaluation should be based on the complete desolvation energy barrier of all coordination components. In addition, when evaluating the possibility of electrolyte decomposition to form SEI, it is necessary to consider the overall solvation structure rather than the HOMO or LUMO energy levels of a single component. The calculation of the density of states in a specific electrolyte environment may be more accurate.^[^ ^346^ ^]^ The solvation sheath includes the first solvation layer and the second. Although the interaction between the second solvation layer is relatively weak, it is evident that the desolvation process starts first from the second solvation layer. Some recent work has focused on the impact of the second solvation layer,^[^ ^347^, ^348^ ^]^ but there is a lack of relevant studies in SIBs. Thanks to the progress of advanced characterization and theoretical simulation tools, we are able to get a more accurate picture of the solvation structure. Nevertheless, a deep understanding of its dynamic changes during ion transport is largely missing. Studies are also needed to explore how film‐forming components build up stable SEI through desolvation, and the influence of external factors such as temperature and electric field.

#### 5.1.2 Explore the Interrelationship between the Co‐Insertion Mechanism and SEI

The co‐intercalation mechanism avoids the influence of volumetric expansion on the cycle performance, accelerates the ion transport, and improves the rate performance. However, there is a controversy over whether SEI truly exists when there is a co‐intercalation. If SEI actually exists, it may hinder the co‐embedding of solvents. Some believe that ether electrolytes can form a thinner SEI, which does not hinder co‐embedding. However, if an SEI formed is uniform and dense, even a thin layer will repel the solvent. If no SEI is formed, a question to be answered is what happens to the components of the electrolyte that are supposed to decompose under voltage drive. One possible explanation is that the SEI structure formed in the presence of co‐intercalation behavior is different from the conventional one, and may undergo dissolution or may be inherently less dense, thus enabling the solvent to enter the interlayer from the voids. Further investigation and characterization of the SEI structure formed under co‐intercalation behavior are needed to address this myth.

### 5.2 Advanced Technology Application and Innovation

#### 5.2.1 Visualize the Dynamic Process of Electrolyte Decomposition for SEI Formation

The desolvation and partial decomposition of the electrolyte to form SEI is a complex and dynamic process. Due to the closed system of the cell, it is difficult to characterize and simulate this dynamic process. Possible decomposition behaviors and SEI structures are inferred from the electrochemical properties and material characterization after cycling. With the latest development of spectroscopic characterization and simulation techniques, more and more researchers are striving to visualize the dynamic process of “electrolyte decomposition forming SEI”, which helps verify the connection between the electrolyte and the interface, and analyze the underlying internal mechanisms. Theoretical calculations reduce the trial‐and‐error costs of material development and help explore underlying mechanisms. Typical computational methods include first‐principles calculations based on DFT, which are primarily used to evaluate system interactions and energetics. Meanwhile, MD simulations are playing an increasingly critical role in investigating electrolyte solvation structures, ionic conductivity, and interfacial reaction mechanisms.^[^ ^349^ ^]^ With the introduction of advanced simulation methods, the dynamic evolution process of electrolyte decomposition and SEI formation has become traceable. The rechargeable battery during the charging and discharging process is considered as a “black box”, in which dynamic processes such as ion transfer, desolvation, and SEI formation and aging, are eager to understand but difficult to capture, which undoubtedly retards mechanism investigation and material development. Therefore, the combination of theoretical simulation and advanced characterization to visualize the internal changes of the battery can promote the development of SIBs.

#### 5.2.2 AI‐Enabled High‐Throughput Screening of SIB Electrolyte and Interface

The discovery of new electrolytes for SIBs in the future will rely deeply on breakthroughs in AI technology. Traditional electrolyte design relies on “trial and error” and limited empirical models, making it difficult to deal with scientific challenges such as the dynamic evolution of solvation structure, the complexity of interfacial reactions, and the synergistic effect of multiple components. By integrating high‐throughput computation, molecular dynamics simulation, and accessible experimental data, AI can construct precise mapping relationships between electrolyte components, solvation structure, and interfacial stability.^[^ ^350^, ^351^ ^]^ It can also explore the combination of new additives and solvents through the generation of a confrontation network (GAN), further strengthen the learning algorithm, and optimize the electrolyte formula to meet the working conditions under different temperatures and voltages.^[^ ^352^ ^]^ AI has powerful learning capabilities. For example, Xiang et al.^[^ ^353^ ^]^ developed a tool called ByteDance Artificial intelligence Molecular simulation Booster (BAMBOO), which learns complex molecular behavior from quantum mechanics simulations and uses it for MD simulations to predict the density, viscosity, ionic conductivity, and other properties of electrolytes. To date, there are few activities in this part. AI‐driven electrolyte development still needs to break through the key problems, such as the construction of high‐quality datasets, the improvement of the accuracy of multi‐scale simulation, and experimental verification. In any case, with the continuous development of AI technology, electrolyte and interface design are expected to move from “empirical trial and error” to a new paradigm of “digitalization”, accelerating the innovation process of high‐performance SIBs.

### 5.3 Practical Application and Sustainable Optimization

#### 5.3.1 Develop Appropriate Electrolytes and Broaden the Application Scope

The future development of electrolytes for SIBs needs to consider the synergistic innovation of both component optimization and application scenarios. In terms of electrolyte components, the Na salts need to balance high solubility, stability, low cost, and environmental friendliness, while also improving the film‐forming performance of anion‐derived SEI. The selection of solvents should give priority to high stability, wide electrochemical window, and coordination ability. Organic solvents are still the main research direction because of their comprehensive performance advantages. The additives should satisfy multi‐functions through task‐specific functional groups, and the decomposition mechanism should be strengthened to match the electrolyte system. Furthermore, the broadening of the application scope needs to pay attention to the characteristics of different electrolyte systems. Ether‐based electrolytes have excellent cycling performance but poor high‐voltage stability.^[^ ^14^ ^]^ Sulfone and nitrile solvents can form stable CEI, but their physical properties, such as ionic conductivity and viscosity, need further optimization. Meanwhile, solid electrolytes have attracted much attention because of their high safety, and the bottlenecks are their poor ionic conductivity and interfacial contact,^[^ ^354^, ^355^ ^]^ low compatibility with electrode materials, and air sensitivity.

#### 5.3.2 Sustainable Collaborative Optimization for Practical Applications

Many key challenges need to be overcome in accelerating SIBs from laboratory research to commercialization. Despite the high abundance of sodium resources, cost control remains a core challenge. Especially, the cost of synthesizing high‐purity sodium salts in electrolytes, the introduction of functional additives, and the solvent purification process limit its competitiveness in cost. Therefore, it is necessary to find a low‐cost electrolyte system and reduce electrolyte consumption through interface modification. In terms of sustainable development of batteries, it requires targeted battery recycling technologies that balance economics and environmental friendliness to reduce environmental pollution. Meanwhile, the construction of a full life cycle evaluation system for batteries is particularly important. It must quantify the energy consumption of electrolyte synthesis, the gain of interfacial modification on the cycle life of batteries, and the environmental burden of recycling of discarded batteries, so as to avoid the systematic imbalance caused by single link optimization. In the future, it is necessary to promote breakthroughs in cost, recyclability, and environmental compatibility of SIBs through the synergistic innovation of “material‐process‐policy”, and ultimately realize the closed‐loop sustainable development from the laboratory to industrialization.

Overall, the electrolyte and the interface, as a medium for transporting ions and protection of electrodes, complement each other, making them crucial in SIBs. A comprehensive understanding of the interaction and connection between different components in electrolytes and interfaces, and exploration of underlying mechanisms, will advance the topic of high‐performance SIBs and get us ready for the post‐lithium‐ion battery era.

## Conflict of Interest

The authors declare no conflict of interest.
